# Supplementary material for: Understanding the interplay between urban segregation and accessibility to services with network analysis
Source: PLoS One. 2026 Apr 1;21(4):e0342156. doi: 10.1371/journal.pone.0342156 (PMC13042880; doi:10.1371/journal.pone.0342156)
Supplement: S1 Appendix — (PDF) [file pone.0342156.s001.pdf]

# Understanding the interplay between urban segregation and accessibility to services with network analysis: Supplementary Material

## Point of Interests' OSM categories

We adopted the Points of Interest (POIs) tags classification into main categories used by Nicoletti et al. [1], after some cleaning and updates to the ever changing Open Street Map ontology.

```
categories = {
    'mobility': {
        'tags': {
            'public_transport': ['station', 'stop_position', 'platform',
                                 'stop_area', 'stop_area_group'],
            'highway': ['bus_stop'],
            'amenity': ['bus_station']
        }
    },
    'active_living': {
        'tags': {
            'leisure': ['fitness_centre', 'sports_centre', 'park', 'pitch',
                        'playground', 'swimming_pool', 'garden', 'golf_course',
                        'ice_rink', 'dog_park', 'nature_reserve', 'marina',
                        'fitness_station'],
            'landuse': ['recreation_ground', 'skatepark', 'skate_park'],
            'sport': ['skateboard'],
            'amenity': ['bicycle_parking']
        }
    },
    'entertainment': {
        'tags': {
            'amenity': ['pub', 'bar', 'theatre', 'cinema', 'nightclub',
                        'events_venue']
        }
    },
    'food': {
```

```
        'tags': {
            'amenity': ['restaurant', 'cafe', 'food_court', 'marketplace',
                        'community_centre']
        }
    },
    'community': {
        'tags': {
            'amenity': ['library', 'social_facility', 'social_centre',
                        'townhall']
        }
    },
    'education': {
        'tags': {
            'amenity': ['school', 'childcare', 'child_care', 'kindergarten',
                        'university', 'college']
        }
    },
    'health_and_wellbeing': {
        'tags': {
            'amenity': ['pharmacy', 'dentist', 'clinic', 'hospital', 'doctors']
        }
    }
}
```

# References

[1] L. Nicoletti, M. Sirenko, and T. Verma. Disadvantaged communities have lower access to urban infrastructure. *Environment and Planning B: Urban Analytics and City Science*, 50(3):831–849, 2023.
