# Supplementary material for: Understanding the interplay between urban segregation and accessibility to services with network analysis
Source: PLoS One. 2026 Apr 1;21(4):e0342156. doi: 10.1371/journal.pone.0342156 (PMC13042880; doi:10.1371/journal.pone.0342156)
Supplement: S2 Appendix — (PDF) [file pone.0342156.s002.pdf]

Understanding the interplay between urban segregation and accessibility to services with network analysis: Supplementary Material

More on cities' rankings

In this appendix, we show some additional plots that are not included in the main paper, to discuss how our measures can be used to produce other cities' rankings and analyses.

In Fig. 1, box and whiskers diagrams are drawn according the $\mathcal{P}$, $\mathcal{P}_{\text{avg}}$, and $\mathcal{A}$ dimensions for the full list of the 92 cities we analyzed. Then, we show all the cities' ranks by PoI-accessibility, and normalized closeness, with their raw data in Table 1, completing the information given in the paper. We can use this table to compare how each city has been ranked by $\mathcal{P}_{\text{avg}}$ (first column) and by $\mathcal{C}'$. All the measures shown here, as in the main paper, are aggregated by averaging the values for each city's point weighted by population at that point. It should also be observed that our analysis has been performed over 92 cities world-wide; for 11 of these cities, we were not able to get the necessary data to calculate closeness, so the bubble charts we plotted in the main paper have been created on 81 cities only.

Cities can also be ranked by the percentage of census areas with no services at all, as in Fig. 2. Here, we have a very different perspectives, where cities ranked in top positions are very low in terms of accessibility, since they show big "holes", or large urban areas with citizens that cannot access nearby services just walking by less than 60 minutes from their residential addresses.

Finally, we show what happens to cities' rankings if we change the weights $w_{\mathcal{P}}, w_{\mathcal{D}}, w_{\mathcal{E}}$ in the definition of PoI-accessibility, as in equation (1) in the main paper. We recall that in our analysis, we set $w_{\mathcal{P}} = w_{\mathcal{D}} = w_{\mathcal{E}} = 1/3$, although we already discussed how the cities' rankings change when we use only the PoI-proximity component (i.e., $w_{\mathcal{P}} = 1, w_{\mathcal{D}} = w_{\mathcal{E}} = 0$), the PoI-density component (i.e., $w_{\mathcal{P}} = 0, w_{\mathcal{D}} = 1, w_{\mathcal{E}} = 0$), or the PoI-entropy component (i.e., $w_{\mathcal{P}} = w_{\mathcal{D}} = 0, w_{\mathcal{E}} = 1$) - see Fig. 6 of the main paper. In Fig. 3 below, we show the Kendall's tau values of the correlations between different rankings based on PoI-accessibility defined in terms of a wider spectrum of variation of coefficients' values. Also observe that we keep the constraint that $w_{\mathcal{P}} + w_{\mathcal{D}} + w_{\mathcal{E}} = 1$, producing a triangle where the vertices are the specific cases where one of the dimension is dominant. All the resulting rankings are compared to the main

baseline that has been deeply analyzed in the paper. All the values are positive, showing strong or very strong signals of correlations, from a minimum of 0.55 to a maximum of 0.98 - of course we will have a value of 1 for the cell in the diagram corresponding to the baseline, i.e., $w_{\mathcal{P}} = w_{\mathcal{D}} = w_{\mathcal{E}} = 1/3$. This analysis confirms the general robustness of such rankings over different choices of the weights used to define PoI-accessibility, leaving the analyst or the stakeholder to strategically give more importance to a dimension over the others.

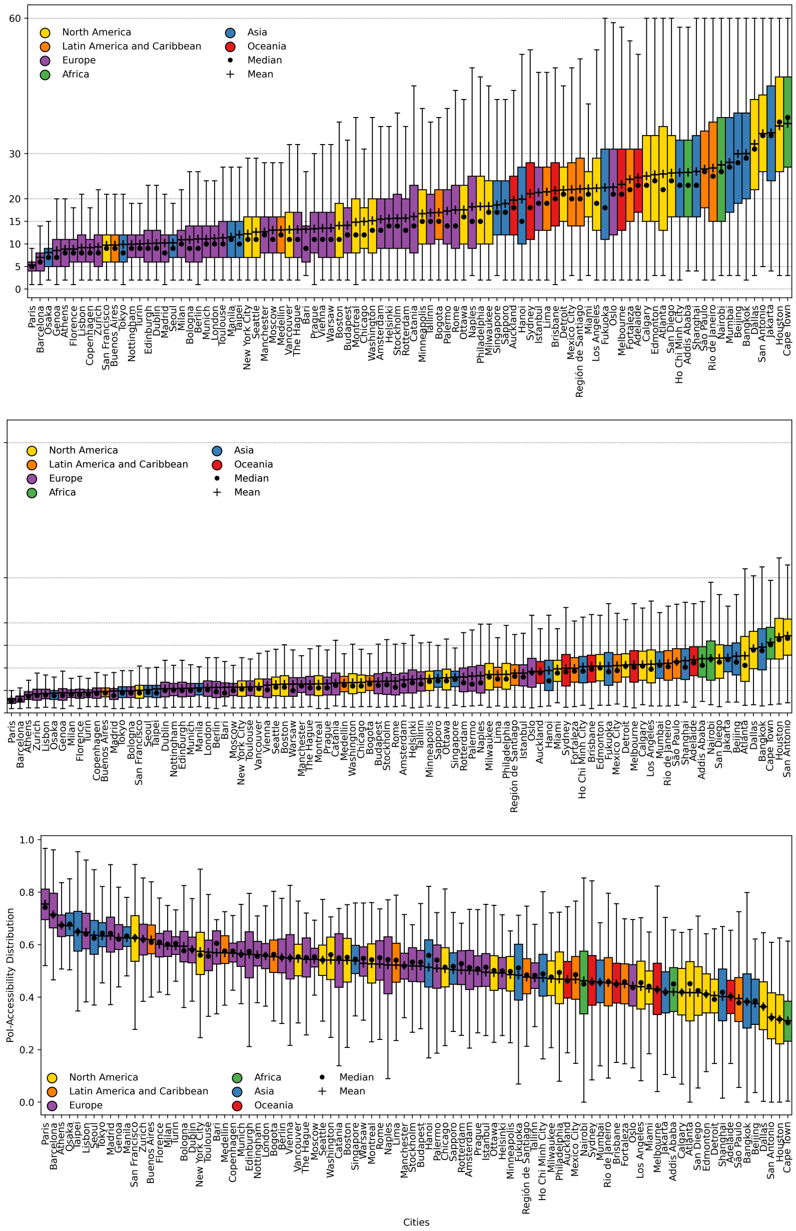

Figure 1: 92 cities ranked by average PoI-proximity $\mathcal{P}$ (top), PoI-proximity-avg $\mathcal{P}_{\text{avg}}$ (middle), PoI-accessibility $\mathcal{A}$; raw data values for each measure are weighted by population in n.

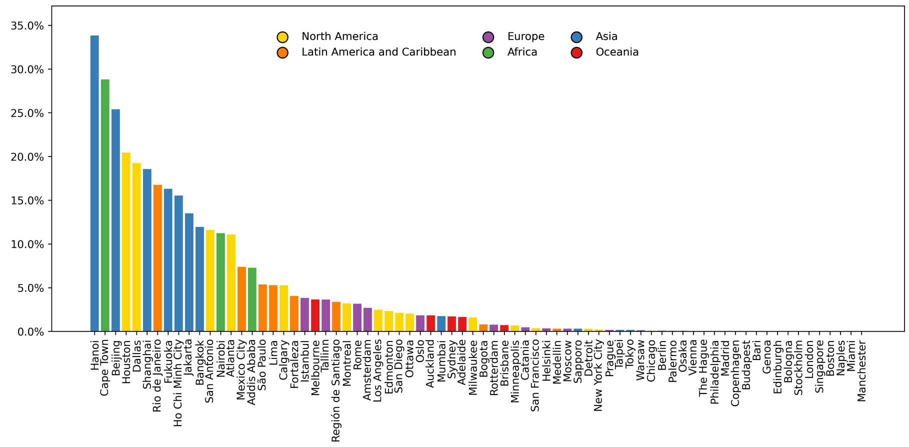

Figure 2: Cities ranked by percentage of census areas with no services, that is $\mathcal{P}(n) > 60$ minutes.

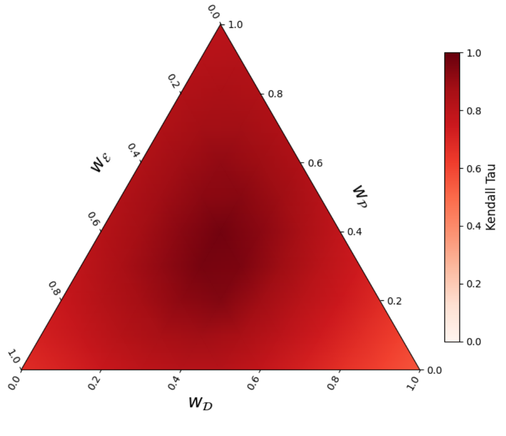

Figure 3: Variation of rankings with respect to w_1, w_2, and w_3.

Rank # by $\mathcal{A}$	City	Country	Geo. Area	Population	$\mathcal{A}$	$\mathcal{C}'$	Rank # by $\mathcal{C}'$
1	**Paris**	France	Europe	1,774,122	0.755	0.409	71
2	Barcelona	Spain	Europe	1,410,695	0.715	0.627	16
3	Athens	Greece	Europe	598,628	0.674	0.582	38
4	Osaka	Japan	Asia	2,159,341	0.672	0.289	78
5	Taipei	Taiwan	Asia	1,778,920	0.649	0.315	75
6	Lisbon	Portugal	Europe	449,355	0.648	0.443	70
7	Seoul	South Korea	Asia	6,652,249	0.636	–	–
8	Tokyo	Japan	Asia	12,280,604	0.634	0.338	74
9	Madrid	Spain	Europe	2,627,850	0.633	0.605	24
10	Genoa	Italy	Europe	462,892	0.627	0.562	47
11	Manila	Philippines	Asia	1,349,068	0.626	0.619	19
12	San Francisco	USA	North America	538,682	0.626	0.590	29
13	Zurich	Switzerland	Europe	347,358	0.622	0.540	56
14	Buenos Aires	Argentina	Latin American and Carribean	1,775,777	0.619	0.158	81
15	Florence	Italy	Europe	275,414	0.602	0.648	10
16	Milan	Italy	Europe	848,877	0.596	0.609	22
17	**Turin**	Italy	Europe	591,465	0.595	0.629	15
18	Bologna	Italy	Europe	252,104	0.593	0.622	17
19	Dublin	Ireland	Europe	447,126	0.582	0.587	30
20	New York City	USA	North America	5,410,843	0.575	0.552	53
21	Toulouse	France	Europe	318,860	0.571	0.231	80
22	Bari	Italy	Europe	213,419	0.570	0.523	59
23	Medellin	Colombia	Latin American and Carribean	2,269,152	0.569	–	–
24	Copenhagen	Denmark	Europe	503,241	0.568	0.572	41
25	Munich	Germany	Europe	1,121,268	0.565	0.339	73
26	Edinburgh	UK	Europe	367,296	0.563	0.580	39
27	Nottingham	UK	Europe	265,923	0.562	0.599	26
28	London	UK	Europe	6,334,662	0.557	0.603	25
29	Bogota	Colombia	Latin American and Carribean	8,186,793	0.555	0.315	76
30	Berlin	Germany	Europe	2,197,778	0.551	0.648	9
31	Vienna	Austria	Europe	1,180,615	0.549	0.594	28
32	**Vancouver**	Canada	North America	482,450	0.547	0.675	6
33	The Hague	Netherlands	Europe	370,290	0.545	0.546	54
34	Moscow	Russia	Europe	8,869,256	0.545	–	–
35	Seattle	USA	North America	494,965	0.542	0.621	18
36	Washington	USA	North America	506,391	0.542	0.568	43
37	Catania	Italy	Europe	211,714	0.541	0.507	61
38	Boston	USA	North America	480,598	0.540	0.555	51
39	Singapore	Singapore	Asia	3,145,249	0.538	0.372	72
40	Warsaw	Poland	Europe	1,157,328	0.529	0.676	5
41	Montreal	Canada	North America	1,134,902	0.527	0.607	23
42	Rome	Italy	Europe	1,721,192	0.526	0.554	52
43	Naples	Italy	Europe	592,770	0.523	0.455	69
44	Lima	Perù	Unknown	7,485,912	0.522	–	–
45	Manchester	UK	Europe	477,632	0.521	0.310	77
46	Stockholm	Sweden	Europe	673,379	0.519	0.560	48
47	Budapest	Hungary	Europe	874,101	0.519	0.583	37
48	Hanoi	Vietnam	Asia	3,243,512	0.514	–	–
49	Palermo	Italy	Europe	390,377	0.510	0.570	42
50	Chicago	USA	North America	1,714,484	0.508	0.633	13
51	Sapporo	Japan	Asia	1,345,080	0.505	0.465	67
52	Amsterdam	Netherlands	Europe	490,085	0.504	0.539	57
53	Rotterdam	Netherlands	Europe	248,243	0.503	0.559	49
54	Prague	Czechia	Europe	881,201	0.500	0.631	14
55	Istanbul	Turkey	Europe	10,140,077	0.495	0.691	4
56	**Ottawa**	Canada	North America	620,190	0.493	0.587	31
57	Helsinki	Finland	Europe	459,721	0.492	0.488	63
58	Minneapolis	USA	North America	267,687	0.486	0.649	8
59	Fukuoka	Japan	Asia	2,560,460	0.484	0.615	21
60	Santiago	Chile	Latin American and Carribean	5,040,510	0.477	0.584	34
61	Tallinn	Estonia	Europe	259,497	0.474	0.664	7
62	Ho Chi Minh City	Vietnam	Asia	7,142,622	0.473	–	–
63	Milwaukee	USA	North America	407,133	0.471	0.637	12
64	Philadelphia	USA	North America	1,081,840	0.469	0.584	35
65	Auckland	New Zealand	Oceania	786,864	0.468	0.616	20
66	Mexico City	Mexico	North America	6,039,358	0.468	0.583	36
67	Nairobi	Kenya	Africa	3,002,541	0.462	0.597	27
68	Sydney	Australia	Oceania	2,216,174	0.460	0.478	66
69	Mumbai	India	Asia	5,475,916	0.457	–	–
70	Rio de Janeiro	Brazil	Latin American and Carribean	3,474,827	0.455	0.722	1
71	Brisbane	Australia	Oceania	583,367	0.451	0.566	45
72	Fortaleza	Brazil	Latin American and Carribean	2,158,359	0.450	0.567	44
73	Oslo	Norway	Europe	516,686	0.444	0.479	65
74	Los Angeles	USA	North America	2,010,709	0.440	0.558	50
75	Miami	USA	North America	305,659	0.433	0.643	11
76	**Melbourne**	Australia	Oceania	2,268,006	0.428	0.587	32
77	Jakarta	Indonesia	Asia	6,558,981	0.421	0.699	2
78	Addis Ababa	Ethiopia	Africa	2,514,440	0.420	0.694	3
79	Calgary	Canada	North America	748,036	0.418	0.497	62
80	Atlanta, Georgia	USA	North America	211,539	0.418	0.463	68
81	San Diego	USA	North America	747,736	0.417	0.575	40
82	Edmonton	Canada	North America	542,021	0.411	0.543	55
83	Detroit	USA	North America	327,975	0.406	0.565	46
84	Shanghai	China	Asia	5,104,955	0.402	–	–
85	Adelaide	Australia	Oceania	623,012	0.401	0.255	79
86	São Paulo	Brazil	Latin American and Carribean	8,143,238	0.395	–	–
87	Bangkok	Thailand	Asia	5,873,869	0.382	0.481	64
88	Beijing	China	Asia	4,915,833	0.376	–	–
89	Dallas	USA	North America	528,646	0.364	0.513	60
90	San Antonio	USA	North America	604,793	0.324	0.586	33
91	**Houston**	USA	North America	982,723	0.315	0.536	58
92	Cape Town	South Africa	Africa	2,531,147	0.310	–	–

Table 1: Cities sorted by PoI-accessibility
