# Supplementary material for: Understanding the interplay between urban segregation and accessibility to services with network analysis
Source: PLoS One. 2026 Apr 1;21(4):e0342156. doi: 10.1371/journal.pone.0342156 (PMC13042880; doi:10.1371/journal.pone.0342156)
Supplement: S3 Appendix — (PDF) [file pone.0342156.s003.pdf]

Understanding the interplay between urban segregation and accessibility to services with network analysis: Supplementary Material

Distributions of $\mathcal{P}(n)$, $\mathcal{D}(15, n)$, $\mathcal{E}(15, n)$, and $\mathcal{A}(15, n)$ for all the other cities

We include in this appendix the distributions of our proximity, density, entropy, and accessibility metrics for all the 92 cities but the 6 highlighted in the paper.

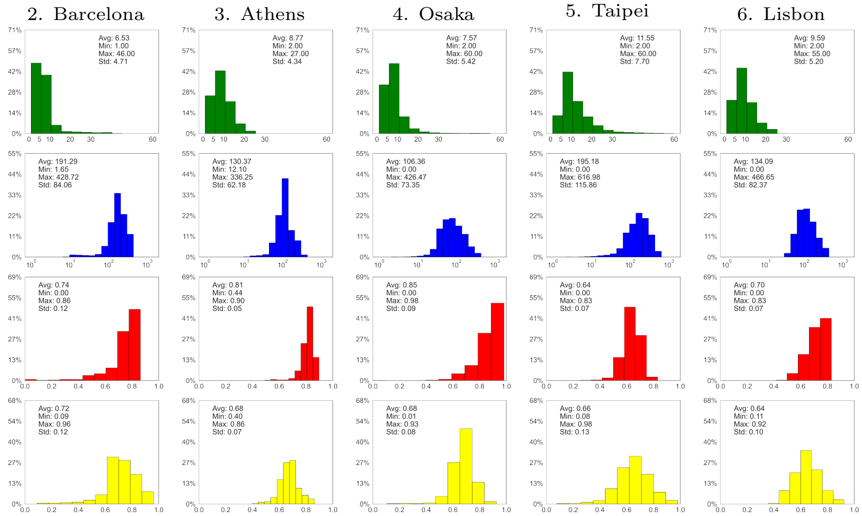

Figure 1: Distributions of $\mathcal{P}(n)$, $\mathcal{D}(15, n)$, $\mathcal{E}(15, n)$, and $\mathcal{A}(15, n)$ for cities ranked from 2 to 6 (Paris not included).

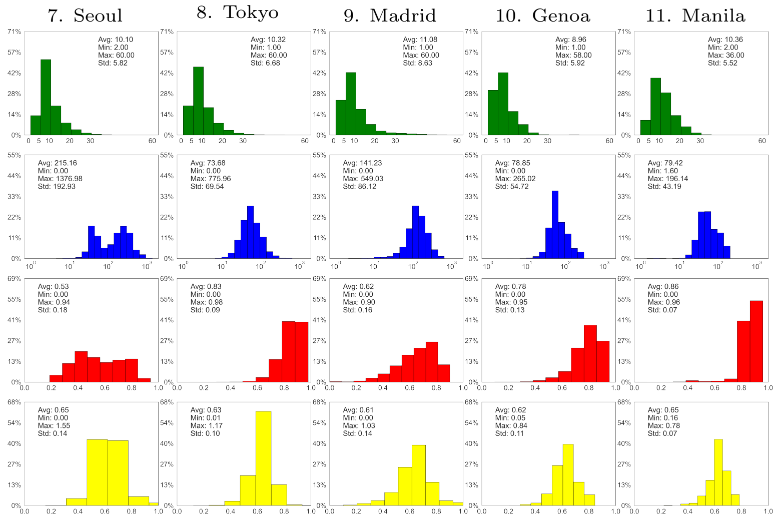

Figure 2: Distributions of $\mathcal{P}(n)$, $\mathcal{D}(15, n)$, $\mathcal{E}(15, n)$, and $\mathcal{A}(15, n)$ for cities ranked from 7 to 11.

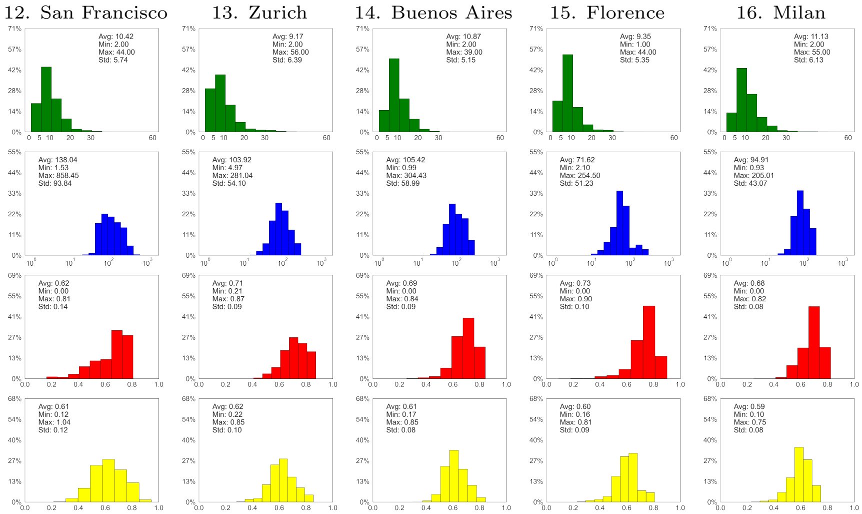

Figure 3: Distributions of $\mathcal{P}(n)$, $\mathcal{D}(15, n)$, $\mathcal{E}(15, n)$, and $\mathcal{A}(15, n)$ for cities ranked from 12 to 16 (Turin not included).

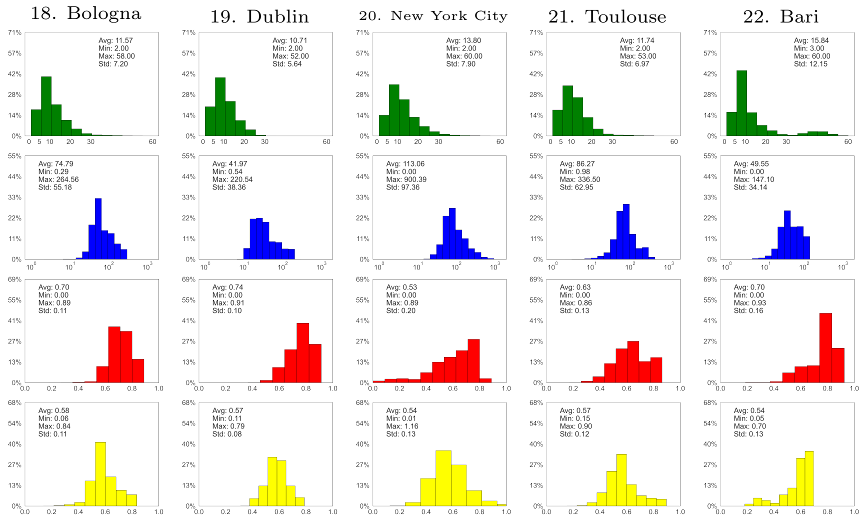

Figure 4: Distributions of $\mathcal{P}(n)$, $\mathcal{D}(15, n)$, $\mathcal{E}(15, n)$, and $\mathcal{A}(15, n)$ for cities ranked from 18 to 22.

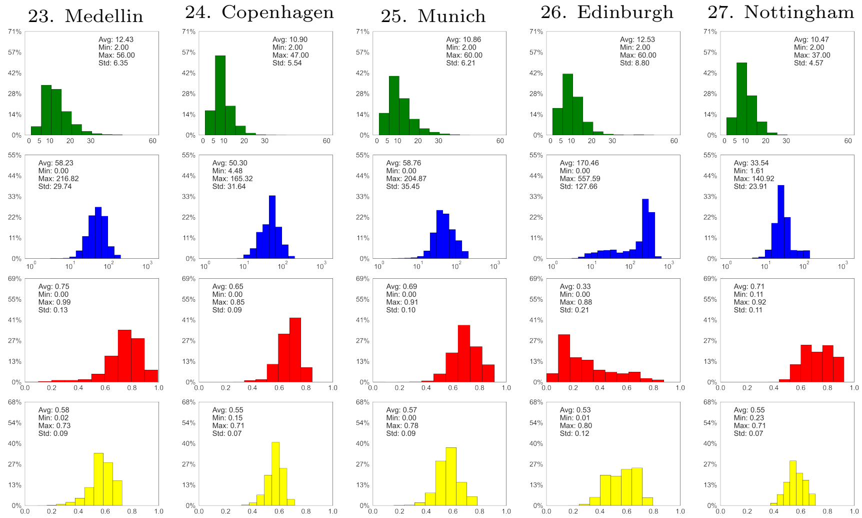

Figure 5: Distributions of $\mathcal{P}(n)$, $\mathcal{D}(15, n)$, $\mathcal{E}(15, n)$, and $\mathcal{A}(15, n)$ for cities ranked from 23 to 27.

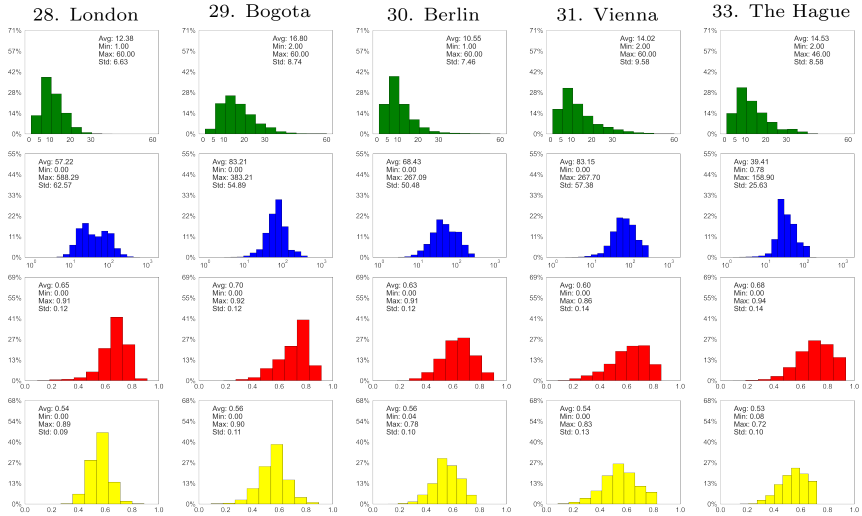

Figure 6: Distributions of $\mathcal{P}(n)$, $\mathcal{D}(15, n)$, $\mathcal{E}(15, n)$, and $\mathcal{A}(15, n)$ for cities ranked from 28 to 31, and 33 (Vancouver not included).

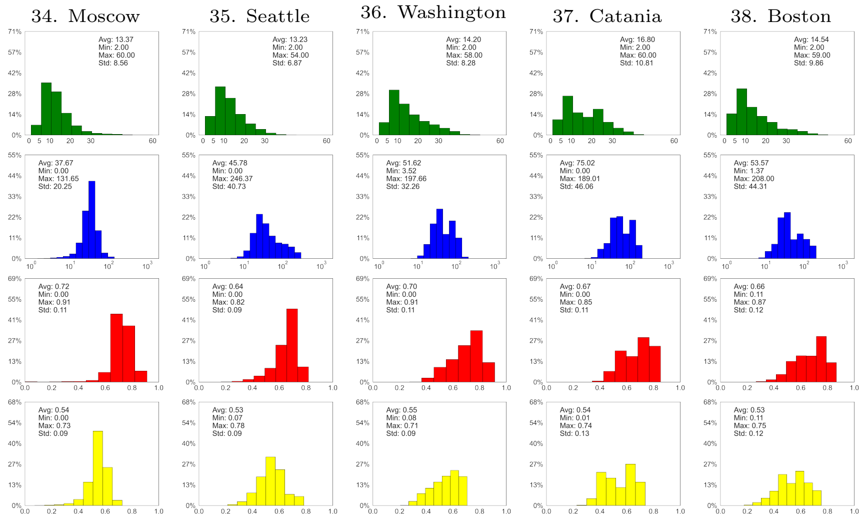

Figure 7: Distributions of $\mathcal{P}(n)$, $\mathcal{D}(15, n)$, $\mathcal{E}(15, n)$, and $\mathcal{A}(15, n)$ for cities ranked from 34 to 38.

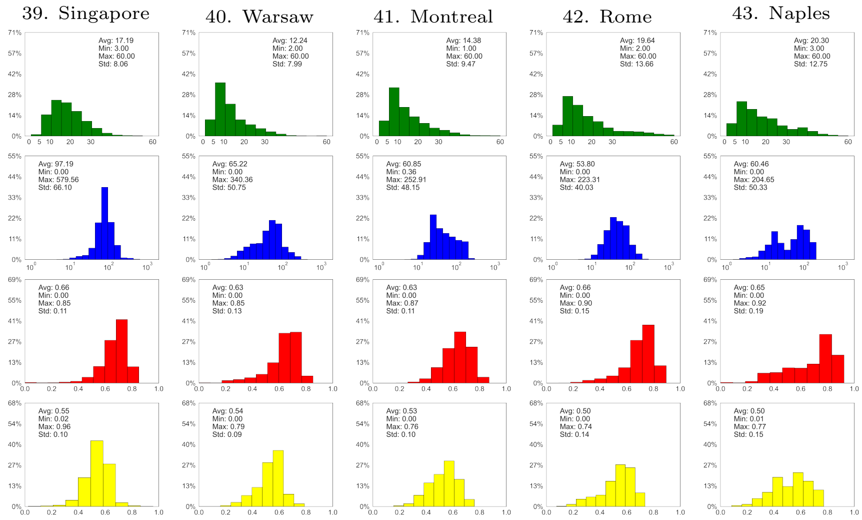

Figure 8: Distributions of $\mathcal{P}(n)$, $\mathcal{D}(15, n)$, $\mathcal{E}(15, n)$, and $\mathcal{A}(15, n)$ for cities ranked from 39 to 43.

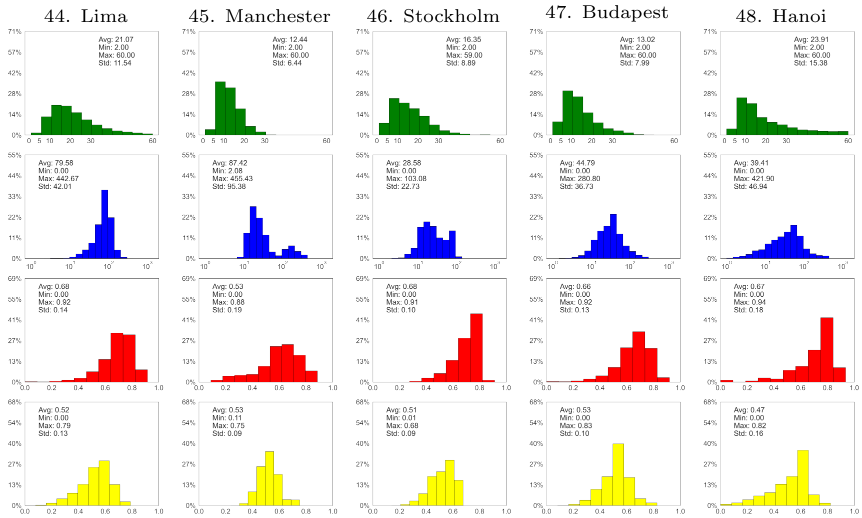

Figure 9: Distributions of $\mathcal{P}(n)$, $\mathcal{D}(15, n)$, $\mathcal{E}(15, n)$, and $\mathcal{A}(15, n)$ for cities ranked from 44 to 48.

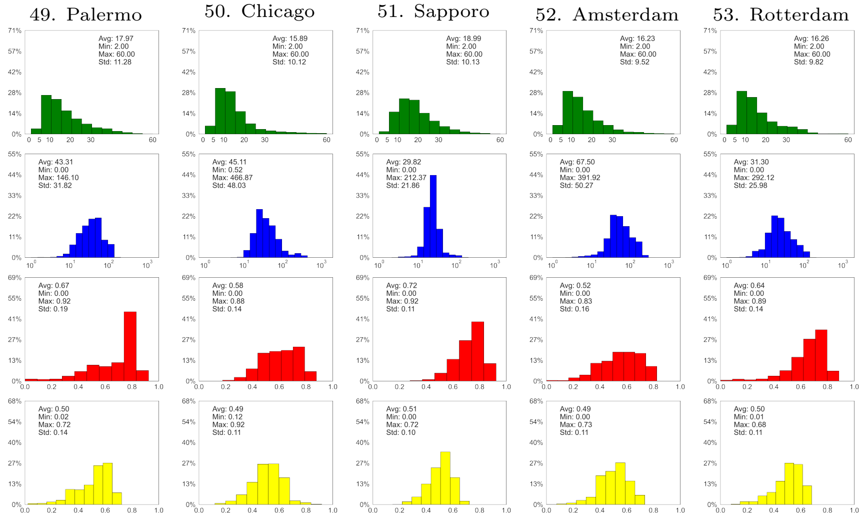

Figure 10: Distributions of $\mathcal{P}(n)$, $\mathcal{D}(15, n)$, $\mathcal{E}(15, n)$, and $\mathcal{A}(15, n)$ for cities ranked from 49 to 53.

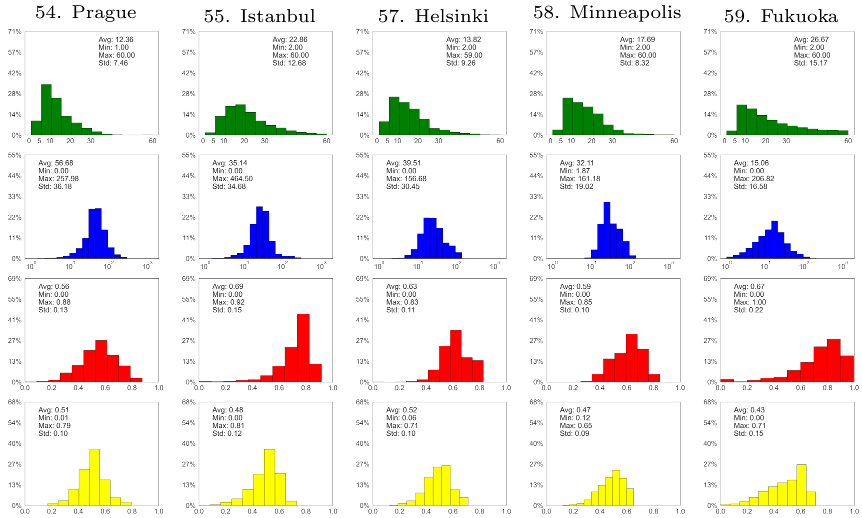

Figure 11: Distributions of $\mathcal{P}(n)$, $\mathcal{D}(15, n)$, $\mathcal{E}(15, n)$, and $\mathcal{A}(15, n)$ for cities ranked from 54 to 55, and from 57 to 59 (Ottawa not included).

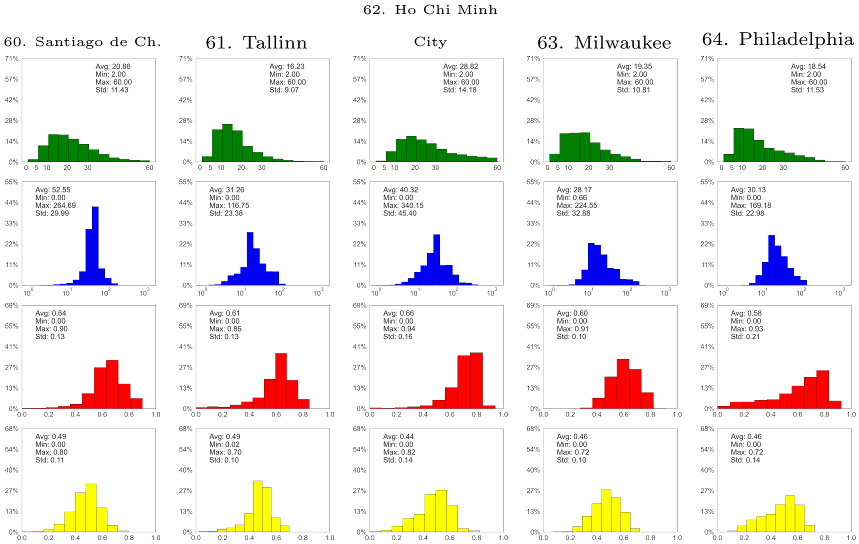

Figure 12: Distributions of $\mathcal{P}(n)$, $\mathcal{D}(15, n)$, $\mathcal{E}(15, n)$, and $\mathcal{A}(15, n)$ for cities ranked from 60 to 64.

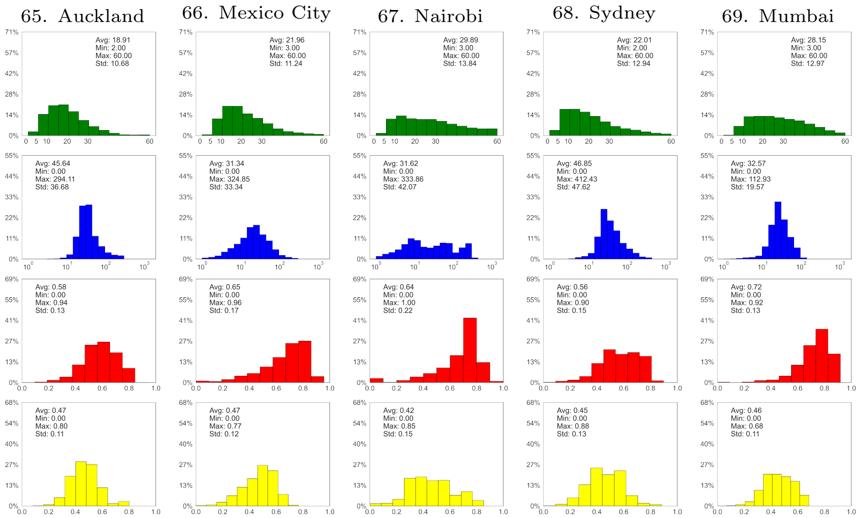

Figure 13: Distributions of $\mathcal{P}(n)$, $\mathcal{D}(15, n)$, $\mathcal{E}(15, n)$, and $\mathcal{A}(15, n)$ for cities ranked from 65 to 69.

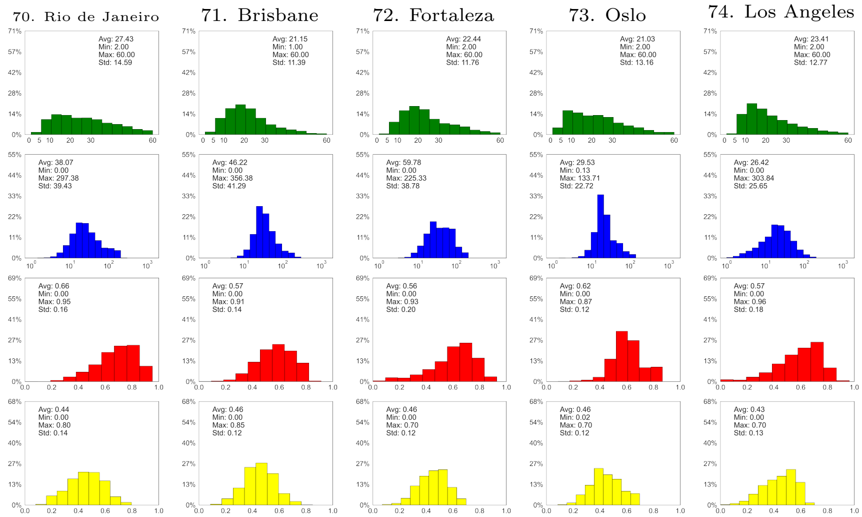

Figure 14: Distributions of $\mathcal{P}(n)$, $\mathcal{D}(15,n)$, $\mathcal{E}(15,n)$, and $\mathcal{A}(15,n)$ for cities ranked from 70 to 74.

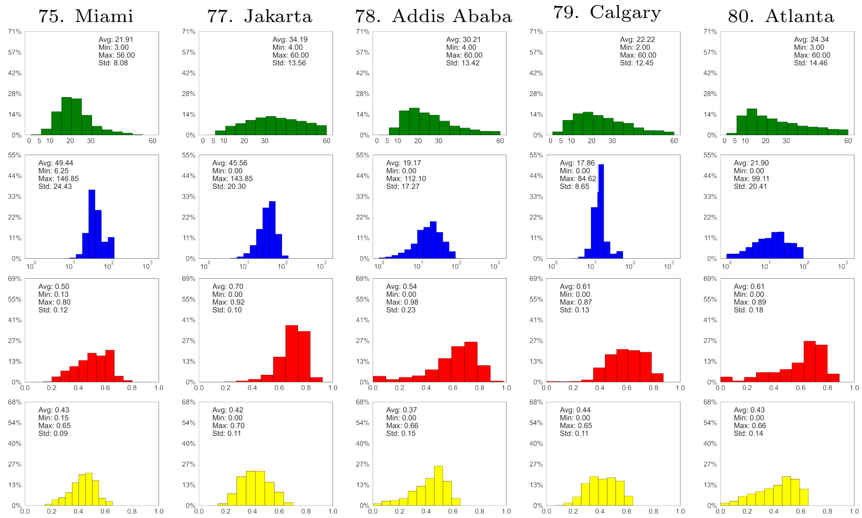

Figure 15: Distributions of $\mathcal{P}(n)$, $\mathcal{D}(15,n)$, $\mathcal{E}(15,n)$, and $\mathcal{A}(15,n)$ for cities ranked from 75 to 80 (Melbourne - 76 - not included).

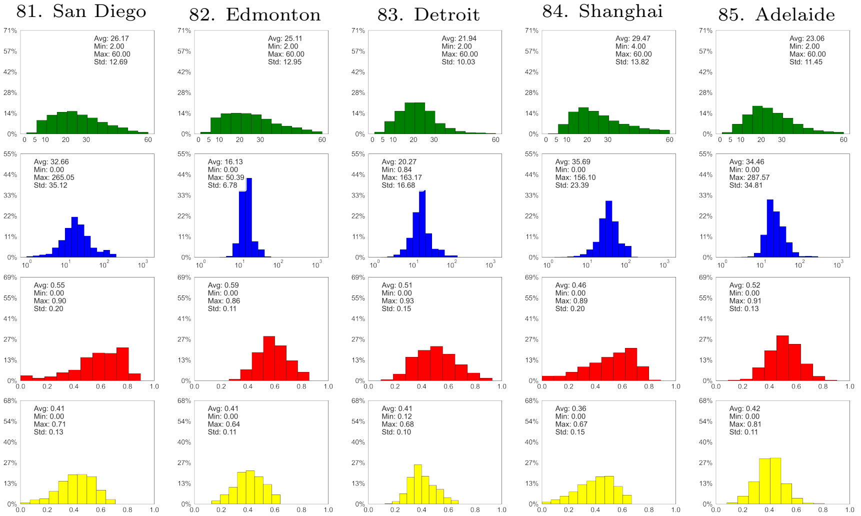

Figure 16: Distributions of $\mathcal{P}(n)$, $\mathcal{D}(15,n)$, $\mathcal{E}(15,n)$, and $\mathcal{A}(15,n)$ for cities ranked from 81 to 85.

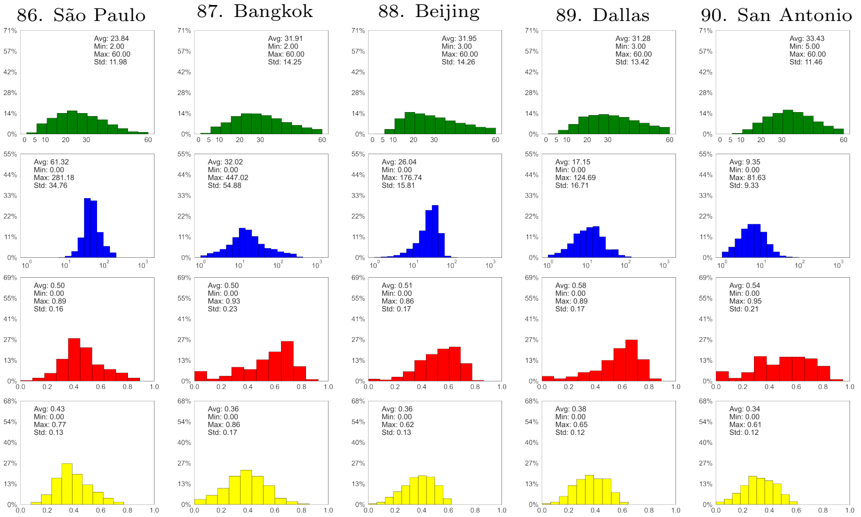

Figure 17: Distributions of $\mathcal{P}(n)$, $\mathcal{D}(15,n)$, $\mathcal{E}(15,n)$, and $\mathcal{A}(15,n)$ for cities ranked from 86 to 90 (Houston not included).

92. Cape Town

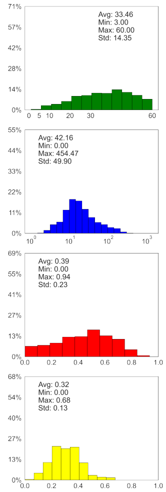

Figure 18: Distributions of $\mathcal{P}(n)$, $\mathcal{D}(15,n)$, $\mathcal{E}(15,n)$, and $\mathcal{A}(15,n)$ for city ranked 92.
