# Supplementary material for: Understanding the interplay between urban segregation and accessibility to services with network analysis
Source: PLoS One. 2026 Apr 1;21(4):e0342156. doi: 10.1371/journal.pone.0342156 (PMC13042880; doi:10.1371/journal.pone.0342156)
Supplement: S4 Appendix — (PDF) [file pone.0342156.s004.pdf]

Understanding the interplay between urban segregation and accessibility to services with network analysis: Supplementary Material

Cities' bubble charts by geographical region

In this appendix we show the same bubble chart we used in the manuscript (Fig. 10) to compare PoI-accessibility and normalized closeness in our 81 cities. However, every plot here highlights only those cities that belong to a given geographical region, i.e., USA, North America (USA non included), Latin America and Caribbean, Northern Europe, Eastern Europe, Southern Europe, Western Europe, Africa, Asia, Oceania. These plots also show how some geographical region is heavily under represented in our data.

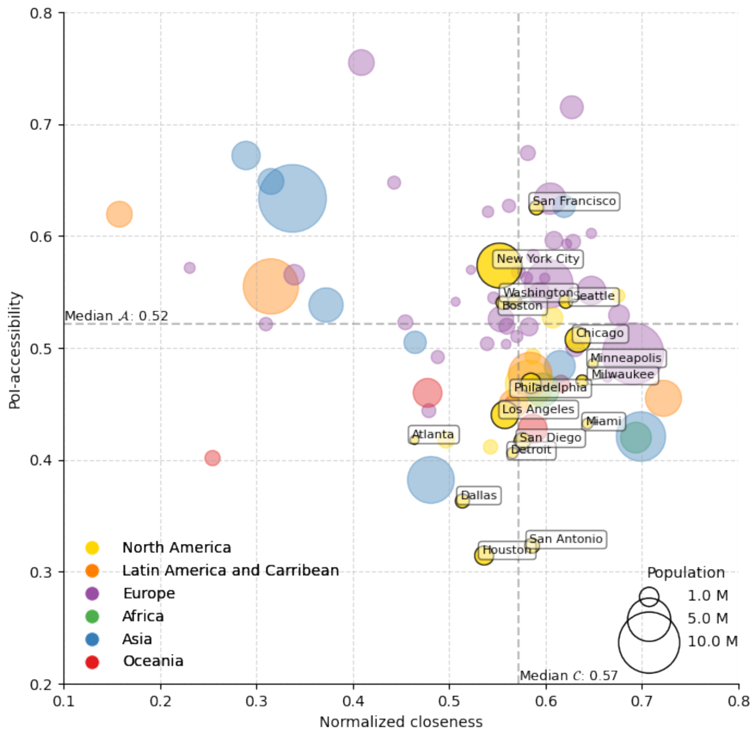

Figure 1: Cities in the USA

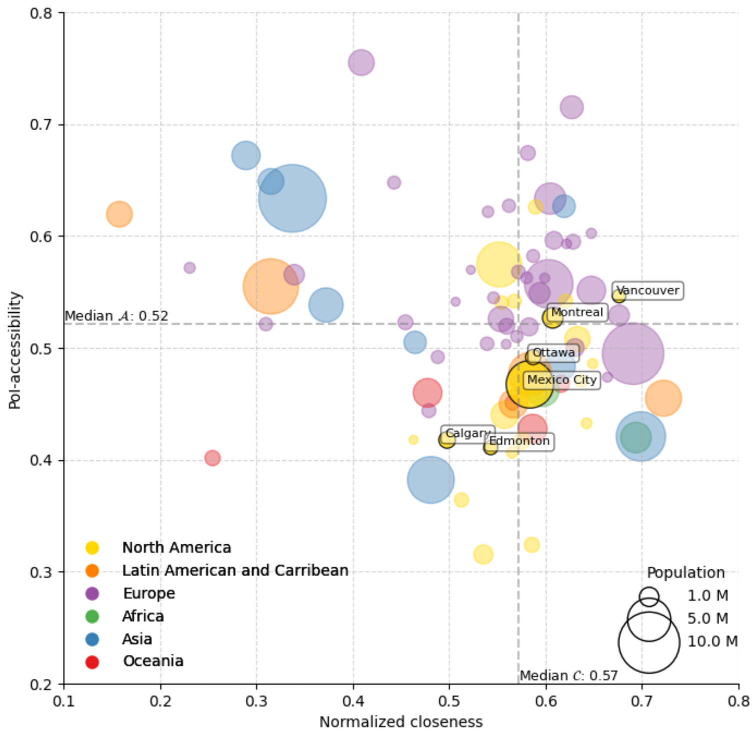

Figure 2: Cities in North America (USA not included)

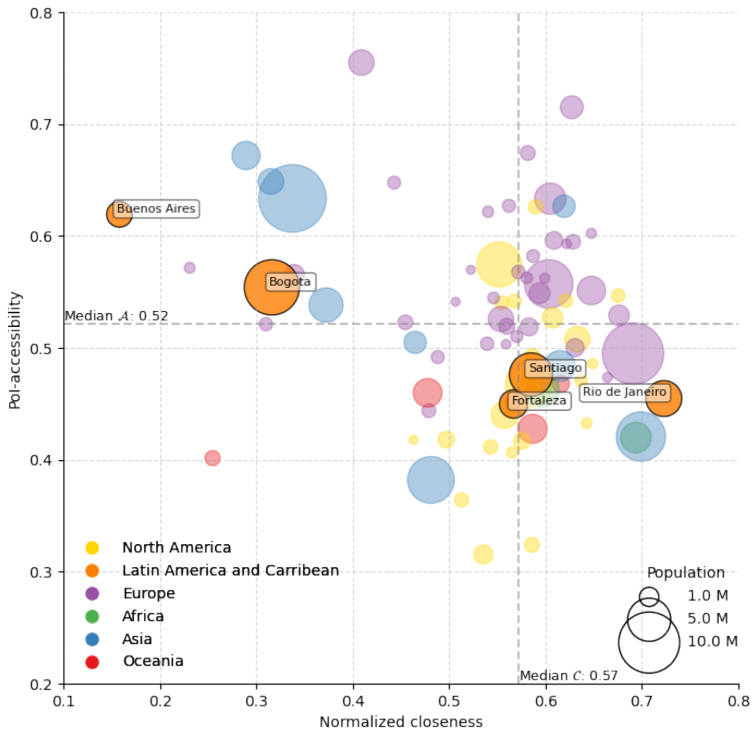

Figure 3: Cities in Latin America and Caribbean

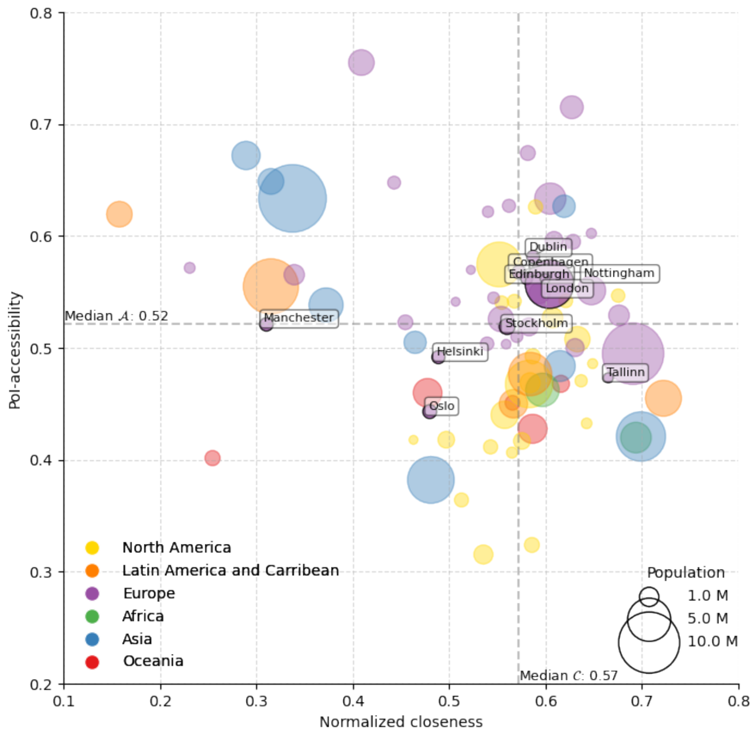

Figure 4: Cities in Northern Europe

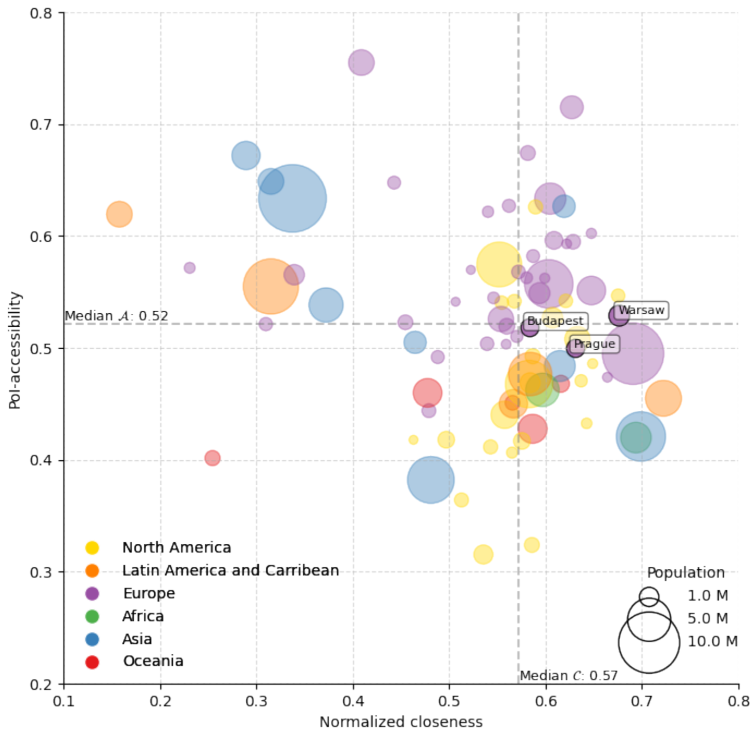

Figure 5: Cities in Eastern Europe

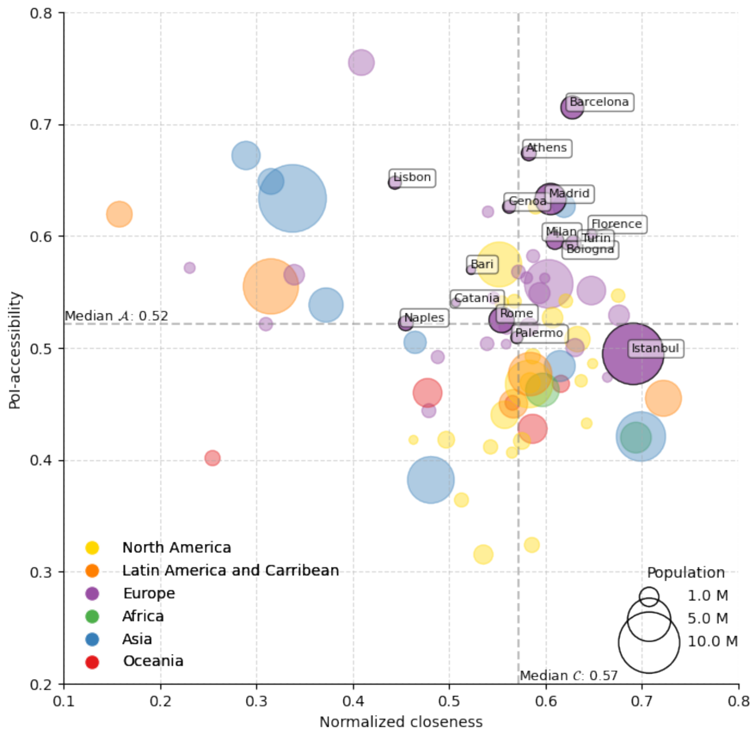

Figure 6: Cities in Southern Europe

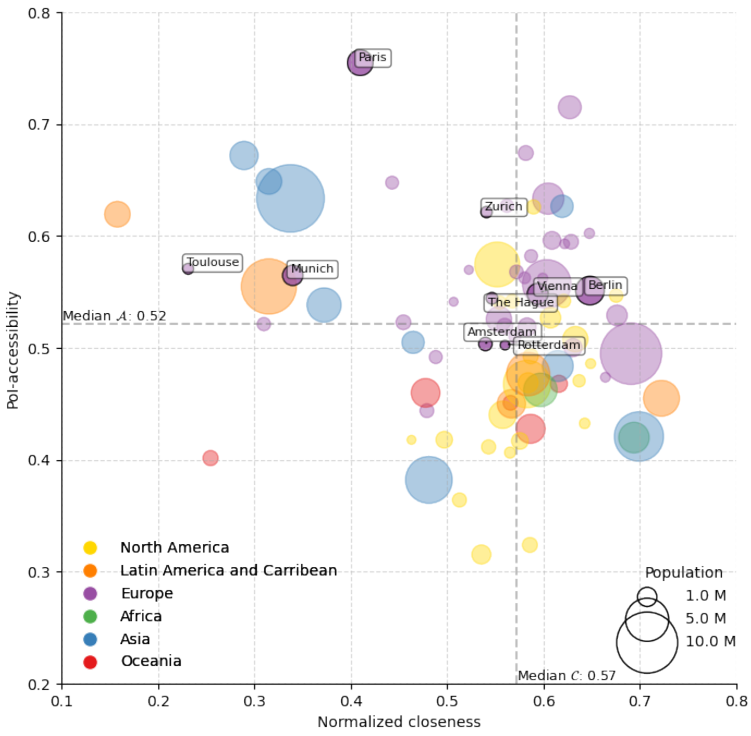

Figure 7: Cities in Western Europe

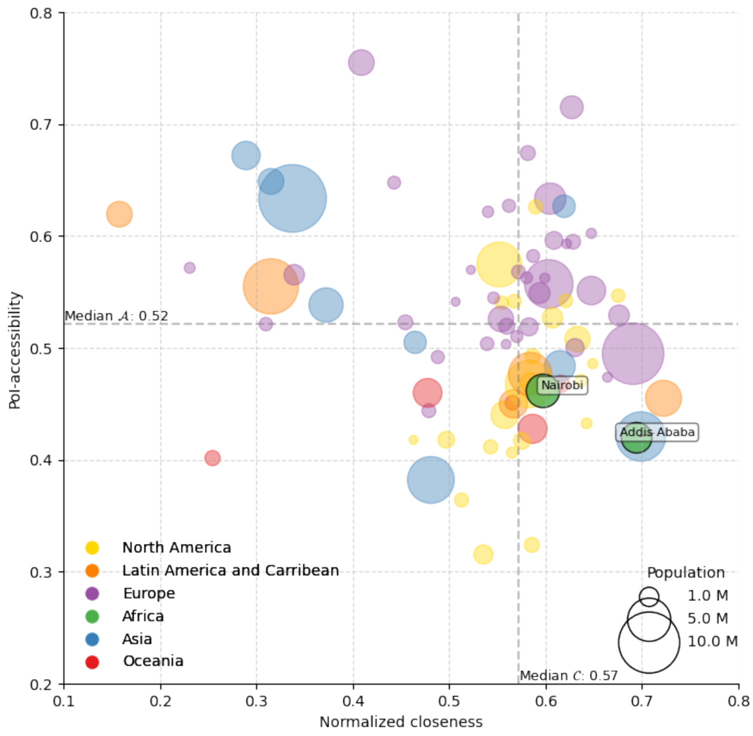

Figure 8: Cities in Africa

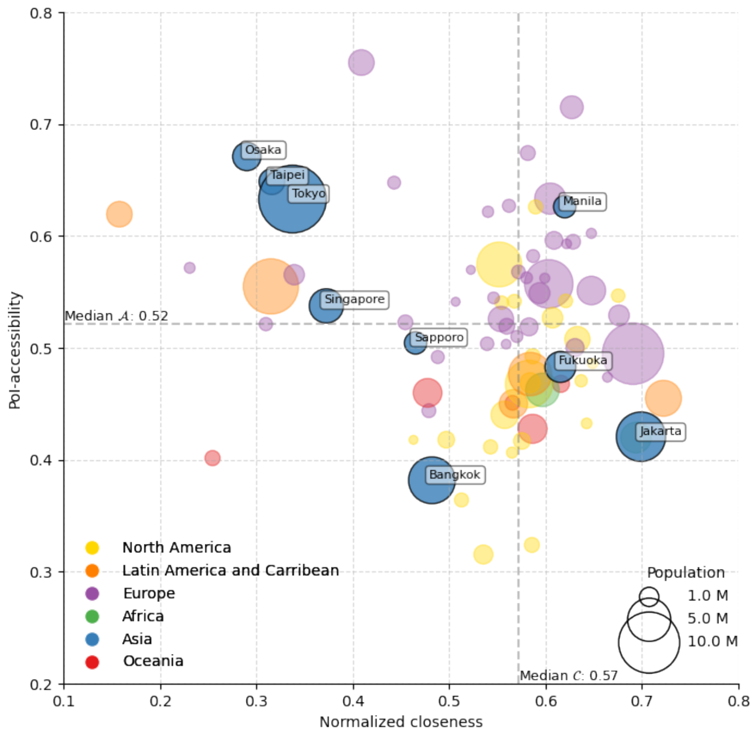

Figure 9: Cities in Asia

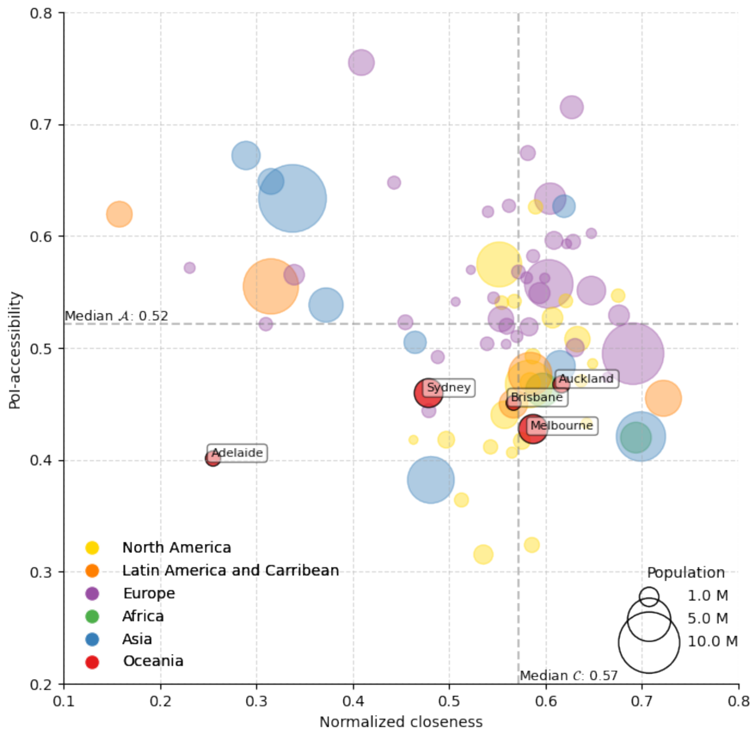

Figure 10: Cities in Oceania
