# Supplementary material for: Understanding the interplay between urban segregation and accessibility to services with network analysis
Source: PLoS One. 2026 Apr 1;21(4):e0342156. doi: 10.1371/journal.pone.0342156 (PMC13042880; doi:10.1371/journal.pone.0342156)
Supplement: S5 Appendix — (PDF) [file pone.0342156.s005.pdf]

Understanding the interplay between urban segregation and accessibility to services with network analysis: Supplementary Material

Plots on the 5 representative cities at a greater resolution

In this appendix we show at a greater resolutions some plots regarding the analyses performed on the 5 out of 6 sample cities we discussed in the paper (Turin is reported in S6 Appendix, together with the other Italian cities, so that we can display also the income's data).

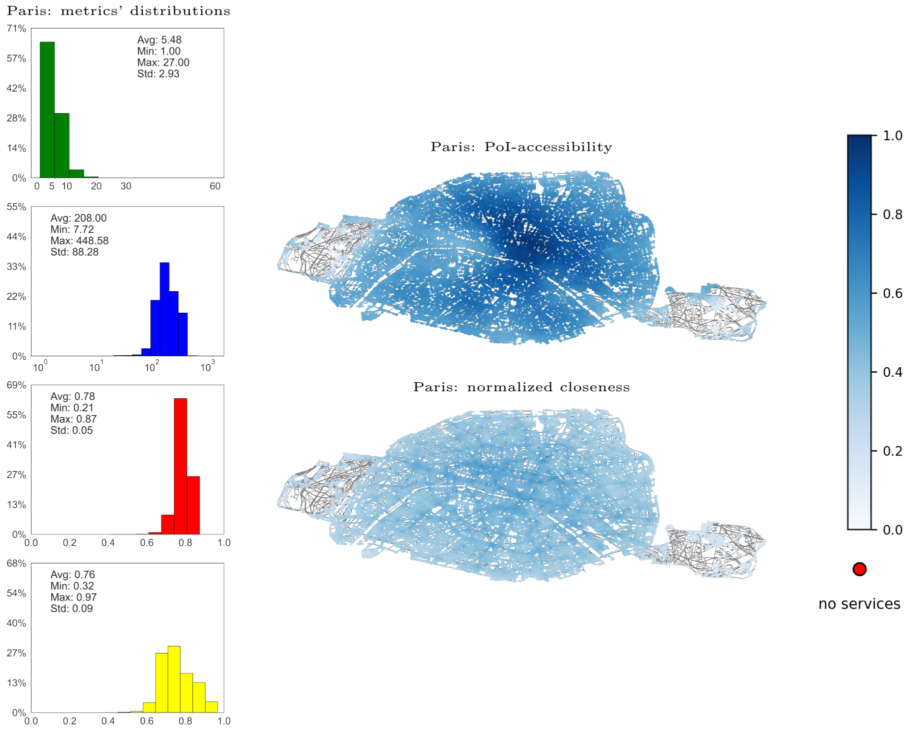

Figure 1: Paris: metrics distributions (left) and PoI-accessibility (top right) and normalized closeness (bottom right) heat maps. Base maps and data from OpenStreetMap and OpenStreetMap Foundation.

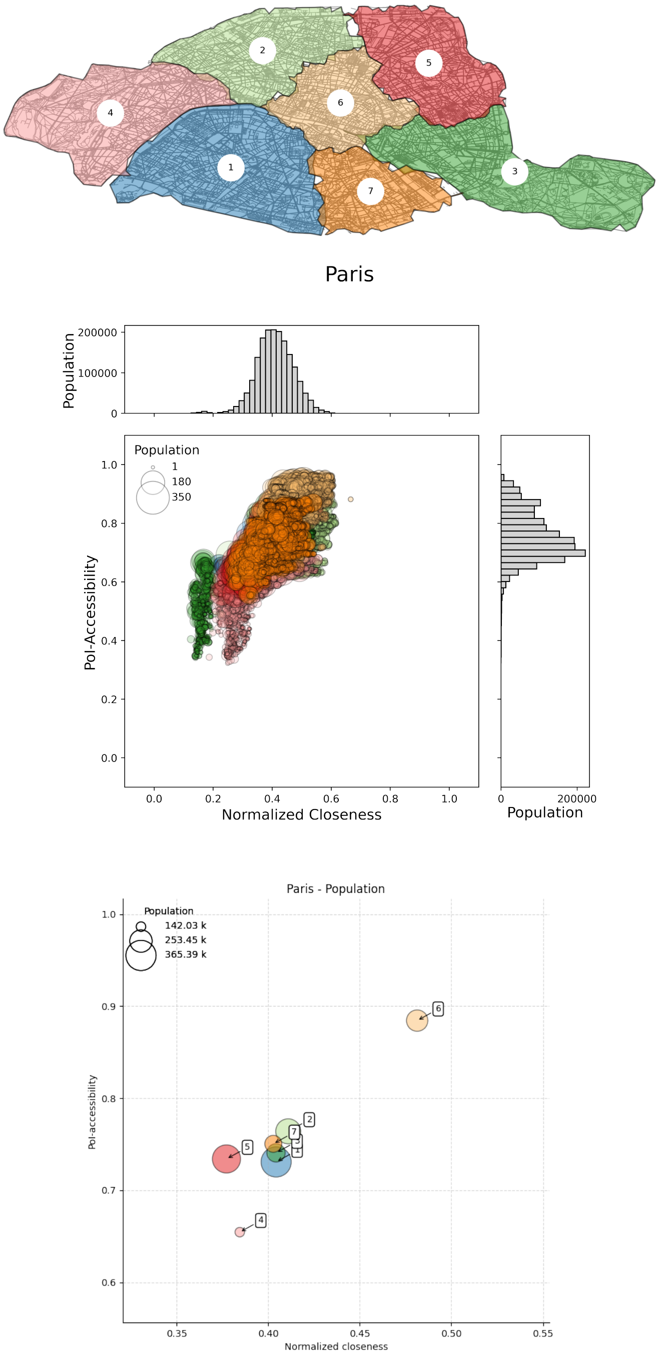

Figure 2: Paris: Infomap neighborhoods (top), PoI-accessibility vs normalized closeness aggregated by census areas (center), and neighborhoods (bottom). The maps contain information from OpenStreetMap and OpenStreetMap Foundation, which is made available under the Open Database License.

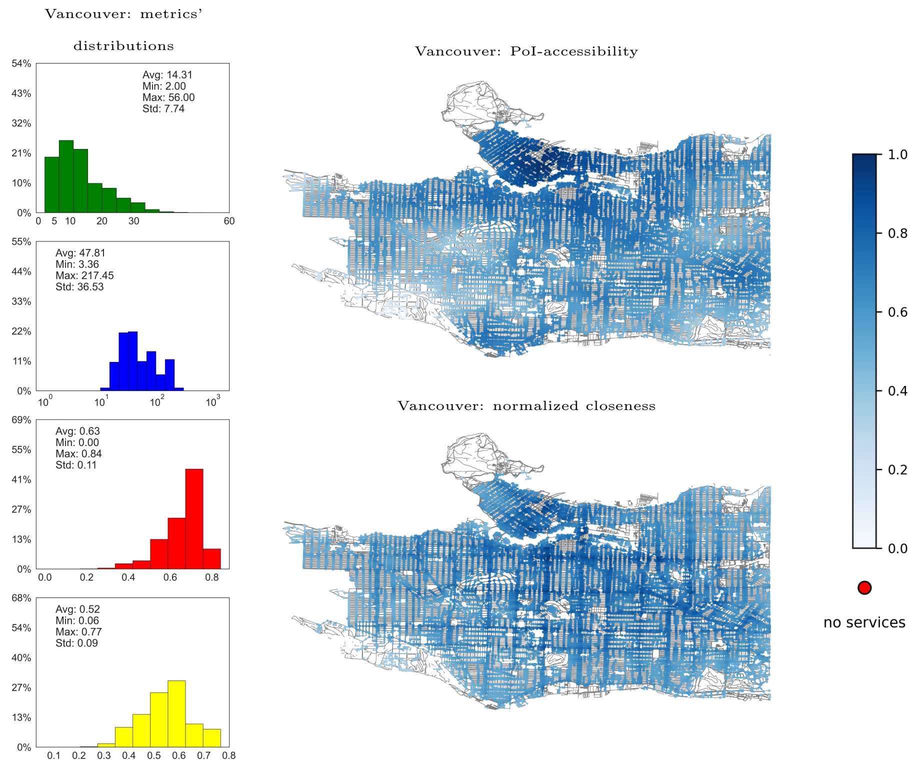

Figure 3: Vancouver: metrics distributions (left) and PoI-accessibility (top right) and normalized closeness (bottom right) heat maps. Base maps and data from OpenStreetMap and OpenStreetMap Foundation.

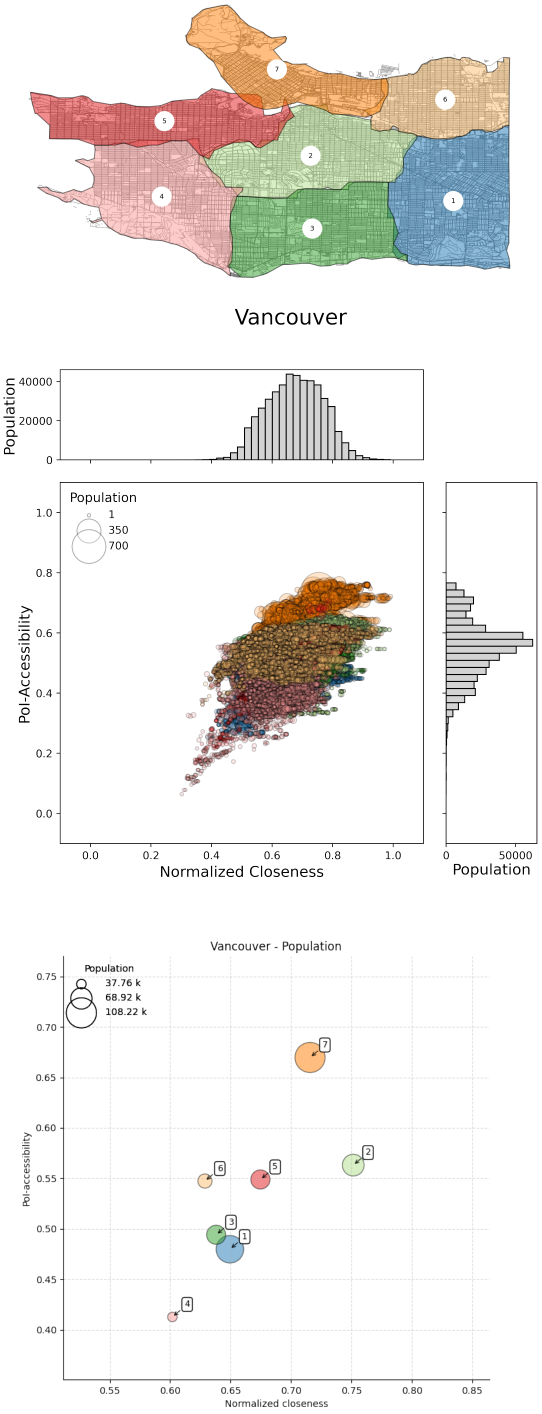

Figure 4: Vancouver: Infomap neighborhoods (top), PoI-accessibility vs normalized closeness aggregated by census areas (center), and neighborhoods (bottom). The maps contain information from OpenStreetMap and OpenStreetMap Foundation, which is made available under the Open Database License.

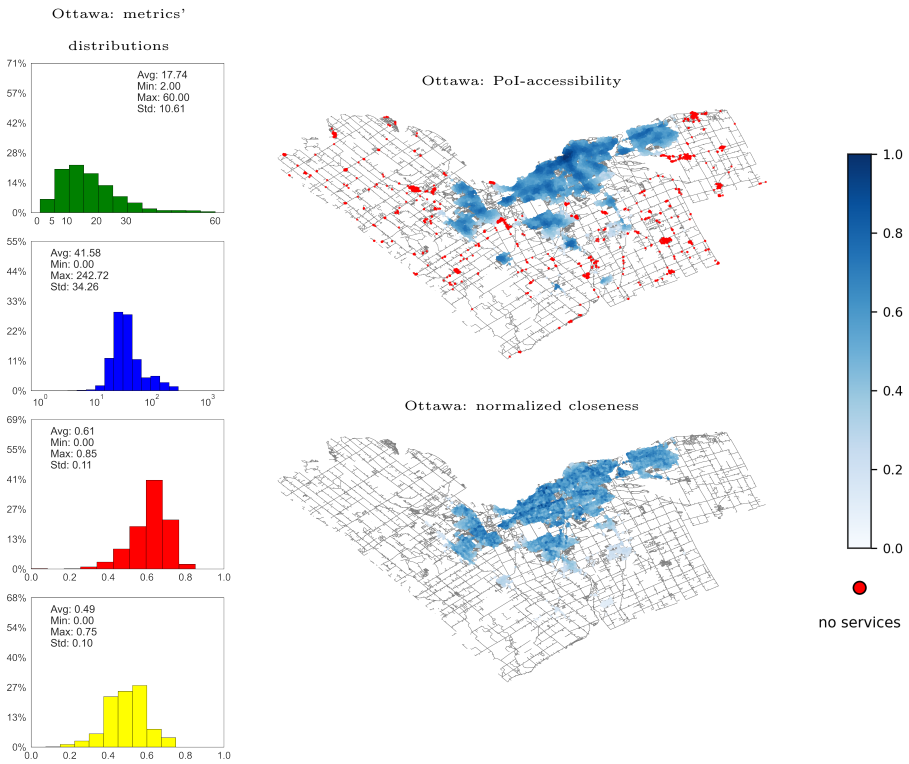

Figure 5: Ottawa: metrics distributions (left) and PoI-accessibility (top right) and normalized closeness (bottom right) heat maps. Base maps and data from OpenStreetMap and OpenStreetMap Foundation.

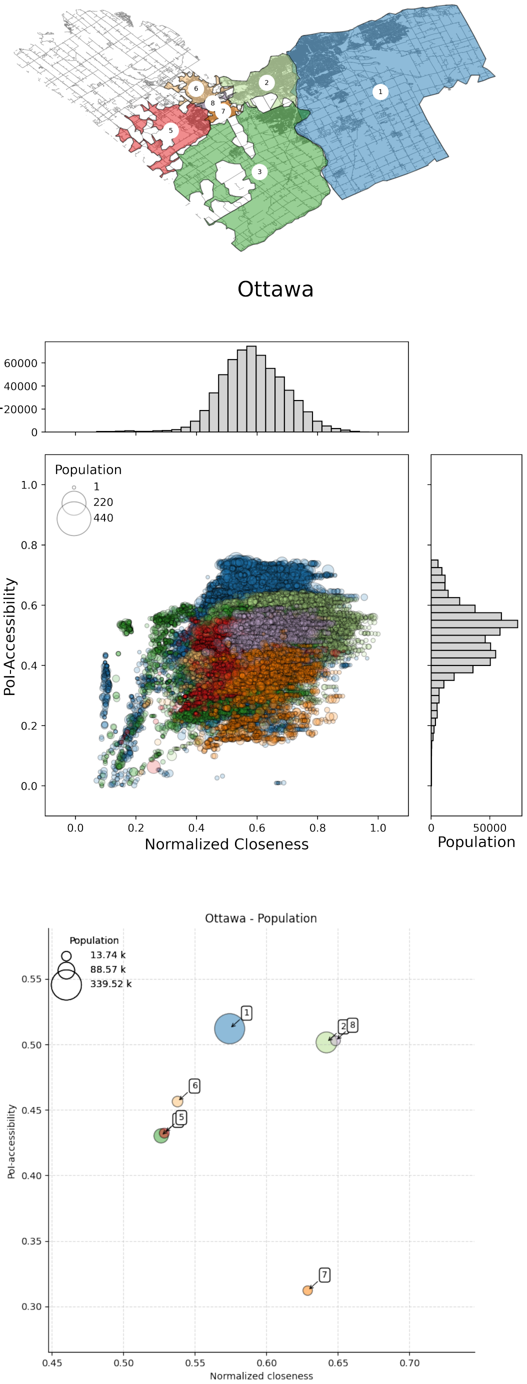

Figure 6: Ottawa: Infomap neighborhoods (top), PoI-accessibility vs normalized closeness aggregated by census areas (center), and neighborhoods (bottom). The maps contain information from OpenStreetMap and OpenStreetMap Foundation, which is made available under the Open Database License. Base maps and data from OpenStreetMap and OpenStreetMap Foundation.

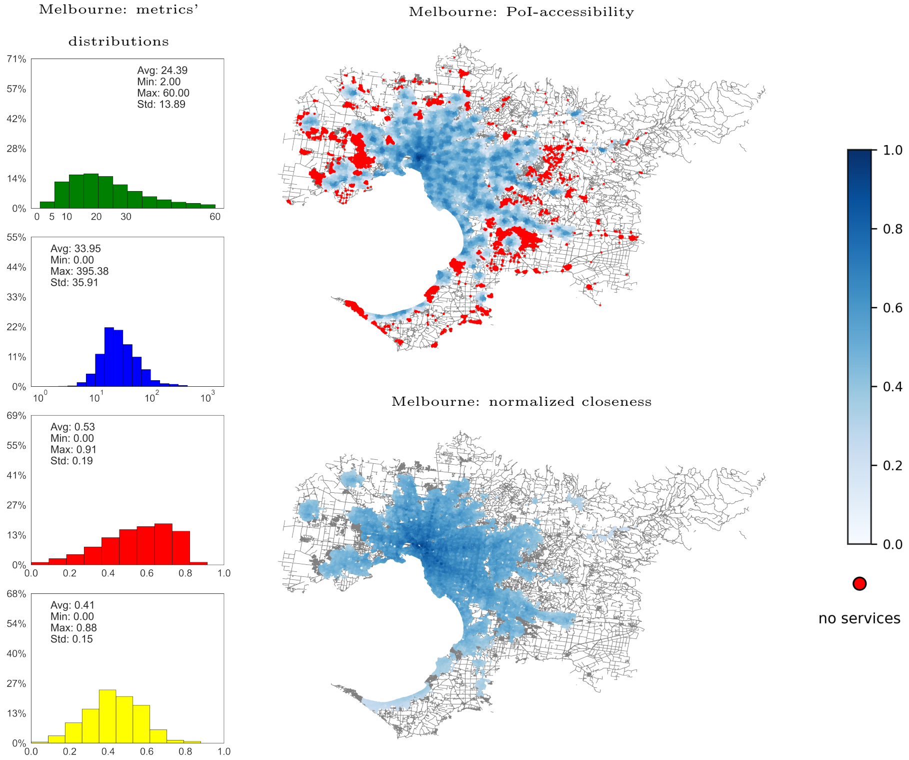

Figure 7: Melbourne: metrics distributions (left) and PoI-accessibility (top right) and normalized closeness (bottom right) heat maps. Base maps and data from OpenStreetMap and OpenStreetMap Foundation.

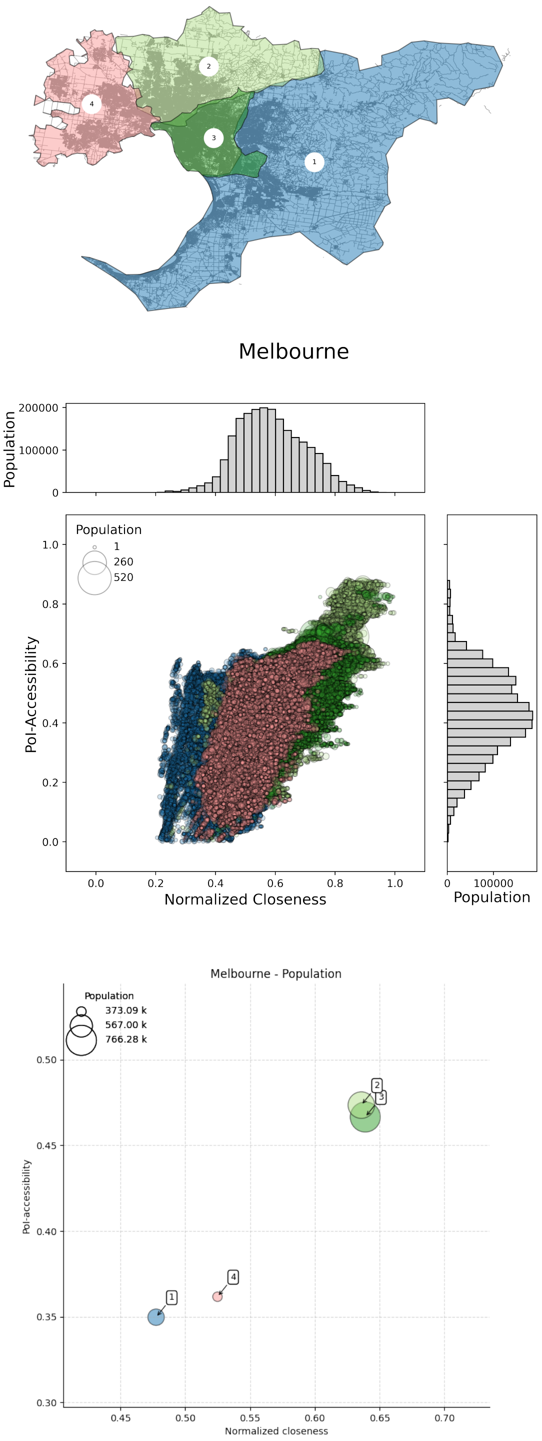

Figure 8: Melbourne: Infomap neighborhoods (top), PoI-accessibility vs normalized closeness aggregated by census areas (center), and neighborhoods (bottom). The maps contain information from OpenStreetMap and OpenStreetMap Foundation, which is made available under the Open Database License.

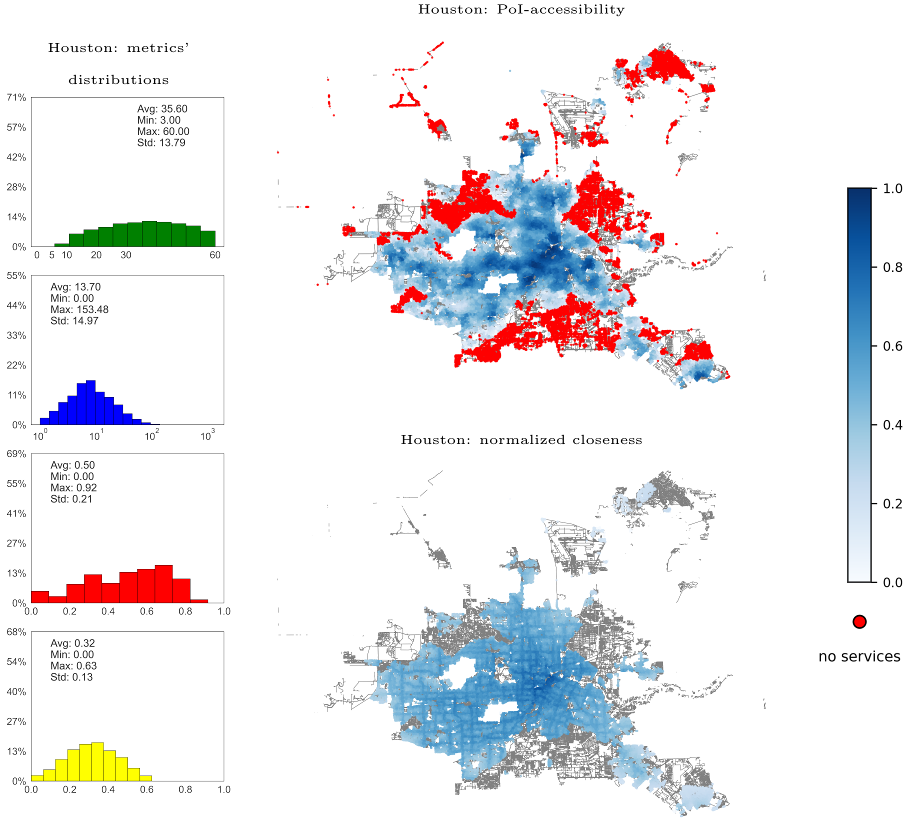

Figure 9: Houston: metrics distributions (left) and PoI-accessibility (top right) and normalized closeness (bottom right) heat maps. Base maps and data from OpenStreetMap and OpenStreetMap Foundation.

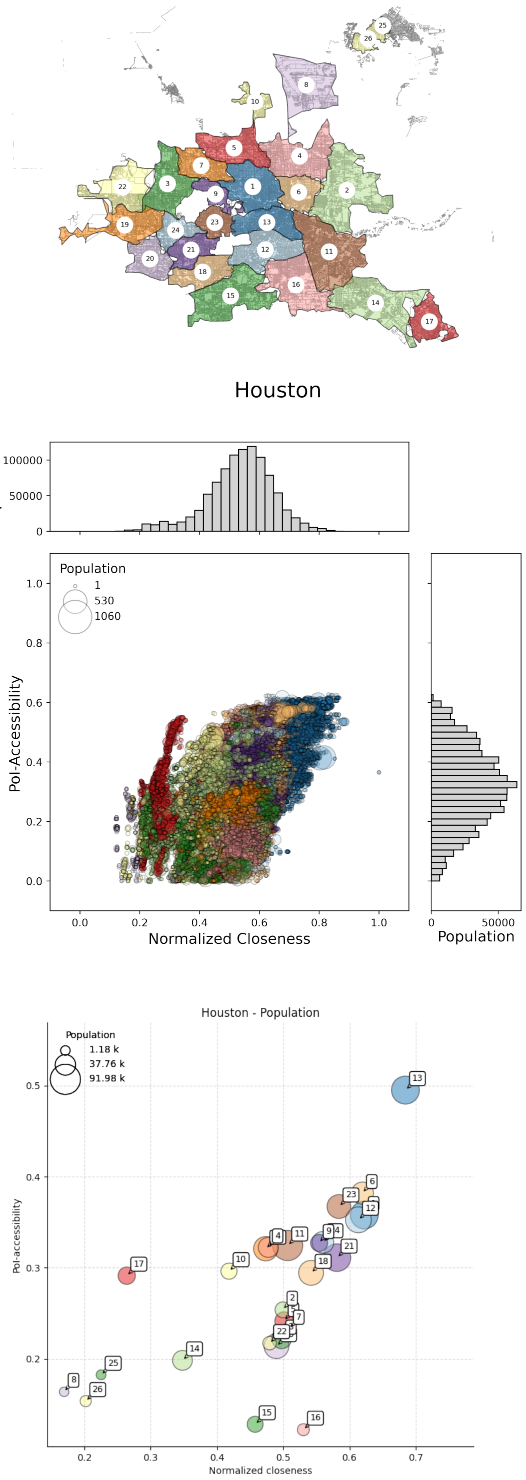

Figure 10: Houston: Infomap neighborhoods (top), PoI-accessibility vs normalized closeness aggregated by census areas (center), and neighborhoods (bottom). The maps contain information from OpenStreetMap and OpenStreetMap Foundation, which is made available under the Open Database License.
