# Supplementary material for: Understanding the interplay between urban segregation and accessibility to services with network analysis
Source: PLoS One. 2026 Apr 1;21(4):e0342156. doi: 10.1371/journal.pone.0342156 (PMC13042880; doi:10.1371/journal.pone.0342156)
Supplement: S6 Appendix — (PDF) [file pone.0342156.s006.pdf]

Understanding the interplay between urban segregation and accessibility to services with network analysis: Supplementary Material

More details on Italian cities

In this appendix we add some details on the analyses performed on the 10 biggest Italian cities, also showing at greater resolutions some plots already shown in the main paper.

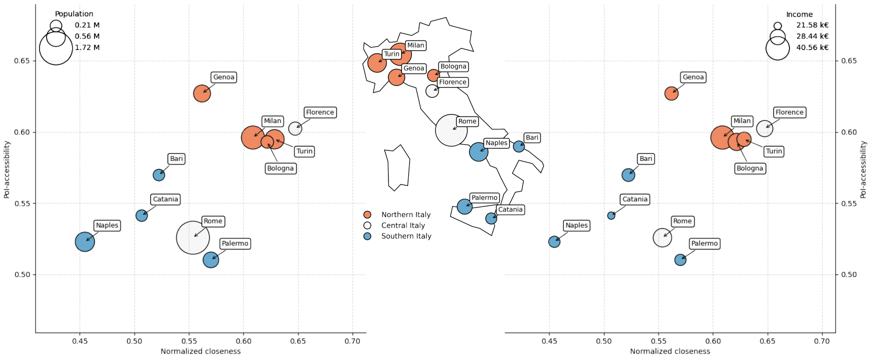

Figure 1: PoI-accessibility vs normalized closeness for the 10 biggest Italian cities (including Catania). Markers in the bubble charts are color-coded according to three geographical macro-regions, and their sizes are proportional to population (left) or to average income (right).

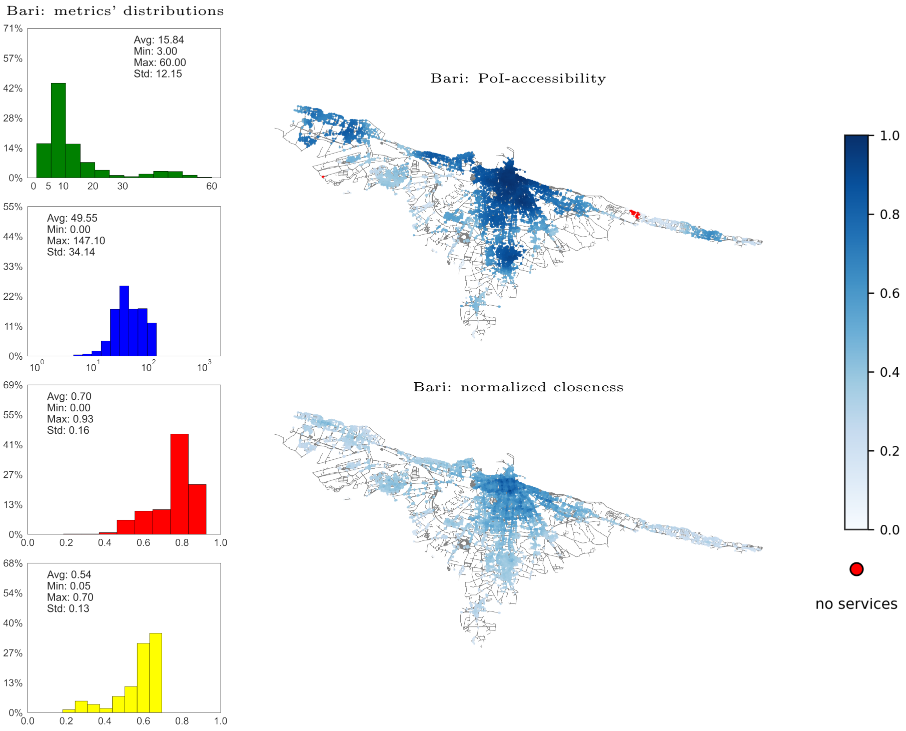

Figure 2: Bari: metrics distributions (left) and PoI-accessibility (top right) and normalized closeness (bottom right) heat maps. Base maps and data from OpenStreetMap and OpenStreetMap Foundation.

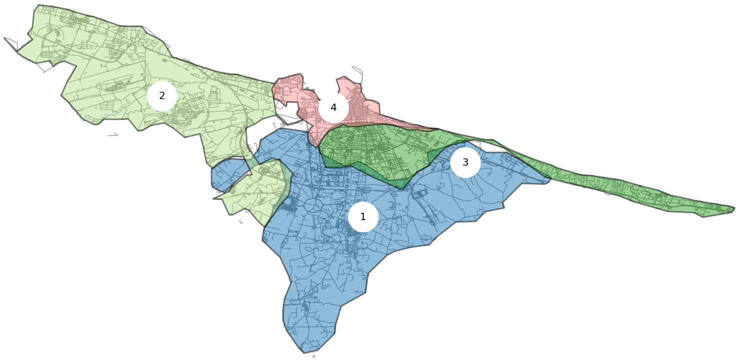

Bari

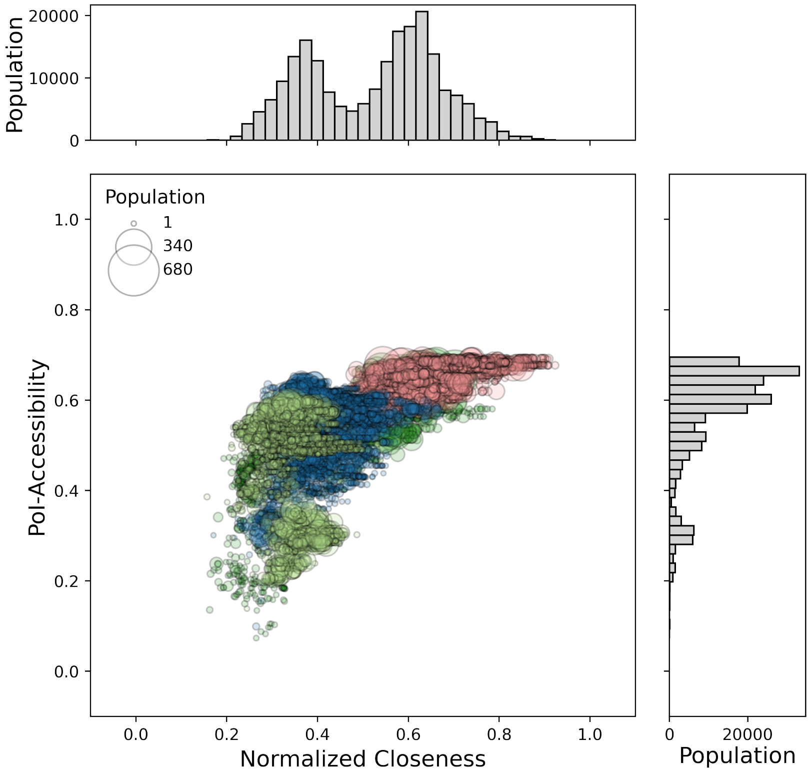

Figure 3: Bari: Infomap neighborhoods (top), PoI-accessibility vs normalized closeness aggregated by census areas (bottom). The map contains information from OpenStreetMap and OpenStreetMap Foundation, which is made available under the Open Database License.

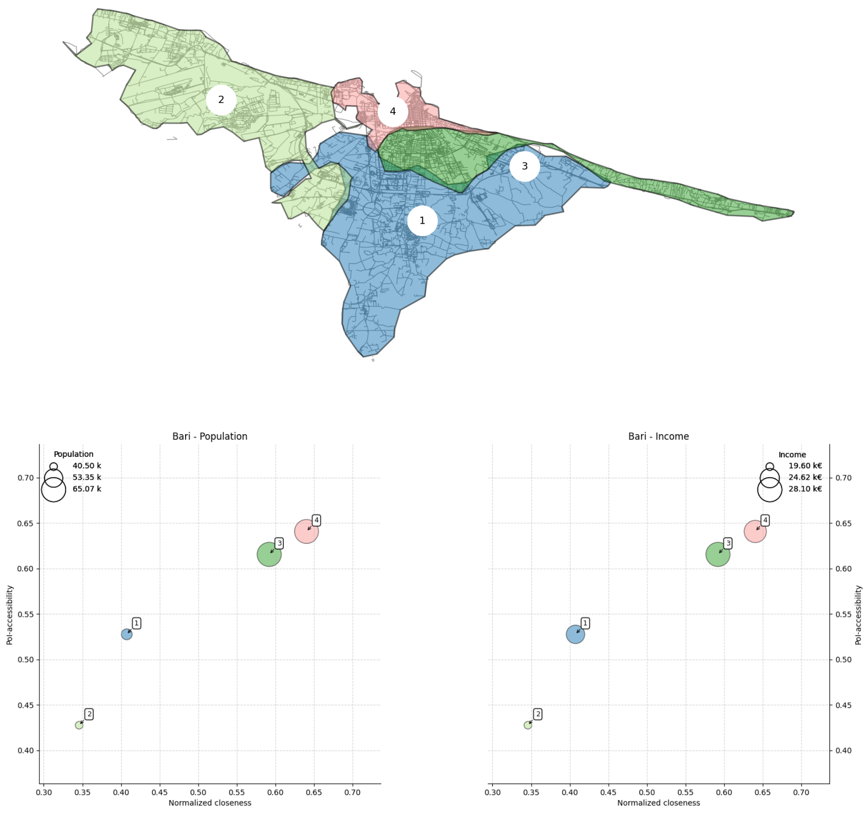

Figure 4: Bari: Infomap neighborhoods (top), PoI-accessibility vs normalized closeness aggregated neighborhoods (center and bottom). The map contains information from OpenStreetMap and OpenStreetMap Foundation, which is made available under the Open Database License.

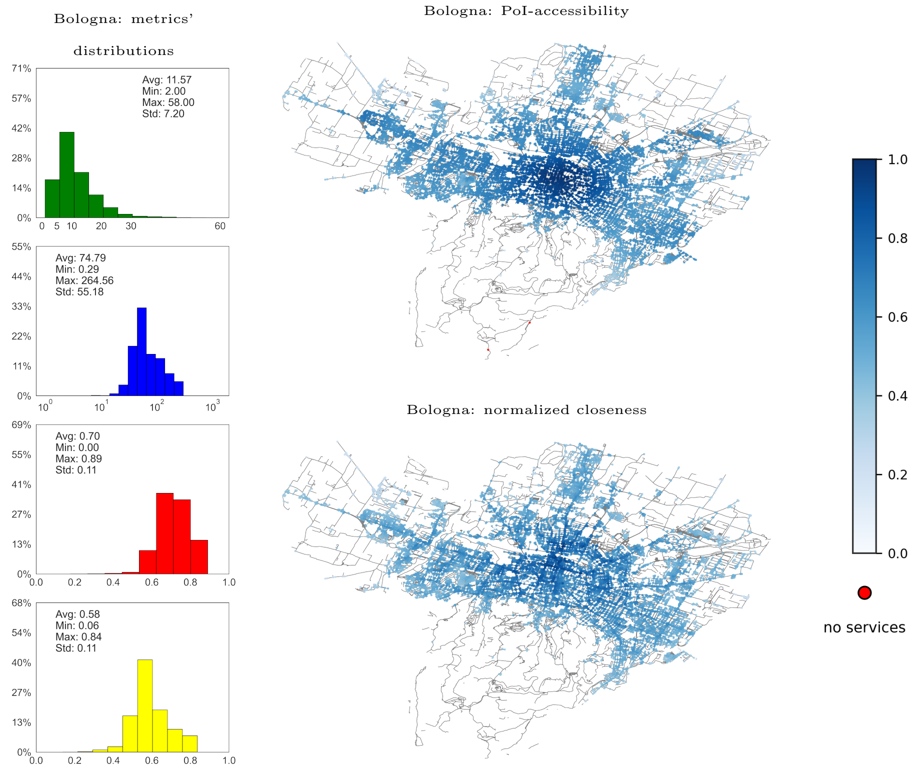

Figure 5: Bologna: metrics distributions (left) and PoI-accessibility (top right) and normalized closeness (bottom right) heat maps. Base maps and data from OpenStreetMap and OpenStreetMap Foundation.

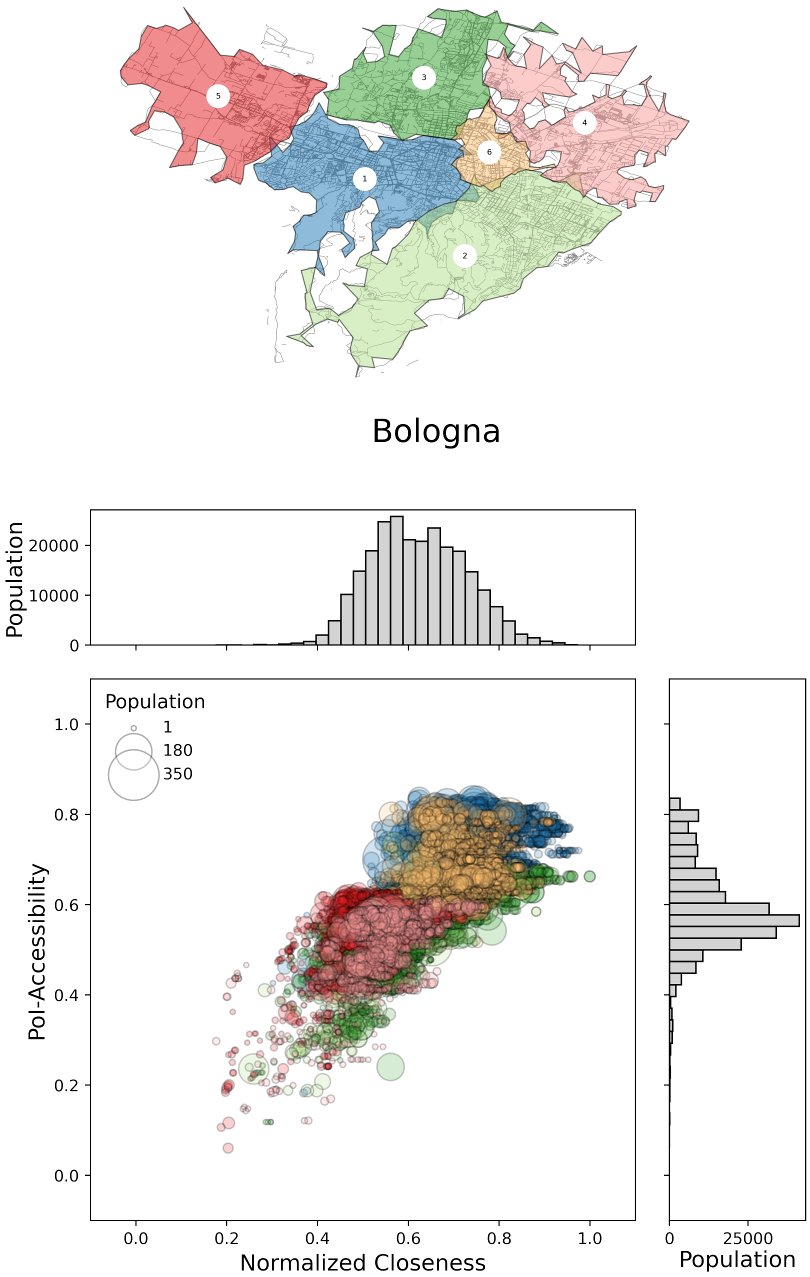

Figure 6: Bologna: Infomap neighborhoods (top), PoI-accessibility vs normalized closeness aggregated by census areas (bottom). The map contains information from OpenStreetMap and OpenStreetMap Foundation, which is made available under the Open Database License.

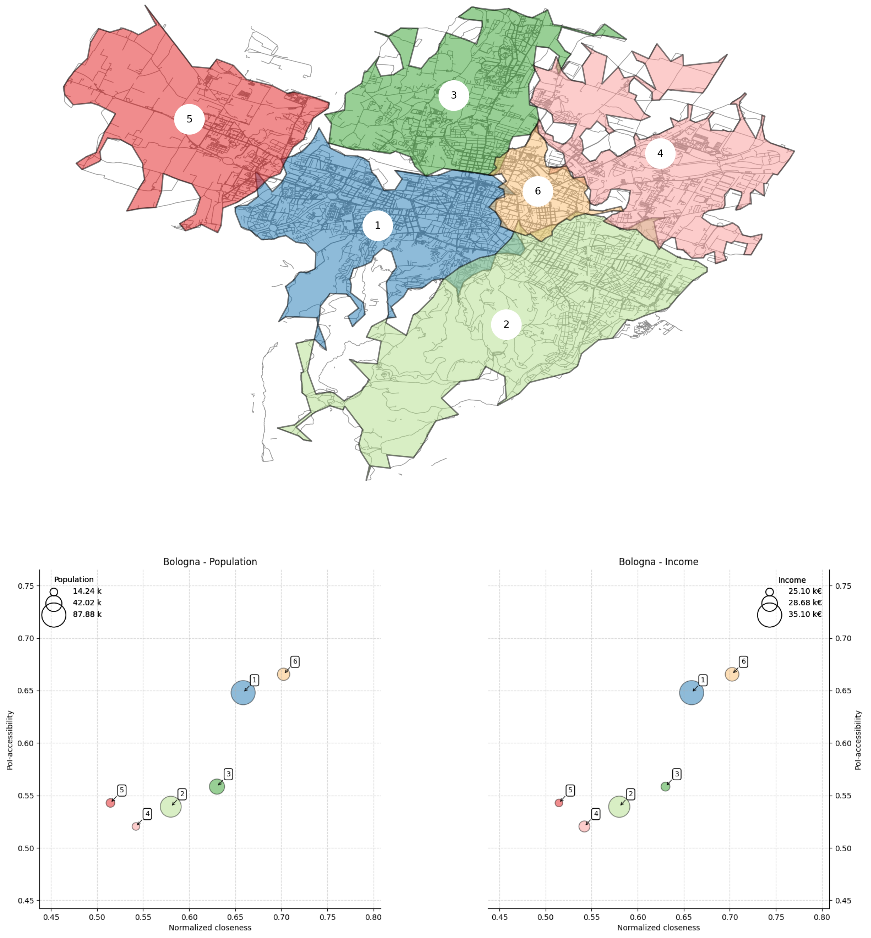

Figure 7: Bologna: Infomap neighborhoods (top), PoI-accessibility vs normalized closeness aggregated by neighborhoods (bottom). The map contains information from OpenStreetMap and OpenStreetMap Foundation, which is made available under the Open Database License.

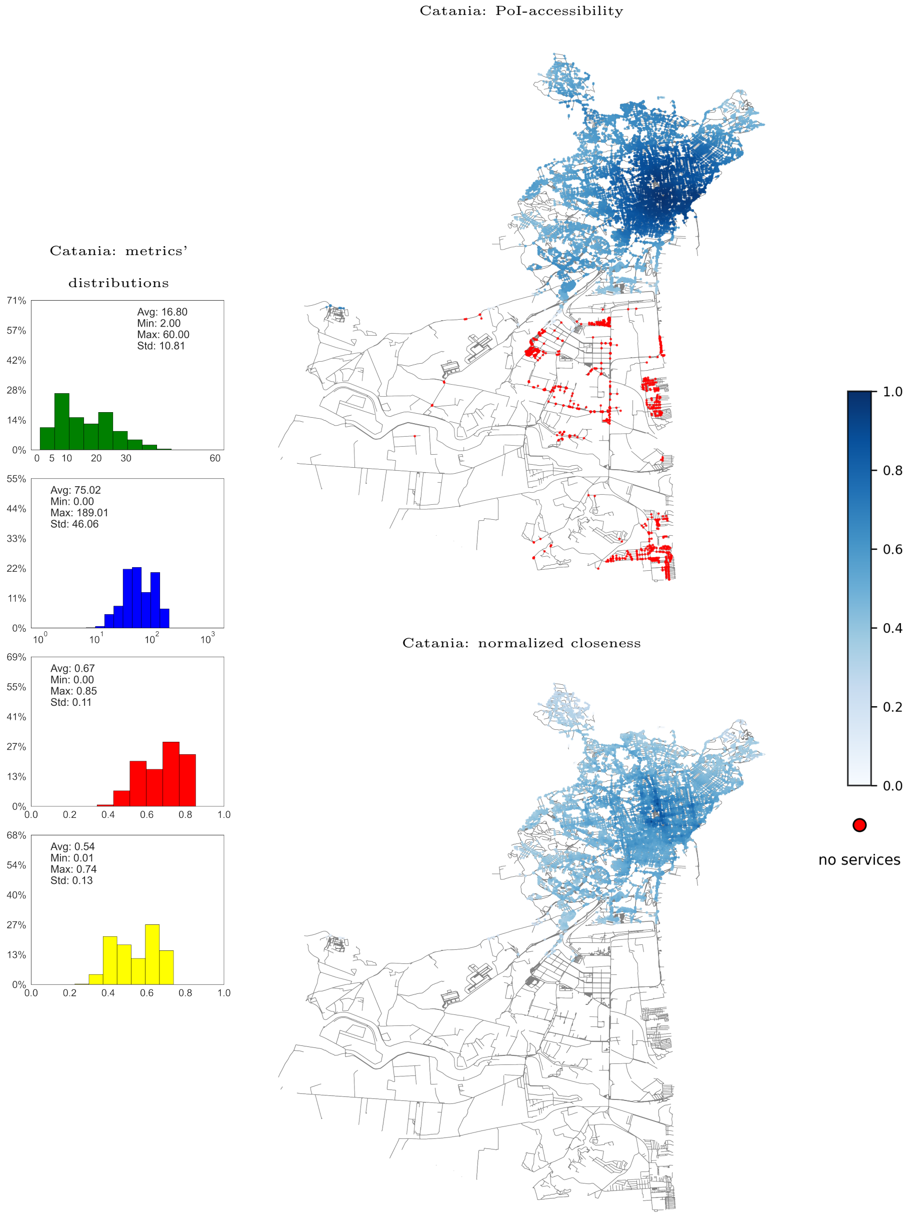

Figure 8: Catania: metrics distributions (left) and PoI-accessibility (top right) and normalized closeness (bottom right) heat maps. Base maps and data from OpenStreetMap and OpenStreetMap Foundation.

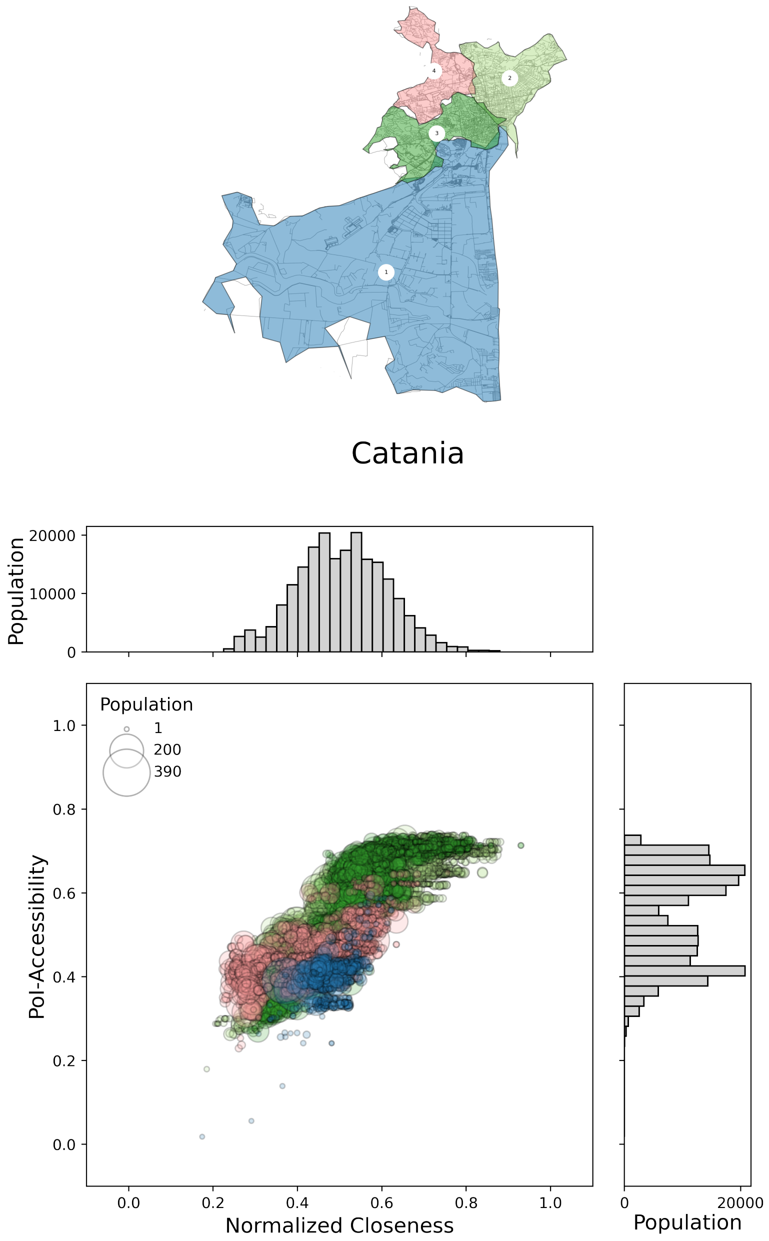

Figure 9: Catania: Infomap neighborhoods (top), PoI-accessibility vs normalized closeness aggregated by census areas (bottom). The map contains information from OpenStreetMap and OpenStreetMap Foundation, which is made available under the Open Database License.

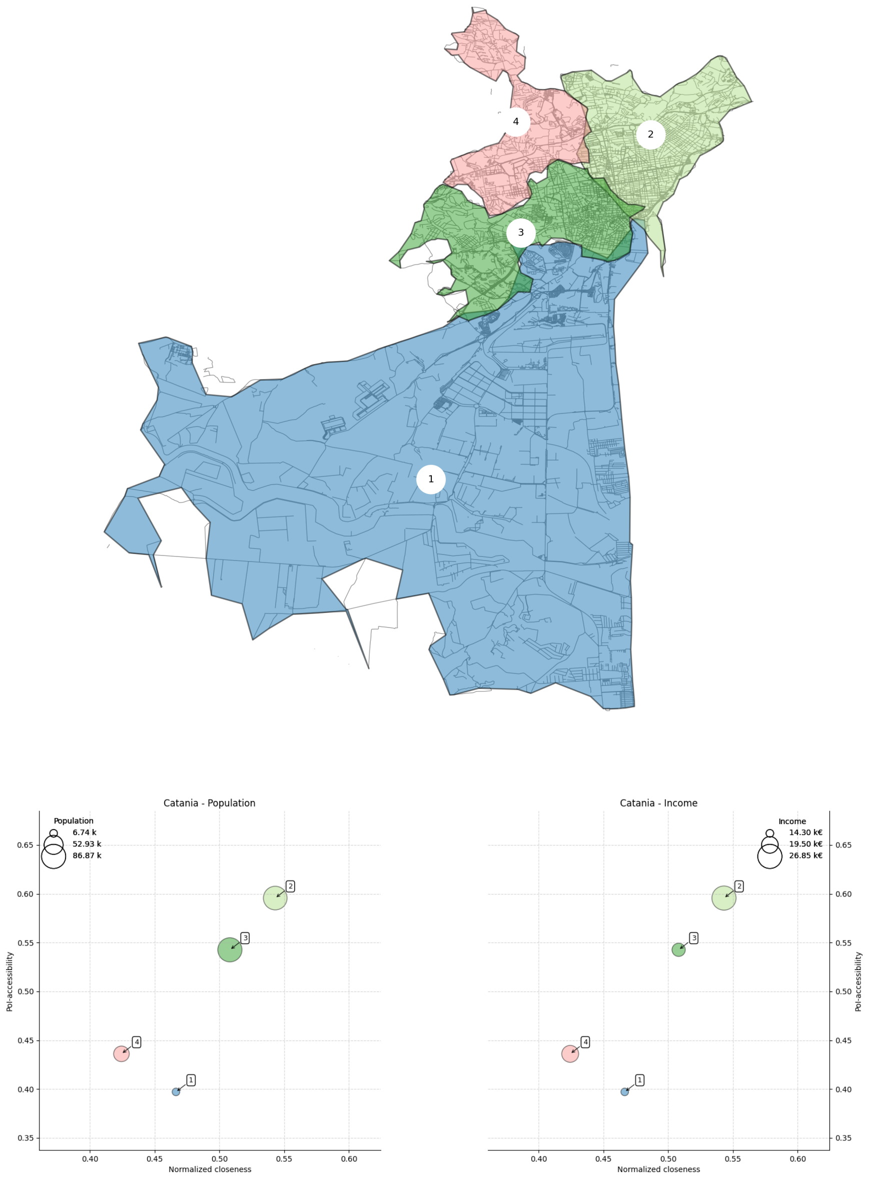

Figure 10: Catania: Infomap neighborhoods (top), PoI-accessibility vs normalized closeness aggregated by neighborhoods (bottom). The map contains information from OpenStreetMap and OpenStreetMap Foundation, which is made available under the Open Database License.

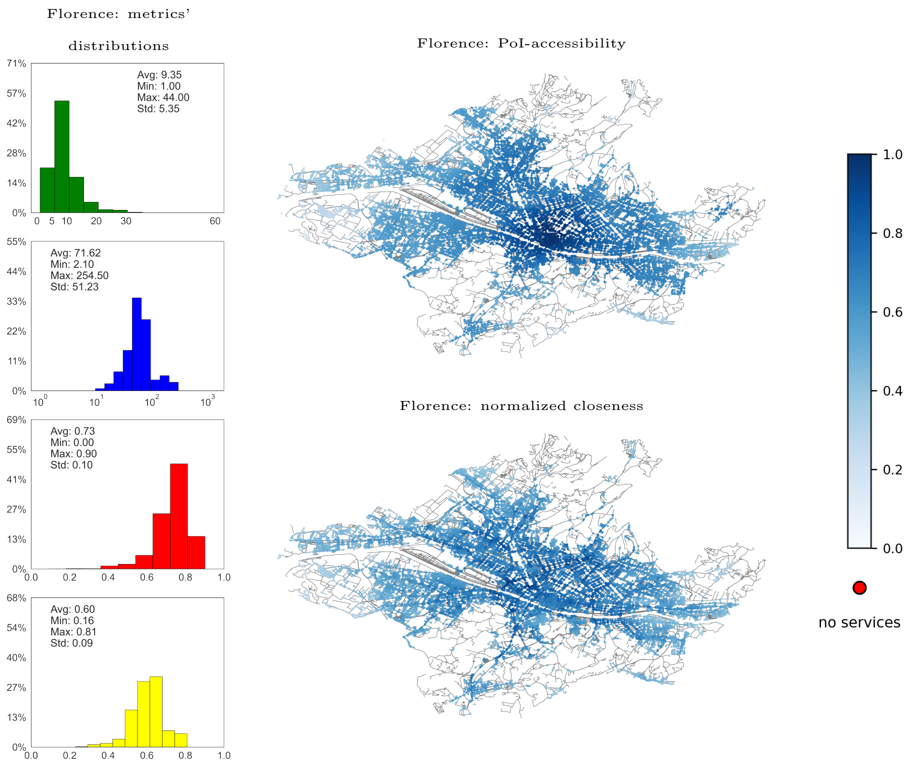

Figure 11: Florence: metrics distributions (left) and PoI-accessibility (top right) and normalized closeness (bottom right) heat maps. Base maps and data from OpenStreetMap and OpenStreetMap Foundation.

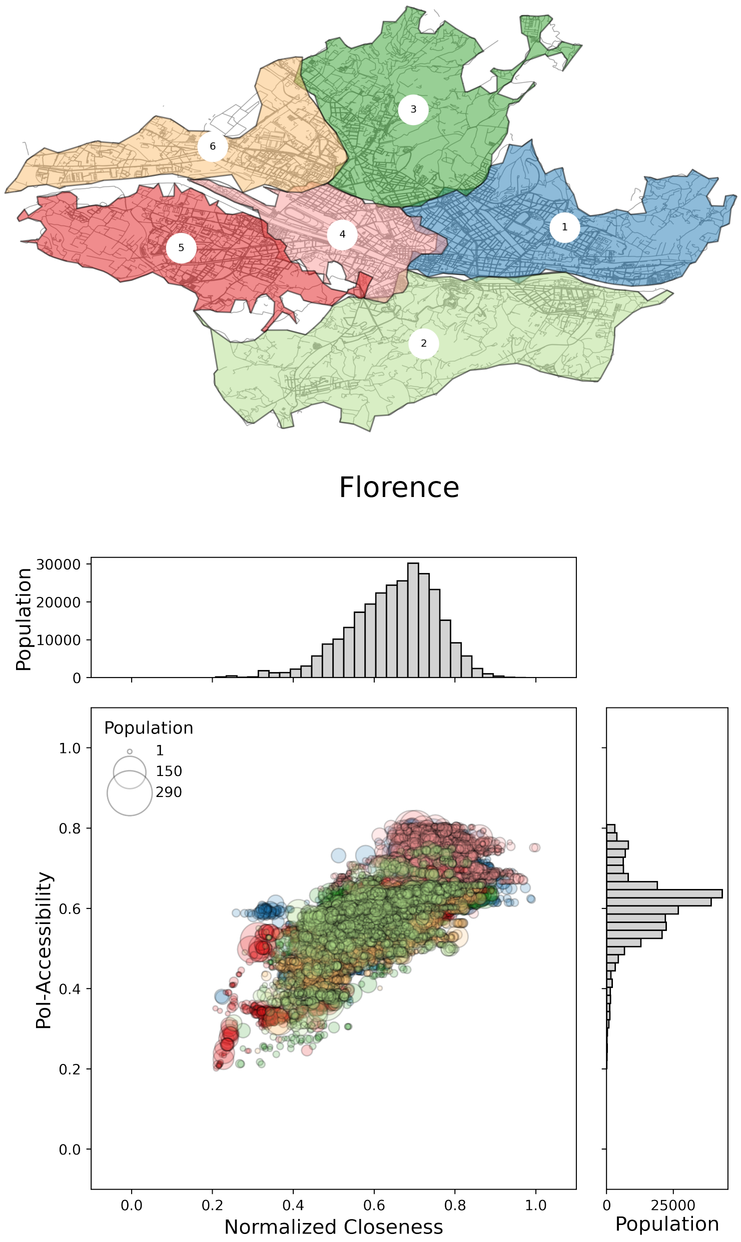

Figure 12: Florence: Infomap neighborhoods (top), PoI-accessibility vs normalized closeness aggregated by census areas (bottom). The map contains information from OpenStreetMap and OpenStreetMap Foundation, which is made available under the Open Database License.

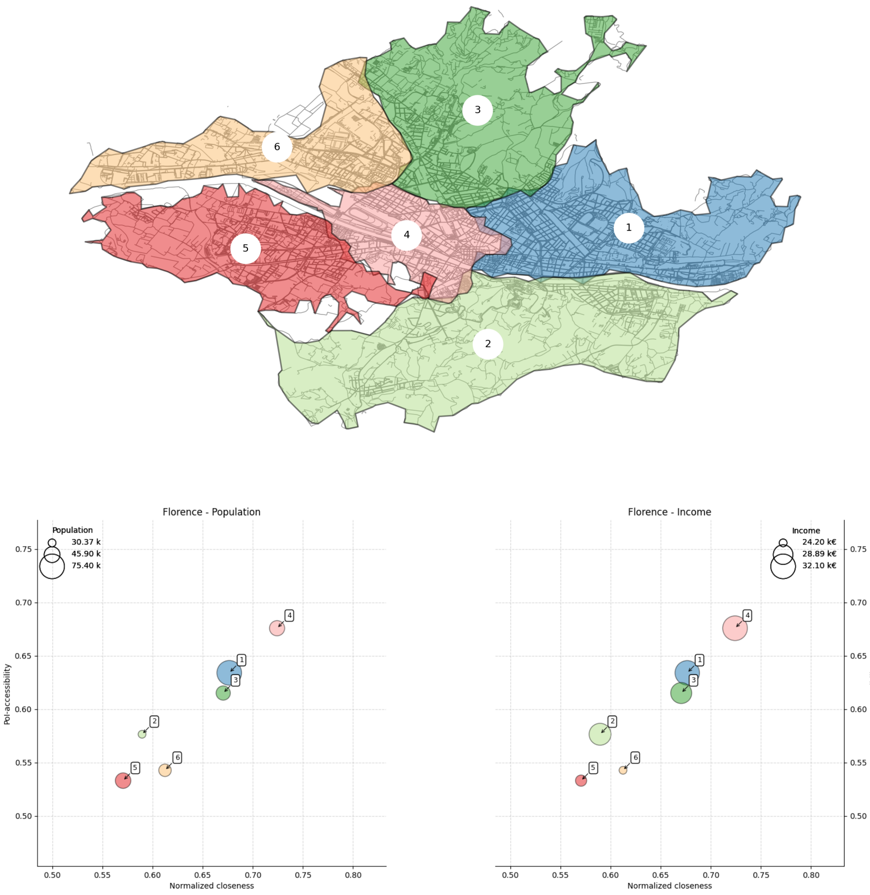

Figure 13: Florence: Infomap neighborhoods (top), PoI-accessibility vs normalized closeness aggregated by neighborhoods (bottom). The map contains information from OpenStreetMap and OpenStreetMap Foundation, which is made available under the Open Database License.

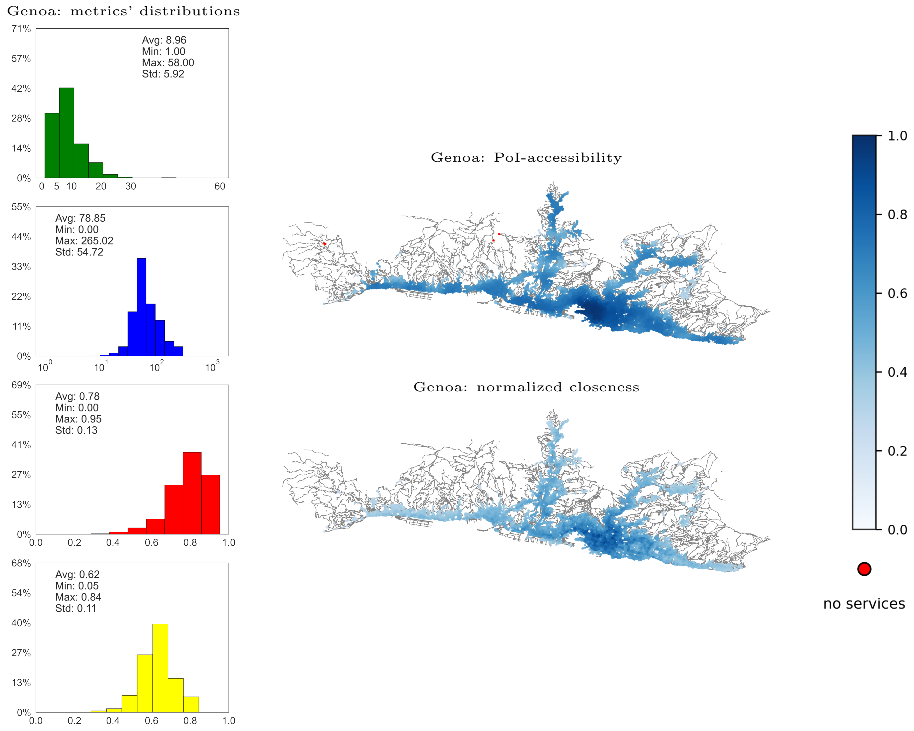

Figure 14: Genoa: metrics distributions (left) and PoI-accessibility (top right) and normalized closeness (bottom right) heat maps. Base maps and data from OpenStreetMap and OpenStreetMap Foundation.

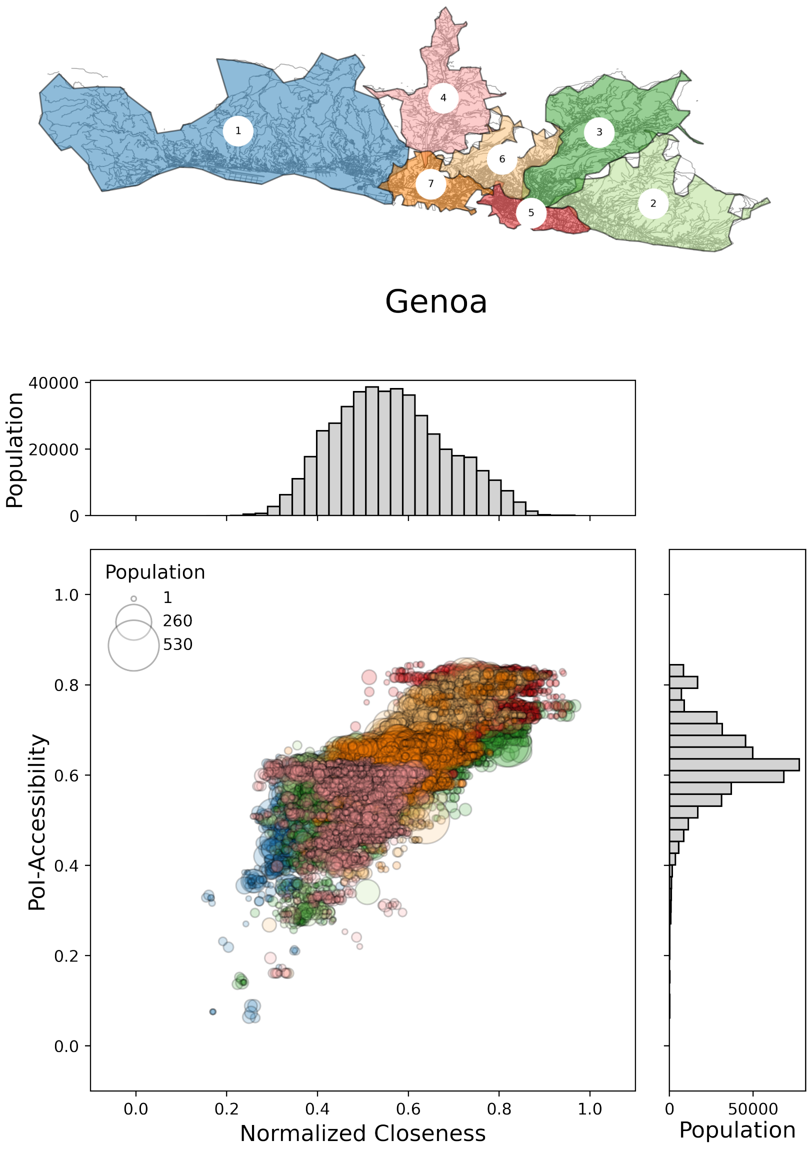

Figure 15: Genoa: Infomap neighborhoods (top), PoI-accessibility vs normalized closeness aggregated by census areas (bottom). The map contains information from OpenStreetMap and OpenStreetMap Foundation, which is made available under the Open Database License.

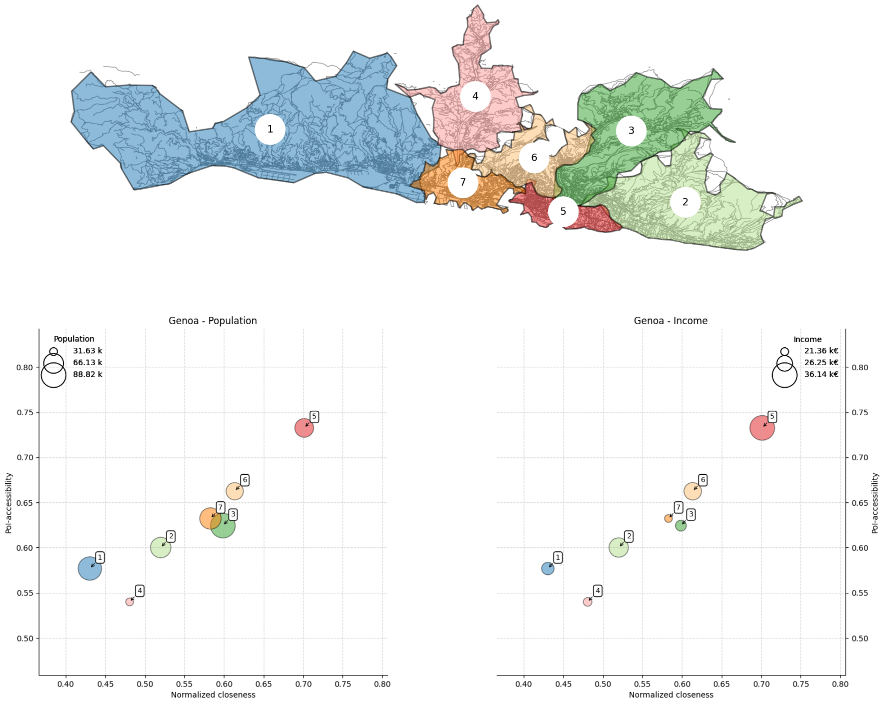

Figure 16: Genoa: Infomap neighborhoods (top), PoI-accessibility vs normalized closeness aggregated by neighborhoods (bottom). The map contains information from OpenStreetMap and OpenStreetMap Foundation, which is made available under the Open Database License.

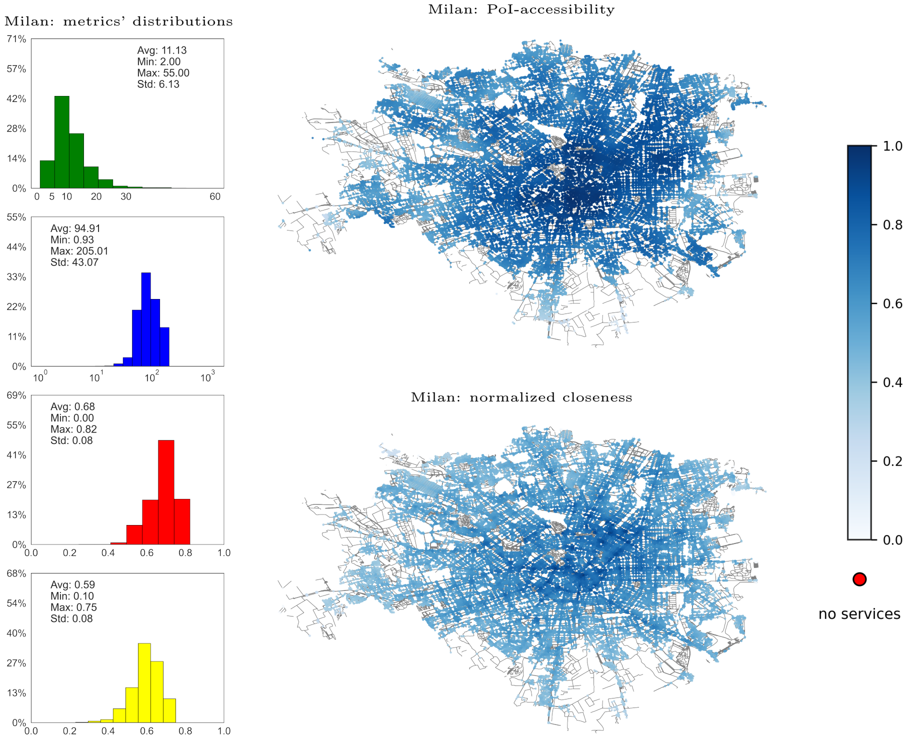

Figure 17: Milan: metrics distributions (left) and PoI-accessibility (top right) and normalized closeness (bottom right) heat maps. Base maps and data from OpenStreetMap and OpenStreetMap Foundation.

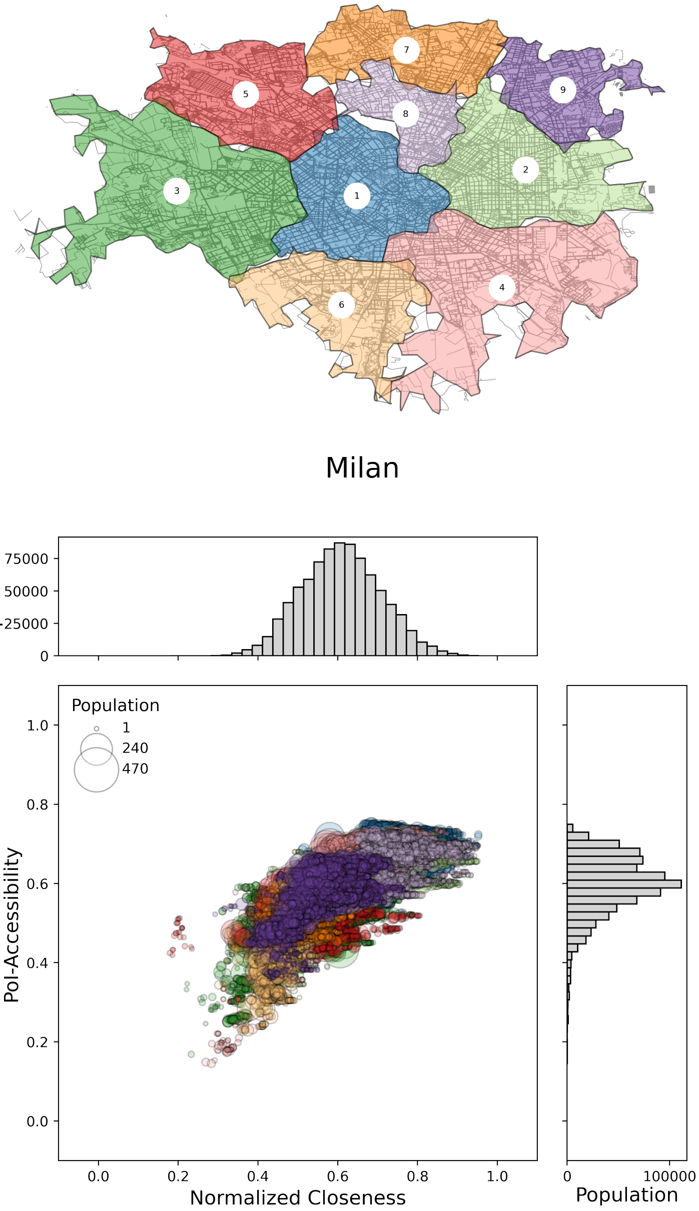

Figure 18: Milan: Infomap neighborhoods (top), PoI-accessibility vs normalized closeness aggregated by census areas (bottom). The map contains information from OpenStreetMap and OpenStreetMap Foundation, which is made available under the Open Database License.

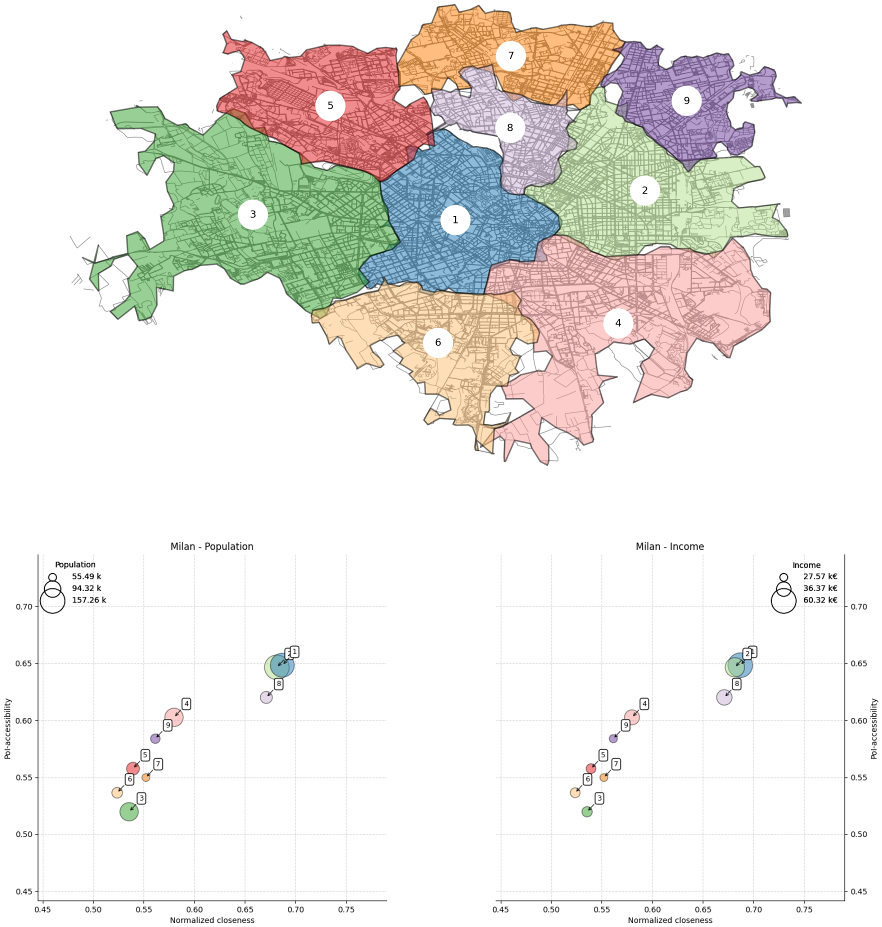

Figure 19: Milan: Infomap neighborhoods (top), PoI-accessibility vs normalized closeness aggregated by neighborhoods (bottom). The map contains information from OpenStreetMap and OpenStreetMap Foundation, which is made available under the Open Database License.

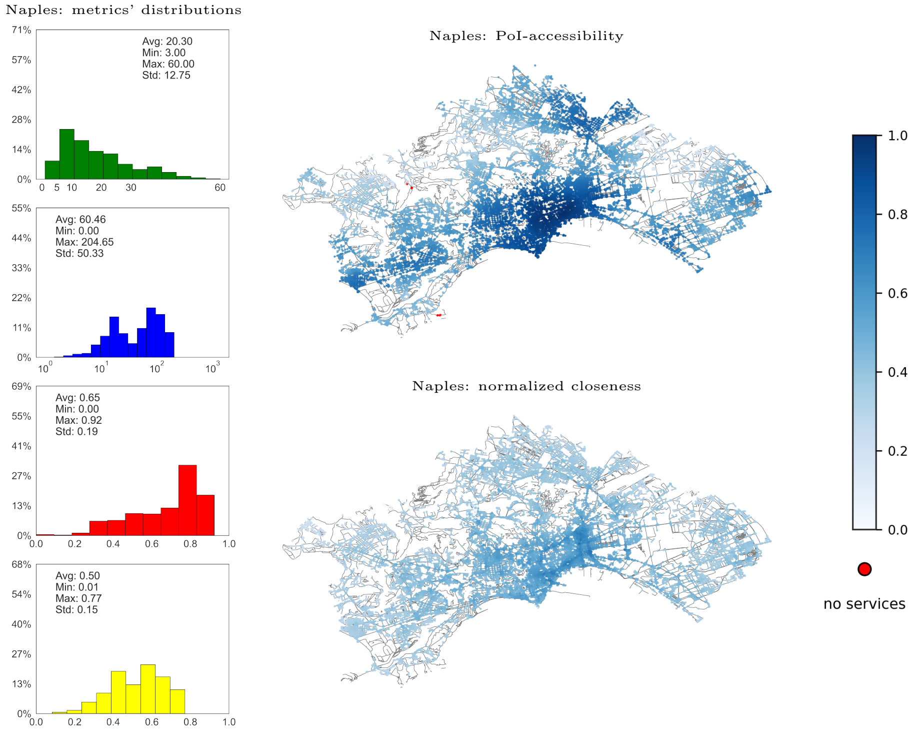

Figure 20: Naples: metrics distributions (left) and PoI-accessibility (top right) and normalized closeness (bottom right) heat maps. Base maps and data from OpenStreetMap and OpenStreetMap Foundation.

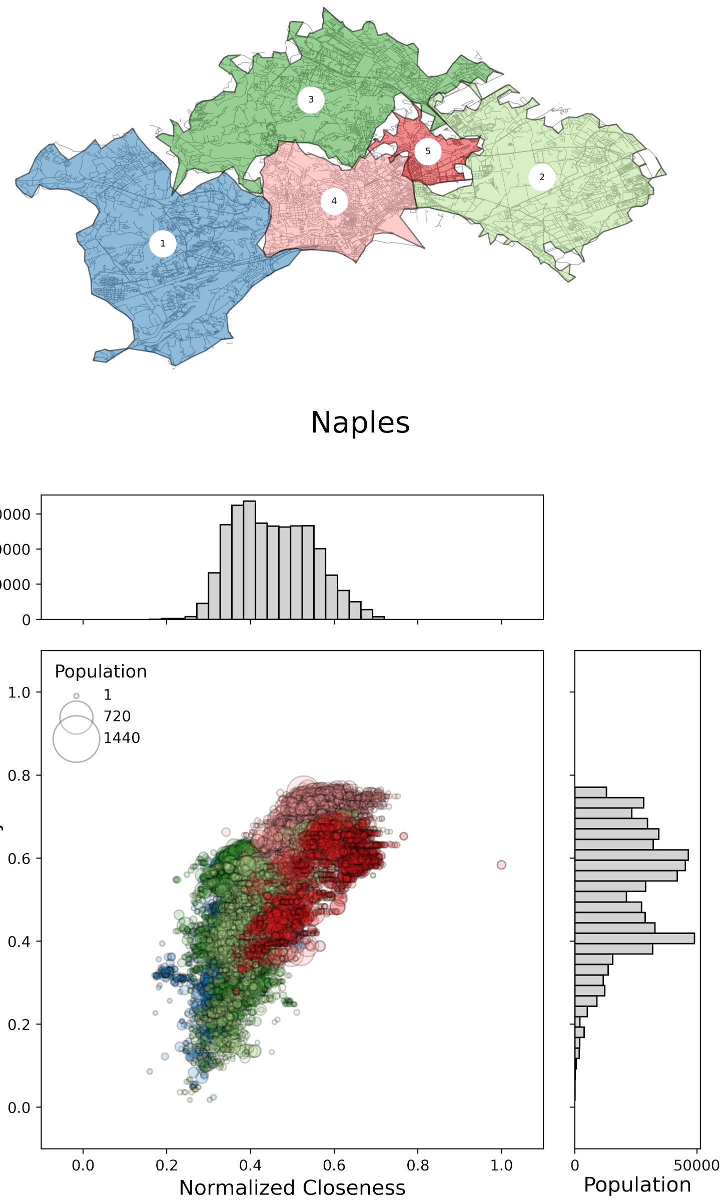

Figure 21: Naples: Infomap neighborhoods (top), PoI-accessibility vs normalized closeness aggregated by census areas (bottom). The map contains information from OpenStreetMap and OpenStreetMap Foundation, which is made available under the Open Database License.

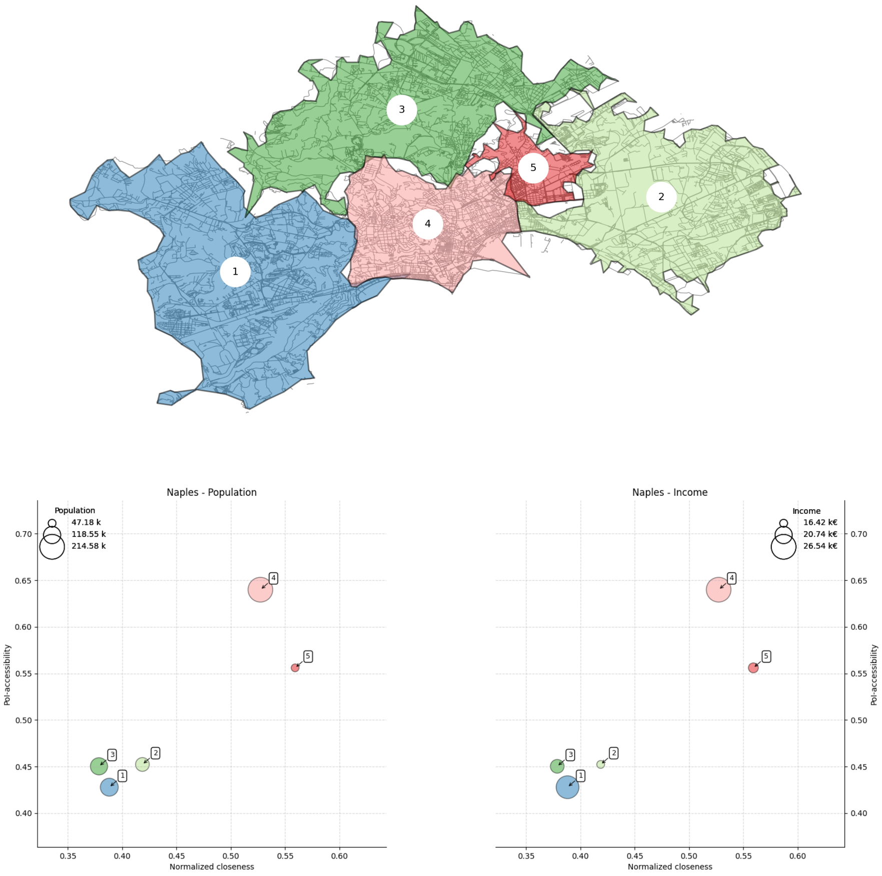

Figure 22: Naples: Infomap neighborhoods (top), PoI-accessibility vs normalized closeness aggregated by neighborhoods (bottom). The map contains information from OpenStreetMap and OpenStreetMap Foundation, which is made available under the Open Database License.

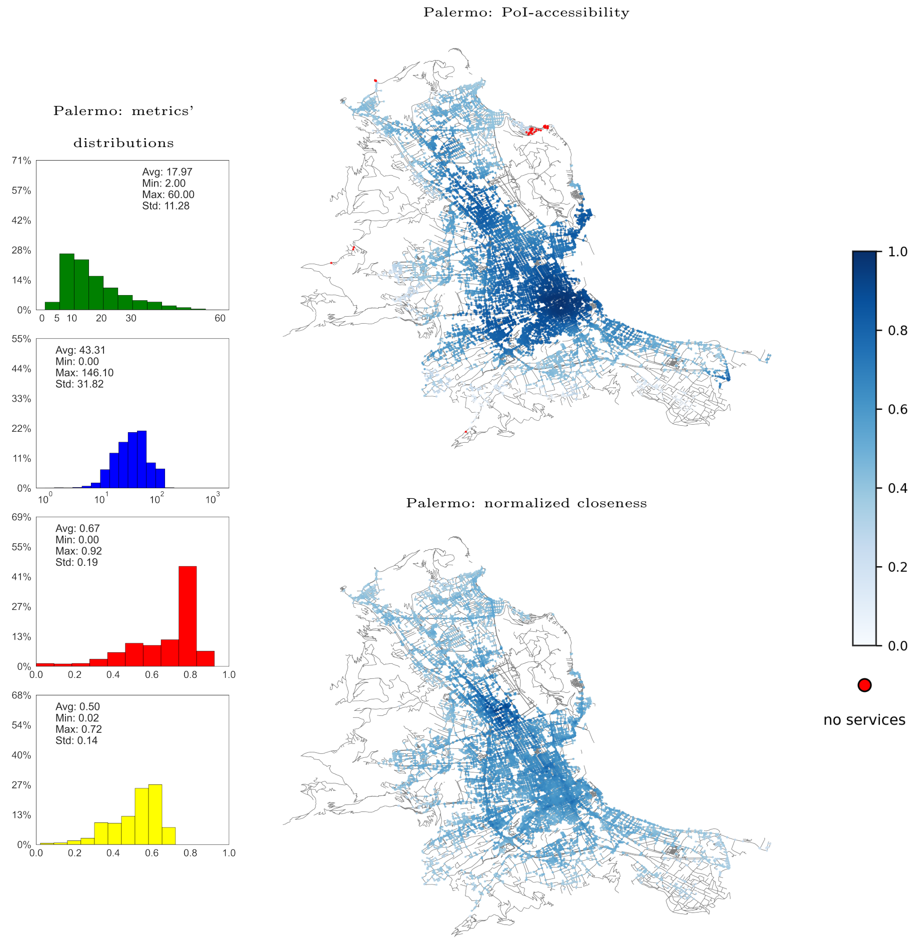

Figure 23: Palermo: metrics distributions (left) and PoI-accessibility (top right) and normalized closeness (bottom right) heat maps. Base maps and data from OpenStreetMap and OpenStreetMap Foundation.

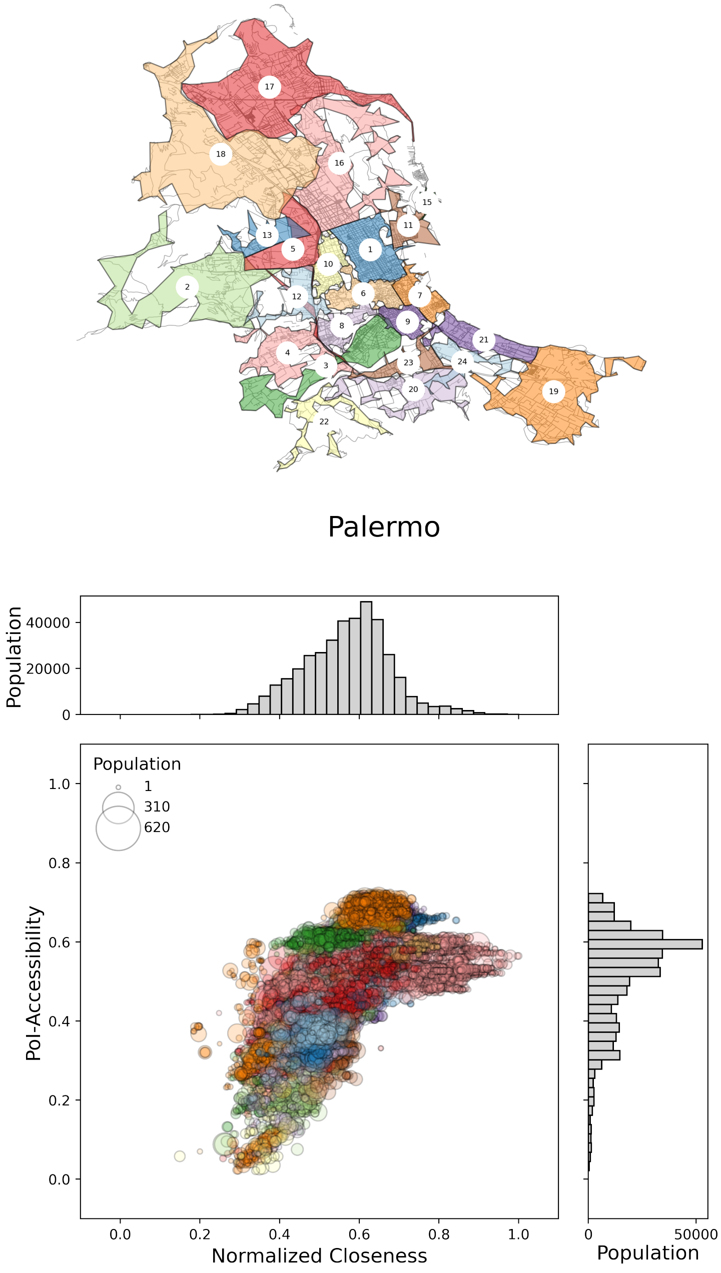

Figure 24: Palermo: Infomap neighborhoods (top), PoI-accessibility vs normalized closeness aggregated by census areas (bottom). The map contains information from OpenStreetMap and OpenStreetMap Foundation, which is made available under the Open Database License.

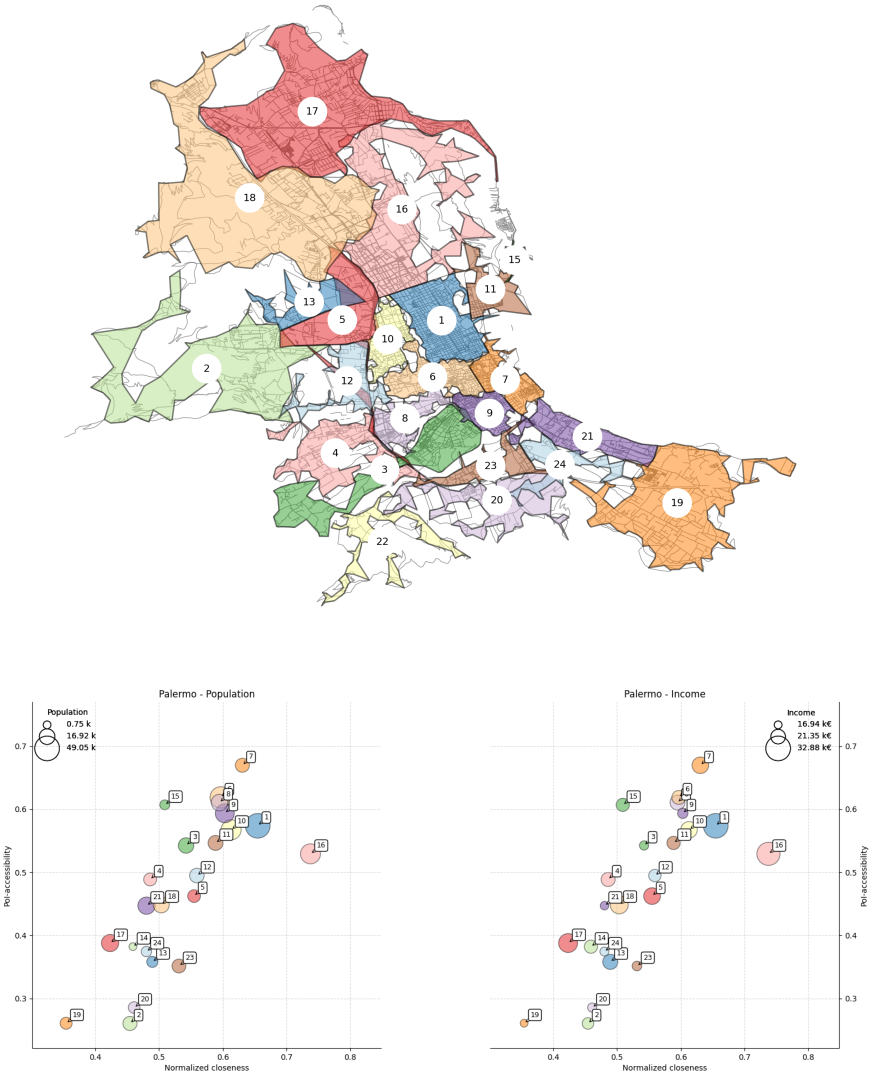

Figure 25: Palermo: Infomap neighborhoods (top), PoI-accessibility vs normalized closeness aggregated by neighborhoods (bottom). The map contains information from OpenStreetMap and OpenStreetMap Foundation, which is made available under the Open Database License.

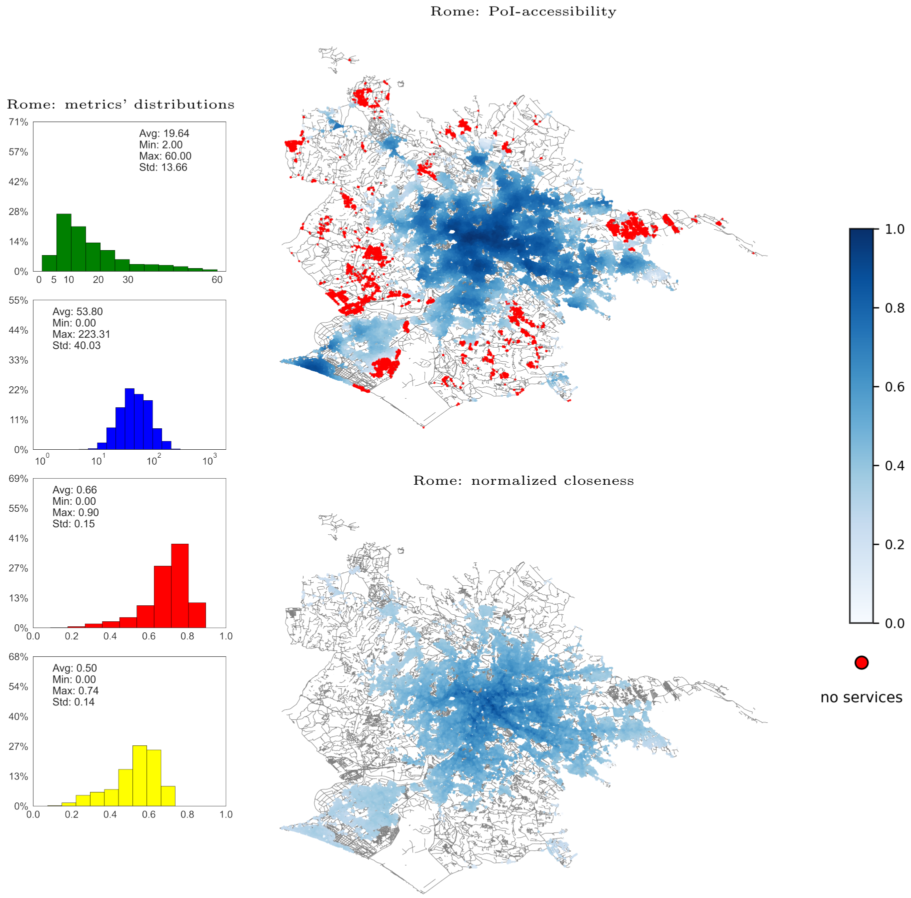

Figure 26: Rome: metrics distributions (left) and PoI-accessibility (top right) and normalized closeness (bottom right) heat maps. Base maps and data from OpenStreetMap and OpenStreetMap Foundation.

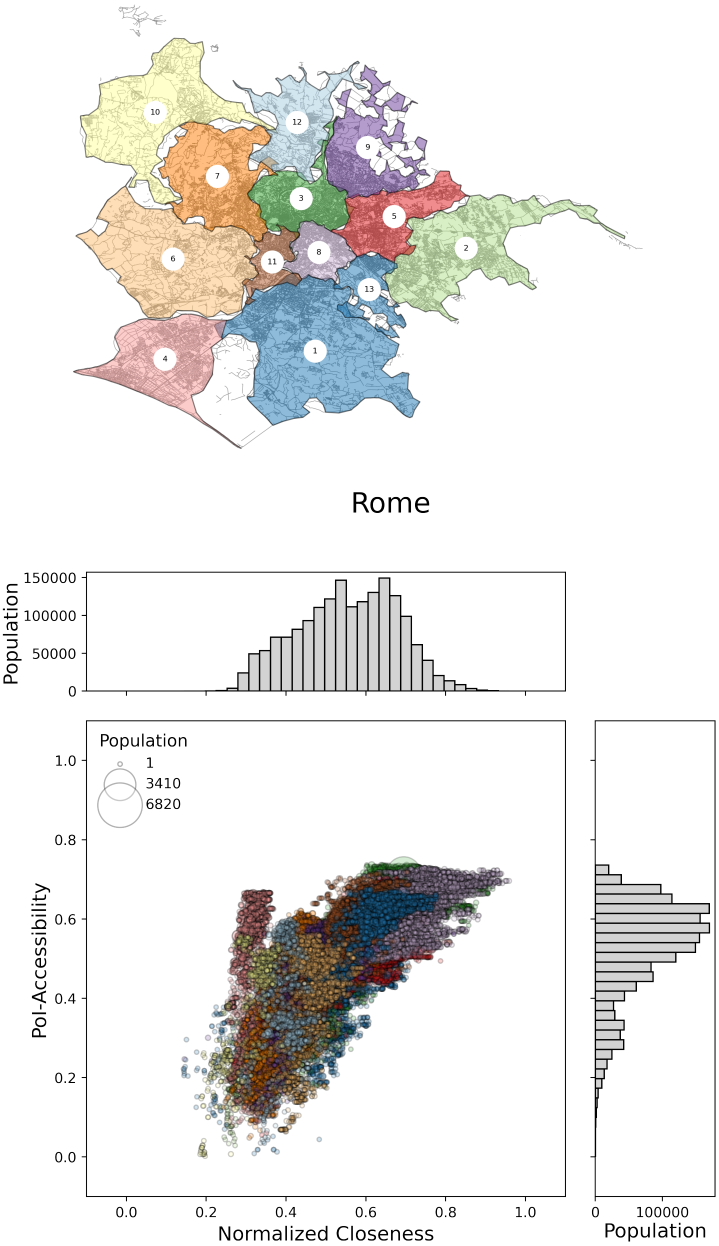

Figure 27: Rome: Infomap neighborhoods (top), PoI-accessibility vs normalized closeness aggregated by census areas (center) (bottom). The map contains information from OpenStreetMap and OpenStreetMap Foundation, which is made available under the Open Database License.

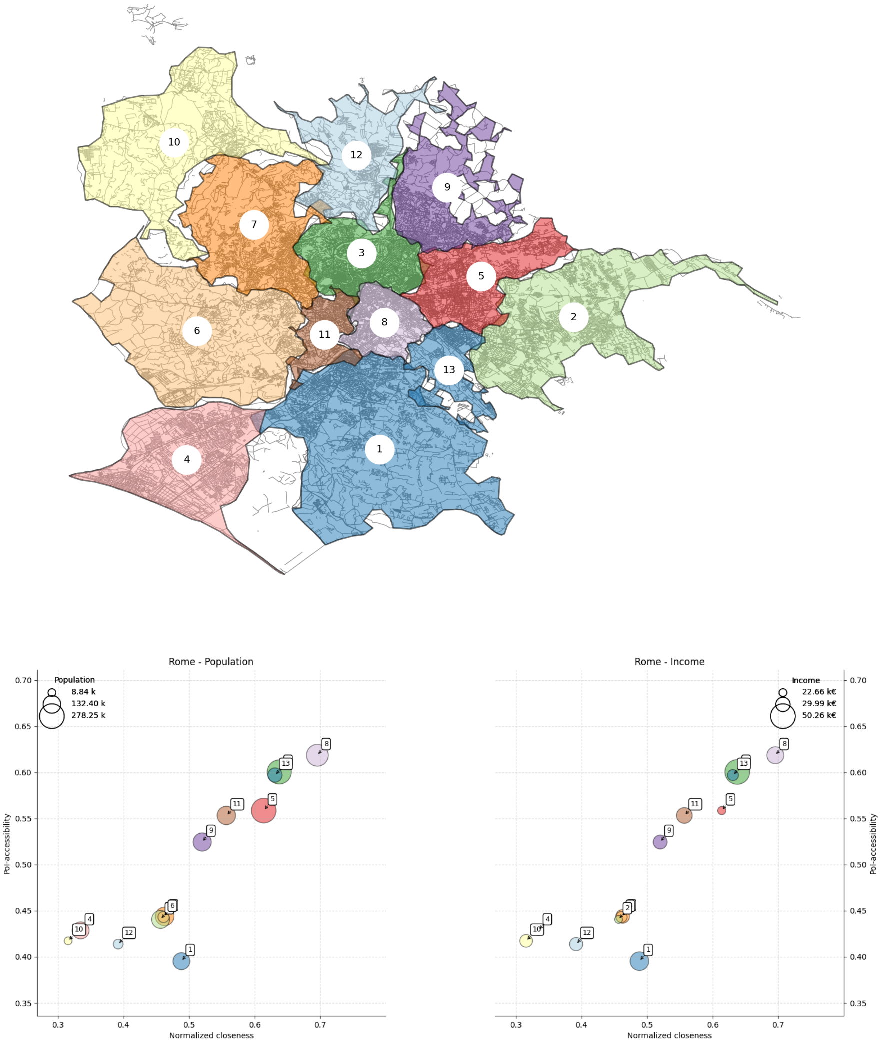

Figure 28: Rome: Infomap neighborhoods (top), PoI-accessibility vs normalized closeness aggregated by neighborhoods (bottom). The map contains information from OpenStreetMap and OpenStreetMap Foundation, which is made available under the Open Database License.

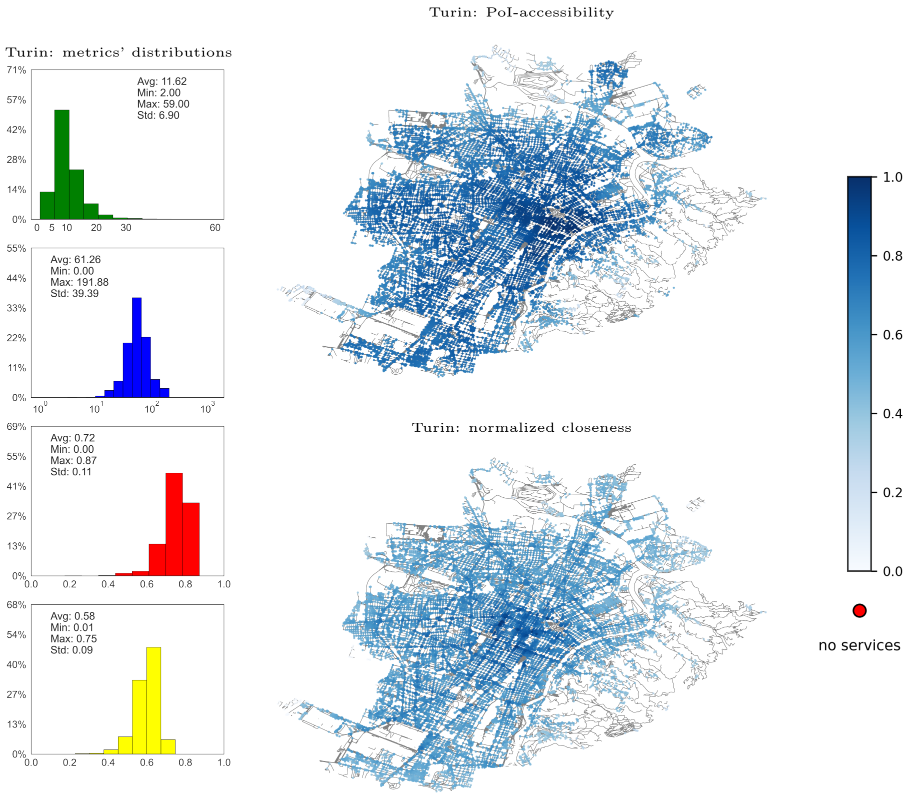

Figure 29: Turin: metrics distributions (left) and PoI-accessibility (top right) and normalized closeness (bottom right) heat maps. Base maps and data from OpenStreetMap and OpenStreetMap Foundation.

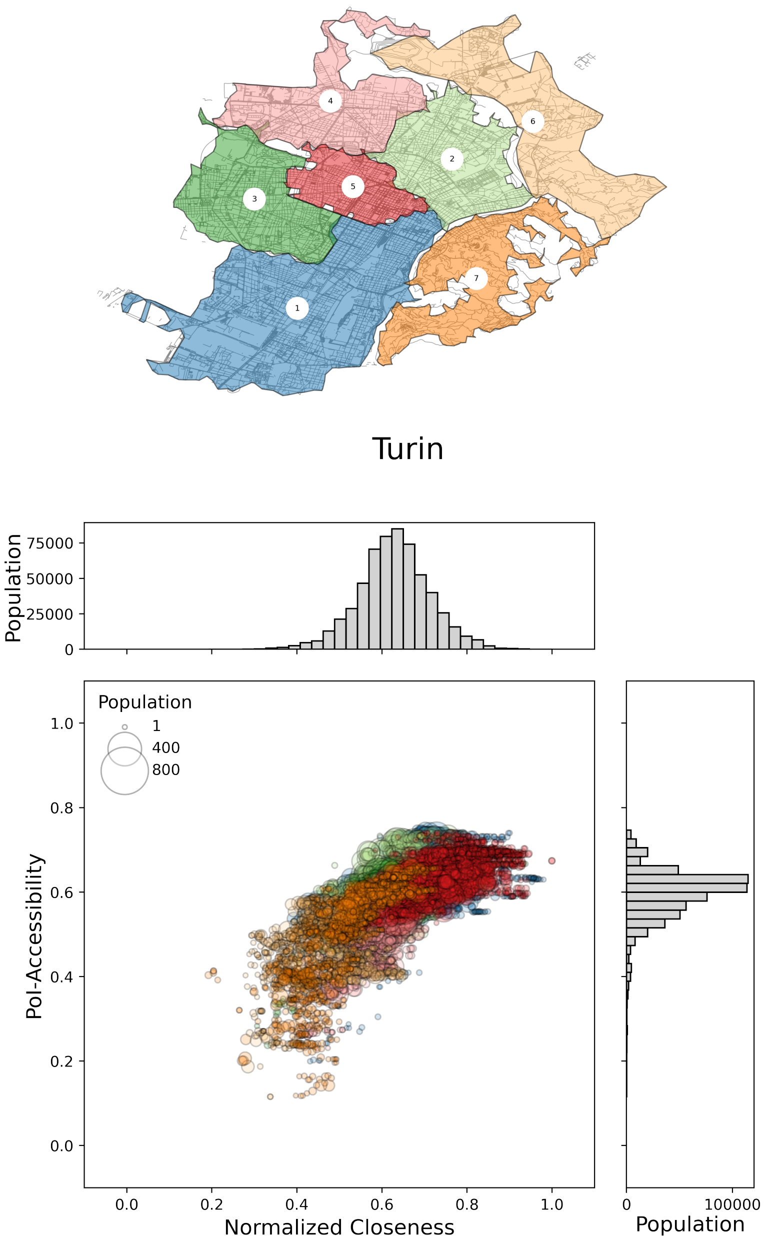

Figure 30: Turin: Infomap neighborhoods (top), PoI-accessibility vs normalized closeness aggregated by census areas (bottom). The map contains information from OpenStreetMap and OpenStreetMap Foundation, which is made available under the Open Database License.

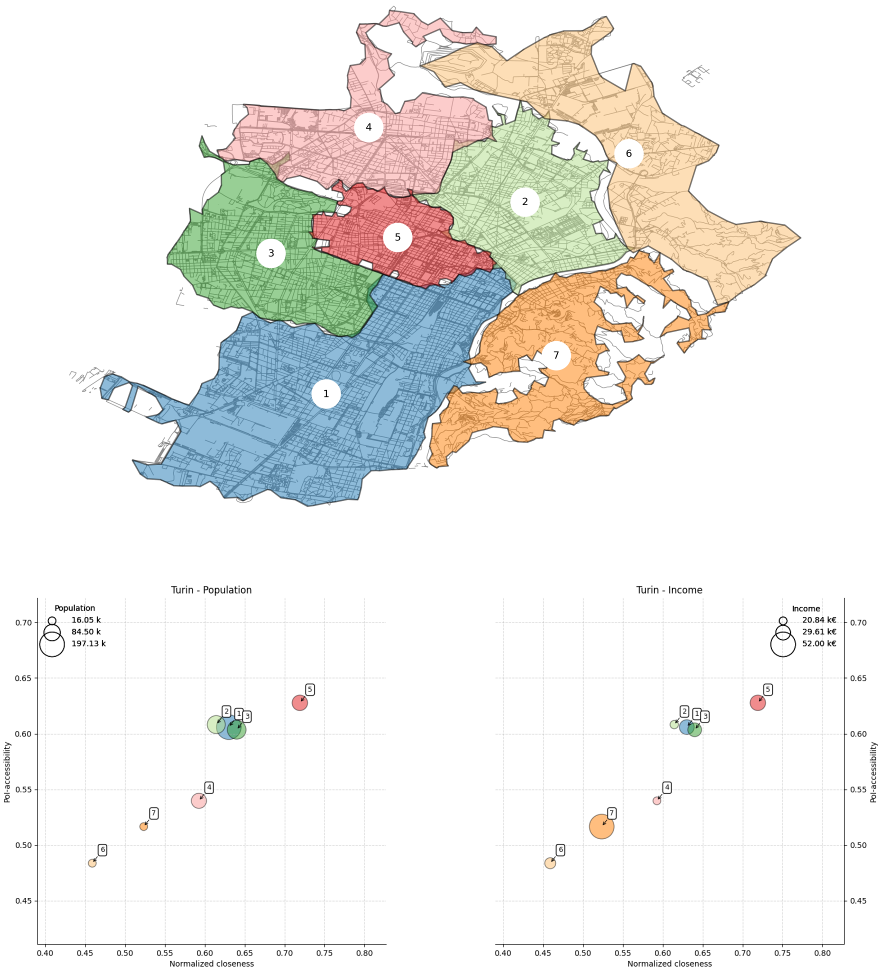

Figure 31: Turin: Infomap neighborhoods (top), PoI-accessibility vs normalized closeness aggregated by neighborhoods (bottom). The map contains information from OpenStreetMap and OpenStreetMap Foundation, which is made available under the Open Database License.
