# Supplementary material for: Understanding the interplay between urban segregation and accessibility to services with network analysis
Source: PLoS One. 2026 Apr 1;21(4):e0342156. doi: 10.1371/journal.pone.0342156 (PMC13042880; doi:10.1371/journal.pone.0342156)
Supplement: S7 Appendix — Software: Open Source code and plots https//github.com/mirkolai/cities To allow experiments replication, the source code to download data from sources, and calculate the accessibility and closeness measures is released under the GPL-3 License. At the same repo, we released all the plots used in the paper, in the Supplementary Material, and the others we could not include for the sake of brevity. (PDF) [file pone.0342156.s007.pdf]

Understanding the interplay between urban segregation and accessibility to services with network analysis: Supplementary Material

Comparing Closeness to $\mathcal{P}$, $\mathcal{D}$, and $\mathcal{E}$

In this appendix, we plot three bubble charts that show how each single component of the PoI-accessibility metric relates to normalized closeness. For simplifying the comparison between different plots, we propose here again the bubble chart of Fig. 10 of the main manuscript (here it is Fig. 1). It is striking that the three measures show quite different patterns. In particular, PoI-proximity compared to closeness (Fig. 2 is the metric that most resembles the comparison between PoI-accessibility and closeness, as also confirmed by the correlation measured with Pearson coefficient and Kendall's tau in the main paper. PoI-density's average is quite low, and points in the plot (Fig. 3 are concentrated mainly in the bottom-right corner; quite interestingly, outliers stand out on the top of the plot. Finally, PoI-entropy shows a quite different pattern, proving once more that all these measures give different viewpoints that can tell different stories on urban hyper-proximity.

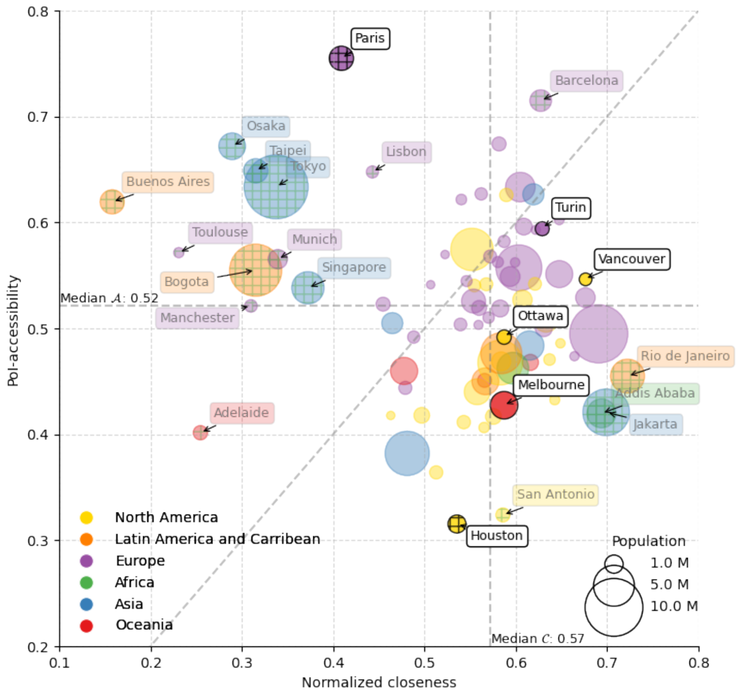

Figure 1: Bubble chart comparing cities' PoI-accessibility and normalized closeness. The markers' size is proportional to the city's population, and the color refers to the geographical region a city belongs to. Outliers according to elliptic envelope (with contamination parameter set to 0.22) are textured. Text annotations' backgrounds are colored coded with the geographical region, with an exception: if the city is one of the six representative ones, they are in white.

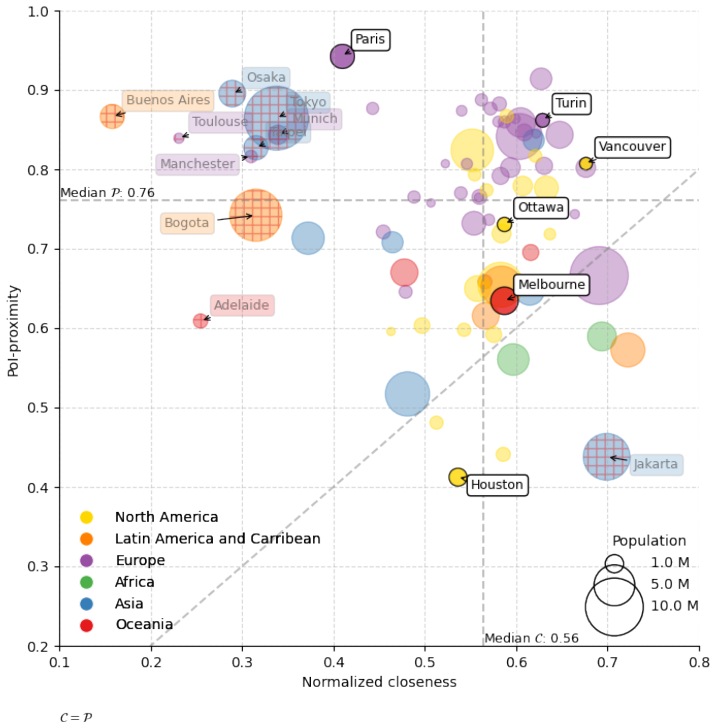

Figure 2: Bubble chart comparing cities' PoI-proximity and normalized closeness. The markers' size is proportional to the city's population, and the color refers to the geographical region a city belongs to. Outliers according to elliptic envelope (with contamination parameter set to 0.22) are textured. Text annotations' backgrounds are colored coded with the geographical region, with an exception: if the city is one of the six representative ones, they are in white.

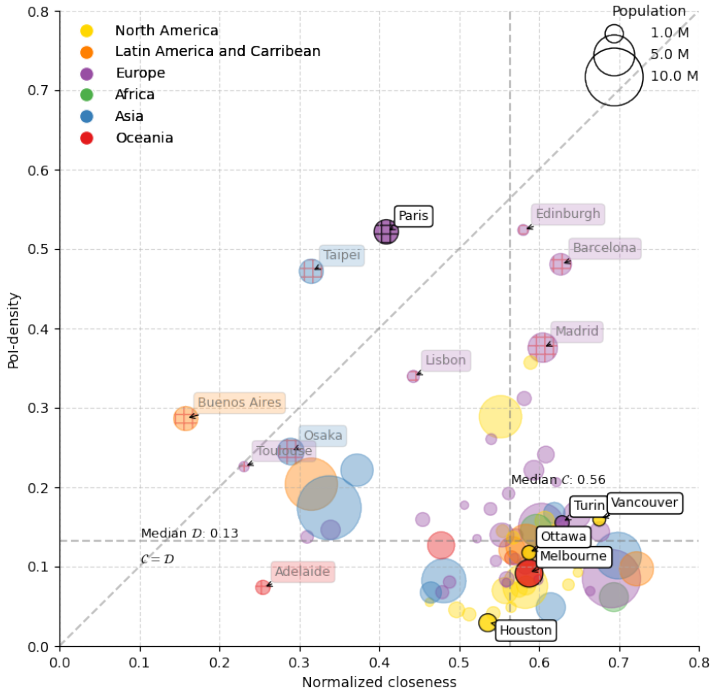

Figure 3: Bubble chart comparing cities' PoI-density and normalized closeness. The markers' size is proportional to the city's population, and the color refers to the geographical region a city belongs to. Outliers according to elliptic envelope (with contamination parameter set to 0.22) are textured. Text annotations' backgrounds are colored coded with the geographical region, with an exception: if the city is one of the six representative ones, they are in white.

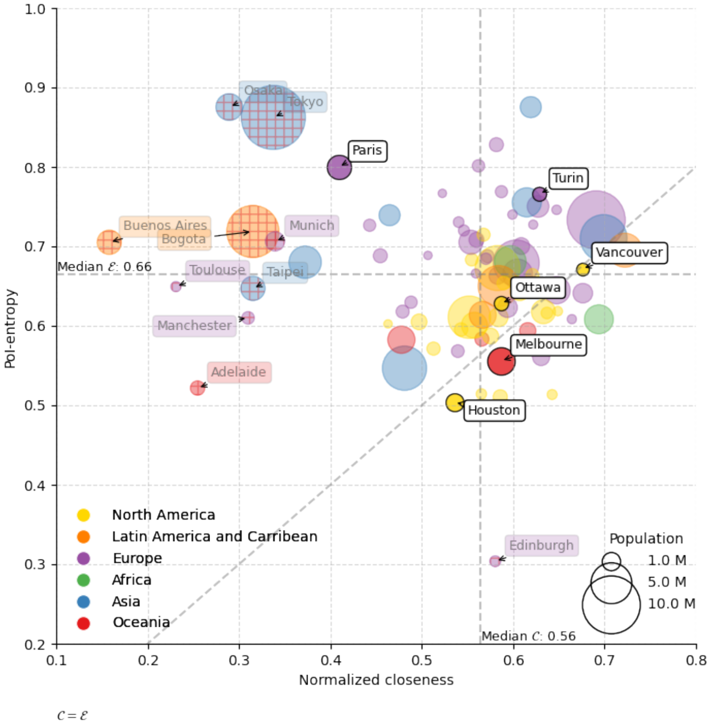

Figure 4: Bubble chart comparing cities' PoI-entropy and normalized closeness. The markers' size is proportional to the city's population, and the color refers to the geographical region a city belongs to. Outliers according to elliptic envelope (with contamination parameter set to 0.22) are textured. Text annotations' backgrounds are colored coded with the geographical region, with an exception: if the city is one of the six representative ones, they are in white.
