# Supplementary material for: Understanding the interplay between urban segregation and accessibility to services with network analysis
Source: PLoS One. 2026 Apr 1;21(4):e0342156. doi: 10.1371/journal.pone.0342156 (PMC13042880; doi:10.1371/journal.pone.0342156)
Supplement: Attachment — Submitted filename: Response to Reviewers.pdf [file pone.0342156.s008.pdf]

Rebuttal Letter

Dear Editor and Reviewers,

Thank you for your thoughtful comments and suggestions. We have carefully revised the manuscript in accordance with the feedback provided by the Reviewers and the Editor. We believe these revisions address all concerns raised and have strengthened the manuscript.

For clarity, all additions to the manuscript are marked in **blue**, and all deleted text is shown with a ~~**red strikethrough**~~.

In the following pages, we provide our detailed, point-by-point responses to all Reviewer and Editor comments. To make them easily distinguishable, our responses are written in **blue**, while the original Reviewer comments precede them in their original format.

Best regards,
the authors

Editor' comments:

When submitting your revision, we need you to address these additional requirements.

1. Please ensure that your manuscript meets PLOS ONE's style requirements, including those for file naming. The PLOS ONE style templates can be found at

https://journals.plos.org/plosone/s/file?id=wjVg/PLOSOne_formatting_sample_main_body.pdf and
https://journals.plos.org/plosone/s/file?id=ba62/PLOSOne_formatting_sample_title_authors_affiliations.pdf

We checked the coherence of our manuscript with PLOS ONE’s style requirements and edited it as follows: 1) we updated the formatting of authors names and affiliations; 2) we included the corresponding author line; 3) we changed the file extension of figures and file naming according to PLOS ONE’s convention.

2. We note that the grant information you provided in the ‘Funding Information’ and ‘Financial Disclosure’ sections do not match.

When you resubmit, please ensure that you provide the correct grant numbers for the awards you received for your study in the ‘Funding Information’ section.

We corrected the information about Fundings and Financial disclosure.

3. Thank you for stating in your Funding Statement:

[SV has been partially funded by PRIN 2022 (PNRR M4C2) EU Commission - Next Generation EU, Mission 4 Component 1 CUP C53D23005810006].

Please provide an amended statement that declares *all* the funding or sources of support (whether external or internal to your organization) received during this study, as detailed online in our guide for authors at http://journals.plos.org/plosone/s/submit-now. Please also include the statement “There was no additional external funding received for this study.” in your updated Funding Statement.

Please include your amended Funding Statement within your cover letter. We will change the online submission form on your behalf.

We followed your instructions in our new submission.

4. Thank you for stating the following in your manuscript:

[This research has been partially funded by PON “Ricerca e Innovazione” 2014-2020 716 framework, and by the European Union - Next Generation EU, Mission 4 Component 2 717 - CUP C53D23005810006, and CUP C33C24000340001.]

We note that you have provided funding information that is not currently declared in your Funding Statement. However, funding information should not appear in the Acknowledgments section or other areas of your manuscript. We will only publish funding information present in the Funding Statement section of the online submission form.

Please remove any funding-related text from the manuscript and let us know how you would like to update your Funding Statement. Currently, your Funding Statement reads as follows:

[SV has been partially funded by PRIN 2022 (PNRR M4C2) EU Commission - Next Generation EU, Mission 4 Component 1 CUP C53D23005810006]

Please include your amended statements within your cover letter; we will change the online submission form on your behalf.

According to the Editor’s points 2, 3, and 4 we have removed incorrect funding information from the manuscript and included the amended statement in the cover letter. Our final financial statement is the following:

This research has been partially funded by PON “Ricerca e Innovazione” 2014-2020 716 framework, and by the European Union - Next Generation EU, Mission 4 Component 2 717 - CUP C53D23005810006, and CUP C33C24000340001. There was no additional external funding received for this study.

5. Thank you for stating the following in your Competing Interests section:

[NO authors have competing interests].

Please complete your Competing Interests on the online submission form to state any Competing Interests. If you have no competing interests, please state "The authors have

declared that no competing interests exist.", as detailed online in our guide for authors at http://journals.plos.org/plosone/s/submit-now

This information should be included in your cover letter; we will change the online submission form on your behalf.

6. We noted in your submission details that a portion of your manuscript may have been presented or published elsewhere. Please clarify whether this conference proceeding was peer-reviewed and formally published. If this work was previously peer-reviewed and published, in the cover letter please provide the reason that this work does not constitute dual publication and should be included in the current manuscript.

[Fig. 2 has been used also in paper "A Complex Networks Approach to Evaluate the 15-Minute City Paradigm and Urban Segregation at Different Scales", to be published in the Proceedings of the International Conference on Complex Networks and Their Applications, 2024. The present submission has been invited to extend that work.]

Please clarify whether this conference proceeding was peer-reviewed and formally published. If this work was previously peer-reviewed and published, in the cover letter please provide the reason that this work does not constitute dual publication and should be included in the current manuscript.

Thank you for raising this point.

We did not anticipate this to be an issue, as the current submission was prepared following an invitation to submit an extended version of our previous work, "A Complex Networks Approach to Evaluate the 15-Minute City Paradigm and Urban Segregation at Different Scales" , for publication in your journal.

Figure 2 in the original submission had also appeared in that earlier work, which meanwhile has been published in the Proceedings of the International Conference on Complex Networks and Their Applications, held in Istanbul in 2024. However, in light of the potential licensing concerns this might raise, we have chosen to remove the figure from the manuscript, even though it was initially helpful for context. We believe the article remains clear and self-contained without it. We also added a reference to the Stephen Few classic textbook in data visualization "Now You See It: Simple Visualization Techniques for Quantitative Analysis" that contains an extended description of bubble plots as extension of scatter plots.

7. We note that Figures 1, 6, 7, 8, 11, 13, 14, 15, 16, Appendices S5 and S6 in your submission contains a map image which may be copyrighted. All PLOS content is published under the Creative Commons Attribution License (CC BY 4.0), which means that the manuscript, images, and Supporting Information files will be freely available online, and any third party is permitted to access, download, copy, distribute, and use these materials in any way, even commercially, with proper attribution. For these reasons, we cannot publish previously copyrighted maps or satellite images created using proprietary data, such as Google software (Google Maps, Street View, and Earth). For more information, see our copyright guidelines: http://journals.plos.org/plosone/s/licenses-and-copyright.

We require you to either (1) present written permission from the copyright holder to publish these figures specifically under the CC BY 4.0 license, or (2) remove the figures from your submission:

1. You may seek permission from the original copyright holder of Figures 1, 6, 7, 8, 11, 13, 14, 15, 16, Appendices S5 and S6 to publish the content specifically under the CC BY 4.0 license.

We recommend that you contact the original copyright holder with the Content Permission Form (http://journals.plos.org/plosone/s/file?id=7c09/content-permission-form.pdf) and the following text:

“I request permission for the open-access journal PLOS ONE to publish XXX under the Creative Commons Attribution License (CCAL) CC BY 4.0 (http://creativecommons.org/licenses/by/4.0/). Please be aware that this license allows unrestricted use and distribution, even commercially, by third parties. Please reply and provide explicit written permission to publish XXX under a CC BY license and complete the attached form.”

Please upload the completed Content Permission Form or other proof of granted permissions as an "Other" file with your submission.

In the figure caption of the copyrighted figure, please include the following text: “Reprinted from [ref] under a CC BY license, with permission from [name of publisher], original copyright [original copyright year].”

2. If you are unable to obtain permission from the original copyright holder to publish these figures under the CC BY 4.0 license or if the copyright holder’s requirements are incompatible with the CC BY 4.0 license, please either i) remove the figure or ii) supply a replacement figure that complies with the CC BY 4.0 license. Please check copyright information on all replacement figures and update the figure caption with source information. If applicable, please specify in the figure caption text when a figure is similar but not identical to the original image and is therefore for illustrative purposes only.

The following resources for replacing copyrighted map figures may be helpful:

USGS National Map Viewer (public domain): http://viewer.nationalmap.gov/viewer/
The Gateway to Astronaut Photography of Earth (public domain): http://eol.jsc.nasa.gov/sseop/clickmap/
Maps at the CIA (public domain): https://www.cia.gov/library/publications/the-world-factbook/index.html and https://www.cia.gov/library/publications/cia-maps-publications/index.html
NASA Earth Observatory (public domain): http://earthobservatory.nasa.gov/
Landsat: http://landsat.visibleearth.nasa.gov/
USGS EROS (Earth Resources Observatory and Science (EROS) Center) (public domain): http://eros.usgs.gov/#
Natural Earth (public domain): http://www.naturalearthdata.com/

We addressed these important concerns with the following actions.

1. The only figures that include OSM map tiles in the manuscript are Figures 1, and 7 (we use current figures numbering here). We thus modified the captions according to PLOS One guidelines (https://journals.plos.org/plosone/s/figures#loc-maps) stating that "OpenStreetMap map tiles are free to use as long as they are accompanied by the following attribution statement: “Base map and data from OpenStreetMap and OpenStreetMap Foundation”. Maps created using OpenStreetMap data must be accompanied by the following attribution statement: "Contains information from OpenStreetMap and OpenStreetMap Foundation, which is made available under the Open Database License.” We updated Fig. 1 and 7 captions accordingly.

2. All other figures displaying city maps (Figures 6, 8, 11, 12, 14, 15, and 16, we use current figures numbering), as well as the figures contained in S5, S6 and the novel S7, do not actually use any map tiles. Instead, we use the coordinate data to project intersection and street data points on a Cartesian plane thus not encountering the issue of using maps under the OSM CC BY-SA 2.0. We updated all of the related figures' captions accordingly.

3. We further queried the PLOS One editorial staff if the previous two actions suffice, and on July 17th we received the following answer from Zora Nazarei, Peer Review Operations Specialist:

> *“Before we proceed, we need some additional information to ensure the images contained in your submission comply with PLOS ONE copyright policy (https://journals.plos.org/plosone/s/licenses-and-copyright).*
>
> *PLOS ONE requires direct confirmation that all images contained in submissions can be published under the CC BY 4.0 license utilized by PLOS ONE. This license allows unrestricted reproduction and reuse (even commercial) of images published in PLOS.*
>
> *In response to your query above, **Open StreetMaps is an acceptable source and does comply with our CC BY 4.0 license.***
>
> *Please respond to the following query regarding the maps/satellite images contained in Figures 1, 6, 7, 8, 11, 13, 14, 15, 16, Appendices S5 and S6:*
>
> *- Where did the authors obtain the maps, basemaps, shapefiles, map data, etc in these figures?*
>
> *- What software was used to make these maps?*
>
> *Please provide the direct URL for the relevant webpage or webpages where your map data was retrieved. Please ensure that licensing information for the relevant data is present on the webpage(s) you provide.*
>
> *Please also update the captions for Figures 1, 6, 7, 8, 11, 13, 14, 15, 16, Appendices S5 and S6 to include any required attribution associated with the applicable license or data source.*

If we cannot confirm that your map was made using only publicly available data compatible with the CC BY 4.0 license, your map will need to be removed or replaced. If you choose to replace your maps, please update the relevant Figure captions, and provide us with information about your replacement map's data, including license information through the Editorial Manager system.

When we receive your response through Editorial Manager, we will evaluate the license associated with your map and confirm it is compatible with the CC BY 4.0 license utilized by PLOS ONE. Please be assured that we will reach out again at that time if there are any remaining issues with your submission.“

Then, we acknowledge that - as stated in the answer reported above - *Open StreetMaps is an acceptable source and does comply with our CC BY 4.0 license.* Finally, we reply below to the additional questions posed by the Peer Review Operations Specialist:

- Where did the authors obtain the maps, basemaps, shapefiles, map data, etc in these figures?

Map data, from all the figures in the manuscript and in the Supporting Information files (S5, S6 and S7), is from Open Street Map, as clarified above. Only Fig. 1 and 7 also use OSM map tiles. For all the other figures, we use the coordinate data to project intersection and street data points on a Cartesian plane, using our own software relying on the libraries listed below. All the figures' captions have been updated to make the correct references to OSM data.

- What software was used to make these maps?

The software that we used is available at https://github.com/mirkolai/cities and make use of the following python libraries to process and visualize geo spatial data:

- **osmnx** https://osmnx.readthedocs.io/en/stable/
- **pandas** https://pandas.pydata.org/
- **geopandas** https://geopandas.org/en/stable/
- **matplotlib** https://matplotlib.org/
- **folium** https://python-visualization.github.io/folium/latest/ (Fig.1 and 7 only, to visualize OSM map tiles)

We updated the Data section accordingly. It is our understanding that our usage of OSM data is permitted.

Reviewers' comments:

Reviewer #1:

Dear authors,
This study is rich and comprehensive in scope, as it examined 81 cities worldwide, providing broad insights rather than focusing on a single location.
However, there are data limitations: while openstreetmap and other public datasets are valuable, they may lack uniform coverage across all cities, potentially affecting accuracy. It is essential to discuss how these limitations could potentially skew the results or lead to misinterpretations of urban density and accessibility. A comparative analysis or a discussion on the reliability of these databases in urban studies would enhance the credibility of your findings.

We agree that while OpenStreetMap (OSM) and other public datasets enable large-scale comparative urban analysis, they are not without limitations—particularly with respect to consistency and completeness across different regions.

To address this, we have expanded the Limitations section of the manuscript to explicitly acknowledge potential coverage and quality variations in OSM data, which may influence the measurement of accessibility and the representation of urban density in certain cities.

We believe this additional discussion improves the transparency of our methodology and clarifies the potential sources of variability in our findings.

Also, the shift from the understanding of density in terms of population per square kilometer to focusing on the density of Points of Interest (POIS) within each isochrone is a significant methodological choice that requires further justification (line 177).

Thank you for pointing this out.

To clarify this point, we have expanded the manuscript to provide a stronger justification for our methodological choice and included a paragraph that elaborates on our conceptual framing.

Additionally, we have replaced the terms *'proximity'*, *'density'*, *'entropy'*, and *'accessibility'* with *'PoI-proximity'*, *'PoI-density'*, *'PoI-entropy'*, and *'PoI-accessibility'*, respectively. This change highlights the distinction between our metrics and those commonly defined in the 15-minute city literature, thereby reducing potential methodological ambiguity and ensuring our definitions are clearly understood.

To the assumption that road network intersections can serve as proxies for population locations (lines 88-94) is a substantial leap that demands a stronger theoretical foundation. It would be beneficial to situate this decision within existing literature, providing examples of previous studies that have successfully employed similar methodologies. This will address the concerns about accuracy and strengthen the argument for the methodological choices made as well.

Thank you for this important observation.

We agree that using road network intersections as proxies for population distribution requires a clearer theoretical justification. We have revised the manuscript to more thoroughly explain our methodological choice and added a dedicated paragraph contextualizing this approach within the existing literature. In this addition, we emphasize the spatial embeddedness of intersections within the urban fabric and their capacity to capture population density patterns in a realistic manner.

We believe these revisions help clarify the rationale behind our approach and strengthen its alignment with established practices in the field.

Considering the diverse range of cities studied, an in-depth analysis of how socio-cultural dynamics shape urban accessibility is essential.

We thank the reviewer for their insightful feedback. We indeed explored the relationship between socio-economic factors and urban accessibility metrics for Italian cities, where relevant data was readily available.

While a more comprehensive cross-country analysis falls beyond the scope of this work, we acknowledge this as a meaningful limitation and have expanded the discussion accordingly. In particular, we have extended the Conclusion section to address how additional data sources, such as satellite-derived night-time light intensity, housing age, and land value, can serve as proxies for income and socio-demographic status. This addition also reflects and integrates the comments raised by Reviewer 2.

It is commendable that the methodology and visual results are made publicly available on github. However, the accessibility and functionality of the provided links must be verified.

We furtherly verified that the data is publicly available at the provided links and that all links work properly.

It should be noted that listing findings within the introduction section is unconventional and potentially confusing for the reader. The introduction should set the context, define the research gap, and outline the objectives and significance of the study without delving into the results.

We have now removed the listing of findings from the Introduction section and reshaped it according to the reviewer's suggestion. For completeness, we kept the list of findings in the cover letter.

Moreover, the manuscript does not clearly articulate how categories such as Health and well-being are evaluated within the study. Providing a detailed methodology on the evaluation criteria and process will enhance the transparency and reproducibility of the research.

The categories used in this study (including *Health and well-being*) are based on a classification previously proposed by Nicoletti et al. (2023). We begin with the OpenStreetMap PoI tags and group them into broader categories according to that framework. A detailed list of the specific tags included in each category (e.g., the *Health and*

well-being category includes ‘pharmacy’, ‘dentist’, ‘clinic’, ‘hospital’, and ‘doctors’) is provided in Supplementary Material - S1.

We have revised this explanation in the Data section to improve clarity for the reader.

Reference needs to include recent studies;

We integrated references to more recent studies (including Olivari et al, 2023, Zhang et al, 2023, Teixeira et al 2024 etc.), also according to Reviewer 2 suggestions.

Recheck the reference formatting as in (Bruno M, Monteiro Melo HP, Campanelli B, Loreto V. A universal framework for inclusive 15-minute cities. Nature Cities. 2024;1:633–641.) this reference is incomplete.

We thank the reviewer for spotting the missing element in the reference. We have now added the number field which was missing from the original version.

In line 103, please mention which supplementary material you are referring to.

We have revised the manuscript accordingly and added explicit references to the relevant sections of the Supplementary Material whenever mentioned.

Lastly, avoid using jargon such as "ghettoization" without providing a definition, and if "segregation" suffices, refrain from overusing it. Alternatively, on page 10, line 18, the phrase "HERE, WE introduce" needs more formal technical writing. On page 30, line 595, a clearer trend emerges. Avoid including extraneous comments such as "Taken together" in the abstract.

While the provided references to pages and line numbers do not map to the highlighted sentences, we looked for matches in the text and revised the manuscript according to the suggestions: 1) we removed the use of “ghettoization”, 2) we changed the wording in the abstract and removed similar instances from the manuscript, and 3) we used more formal writing where indicated.

Reviewer #2:

This manuscript offers a socially relevant and technically rigorous investigation that examines the relationship between socio-spatial segregation and urban service accessibility within the context of the 15-minute city paradigm. The authors implement a robust methodology that is based on network science and utilises PoI proximity, density, entropy, and closeness centrality in 92 global cities.
Furthermore, it emphasises the study's methodological coherence and innovation. The authors offer a comprehensive comprehension of the ways in which infrastructural accessibility does not always translate into equitable connectivity by integrating node-based accessibility metrics with normalised closeness centrality within a multi-scale spatial framework.

This effectively expands upon previous computational urbanism research (e.g., Hillier & Hanson, 1984; Porta et al., 2006) while also building upon the scope of earlier endeavours

such as Bruno et al. (2020) and Nicoletti et al. (2021). The decision to model city clusters using Infomap instead of administrative boundaries is particularly commendable, as it provides a more naturalistic representation of urban morphology.

Furthermore, the investigation is exceptional in its transparency and openness to scientific inquiry. The authors have granted complete access to their code repositories, which encompass scripts for data retrieval, processing, and visualisation. The methodological credibility is improved by the clear description of the use of cloud-based batch processing for scaling computational tasks across large networks.

We thank the reviewer for the positive feedback.

However, there are a few areas that could benefit from specific improvements:

Limitations of Socioeconomic Coverage: Although the sociodemographic validation of Italian cities by income data is valuable, the conclusions regarding urban segregation may appear to be restricted in their generalisability. The authors are encouraged to recognise the potential of alternative proxies (e.g., night-time light data, housing age, or land value, as demonstrated in Bilal et al., 2019; Venerandi et al., 2018) in situations where income data are not readily accessible. This would enhance the paper's relevance to cities in the Global South that have incomplete datasets.

We thank the reviewer for the thoughtful feedback regarding the limitations of socioeconomic coverage in our study. We appreciate the opportunity to clarify and expand on this important aspect.

We indeed recognize that while we find a signal related to urban segregation, the study only revolves around Italian cities because of data availability constraints. Income and sociodemographic information have however been found to correlate with other information, which can be successfully used as a proxy for income.

We extended our discussion to include these aspects in the Conclusions section.

Clarification of Terminology: The manuscript could benefit from a more distinct differentiation between network-based disconnection and spatial segregation. Although closeness centrality is a valuable proxy for topological isolation, it may not fully capture the sociopolitical aspects of urban exclusion. Interpretive nuance could be improved by contextualising this within the context of established urban inequality literature (e.g., Lees, 2017; Lucas, 2012).

We have now clarified in the Methods section that while closeness centrality serves as a proxy for topological isolation, we use it with caution as an indicator of urban segregation. We explicitly acknowledge its limitations in capturing the full complexity of urban exclusion, which includes sociopolitical and economic dimensions.

We have now contextualized our approach within the relevant literature on urban inequality and more clearly articulated the distinction between structural connectivity and broader forms of urban segregation. To this aim, we have incorporated the suggested references,

drawing in particular on Lees (2008), as we did not identify a clear source for the cited Lees (2017). We believe this revised framing adds important interpretive nuance to our use of the metric.

Comparative Literature Positioning: In addition to comparisons with Nicoletti and Bruno, this study could be more clearly positioned within the emerging global discourse on walkability and equity by including references to recent work on accessibility-diversity metrics (e.g., Kim & Kang, 2020; Welle et al., 2018).

We thank the reviewer for this valuable suggestion. While we were unable to locate academic publications corresponding exactly to the references "Kim & Kang (2020)" and "Welle et al. (2018)," we fully agree on the importance of contextualizing our study within the emerging global discourse on walkability and accessibility equity.

To address this, we have identified and incorporated recent peer-reviewed studies (Kang et al., 2021; Yin et al., 2024; He et al., 2025) and provided a more detailed discussion of two particularly relevant contributions (Van Wee, 2022; Lucas et al., 2016). These works have now been cited in the manuscript's introduction to better position our accessibility-diversity metrics within this emerging global discourse.

Policy Implication Framing: Although the empirical results are robust, the discussion could be enriched to provide a more precise explanation of the implications for urban planning. In particular, how could planners or policymakers employ these metrics to revise zoning practices, promote transit equity, or prevent service clustering? The framework is consistent with the call for spatial justice and should be more explicitly linked to such agendas.

In the conclusion section, we have expanded the discussion to address the implications of how policymakers can employ these metrics—particularly in relation to spatial justice—and have more explicitly connected them to our platform, outlining potential applications and directions for future work.

Specifically, we now highlight how the framework can support evidence-based planning by identifying underserved areas and guiding more equitable resource allocation, in accordance with the 11th goal of the United Nations 2030 Agenda for Sustainable Development "Make cities and human settlements inclusive, safe, resilient and sustainable" (https://sdgs.un.org/goals/goal11). Additionally, we have introduced future developments of the platform aimed at evaluating system robustness and intervention scenarios to strengthen urban accessibility.

Visual Simplification: A few figures, particularly bubble charts and heatmaps, are dense and could be more clearly annotated. Highlighting extreme or illustrative cases (e.g., Paris, Houston, Jakarta) would assist in interpretation.

We addressed this concern with the following three actions.

First, we have highlighted the annotation boxes of the six representative cities reported in Table 3 to make them easier to identify in Fig. 9 (now Fig. 10). The figure caption has also been updated accordingly. Now outliers discussed in the text are highlighted with an

annotation with white background, while the 6 sample cities are highlighted with a light gray background.

Second, figures 11 and 12 have been restructured to better illustrate the comparison between PoI-accessibility and normalized closeness using bubble charts for the six representative cities, at two different spatial resolutions (i.e. neighborhoods and census areas). Each row in the figures now corresponds to one representative city: Paris, Turin, and Vancouver in Fig. 11, and Ottawa, Melbourne, and Houston in Fig. 12. For each city, the layout includes a city map displaying neighborhoods (clusters) identified via Infomap, along with two bubble charts—one for neighborhoods and one for census areas. A consistent color scheme is used to represent the clusters, helping to visually link the neighborhoods and census areas to their locations on the city map. While this arrangement enhances spatial understanding, it still preserves the ability to compare accessibility patterns both within and across cities.

Finally, we have divided the experimental analysis into five subsections to better contextualize figures discussion and interpretation.

A comparative schematic explaining how PoI proximity, density, and entropy relate conceptually to accessibility versus closeness would also aid clarity.

We addressed this concern with the following three actions.

First, we added a schematic table in the Methods section to summarize the accessibility and connectivity metrics used in our analysis.

Second, we added three bubble charts highlighting how proximity, density, and entropy relate to closeness together with a discussion of differences and similarities to the Supplementary Material (Appendix S7).

Finally, we added two correlation heatmaps (now Fig. 5) to show how all metrics in the main manuscript are related to each other.

Language and Presentation: The manuscript is generally well-written. A few minor edits are required to ensure that Figure captions are clear. These issues may be resolved through revision.

We have revised all captions in the manuscript to ensure their clarity for the reader. In particular, we added bold titles, restructured the descriptions of panels, and standardized the placement of OpenStreetMap attributions.
