# Supplementary material for: Understanding the interplay between urban segregation and accessibility to services with network analysis
Source: PLoS One. 2026 Apr 1;21(4):e0342156. doi: 10.1371/journal.pone.0342156 (PMC13042880; doi:10.1371/journal.pone.0342156)
Supplement: Attachment — Submitted filename: Response to Reviewers_v2.pdf [file pone.0342156.s009.pdf]

Rebuttal Letter

Dear Editor and Reviewers,

Thank you for your thoughtful comments and suggestions. We have carefully revised the manuscript in accordance with the feedback that you provided. We believe these revisions address all concerns raised and have strengthened the manuscript.

For clarity, all additions to the manuscript are marked in **blue**, and all deleted text is shown with a ~~**red strikethrough**~~.

In the following pages, we provide our detailed, point-by-point responses to all Reviewer and Editor comments. To make them easily distinguishable, our responses are written in **blue**, while the original Reviewer comments precede them in their original format.

Best regards,
the authors

Reviewer #2:

This manuscript presents a strong and timely contribution to urban studies by investigating the interplay between accessibility to services and segregation within the framework of the "15-minute city." The multi-scalar, network-based approach, which combines measures of Points of Interest (PoI) proximity, density, entropy, and closeness centrality across 92 cities worldwide, is a major strength. The methodological innovation, particularly the use of community detection and transport connectivity analysis, advances computational urbanism and contributes to the literature on spatial justice. The authors' efforts to improve the manuscript based on earlier reviews are evident, and the expanded methodological clarity, updated figures, and broader literature engagement substantially strengthen the paper.

That said, a few areas merit further attention to enhance clarity, rigor, and impact:

Data Transparency and Limitations: While the manuscript now acknowledges the limitations of OpenStreetMap and other open datasets, further discussion on how heterogeneous data quality across regions may skew results would increase transparency. For instance, how do results differ for Global South cities where transport data is sparse compared to Europe or North America? A sensitivity check or comparison with alternative proxies (night-time light data, land value, housing age) could better demonstrate robustness.

We thank the reviewer for acknowledging our efforts to revise the manuscript. We agree that service and connectivity heterogeneity are well-documented in urban studies, and that alternative proxies can explain differences across diverse political and geographical contexts. In particular, the literature acknowledges that taxation is a key proxy for service quality; we have now added a brief discussion on this point. However, we emphasize that our primary contribution is a quantitative methodology for identifying relationships between service

accessibility and transport connectivity. To better clarify this, we have expanded the 'Objectives and Scope' and 'Limitations and Future Perspectives' sections.

Furthermore, we agree that heterogeneous data quality is a potential source of bias. We have clarified that OSM completeness does not follow a simple Global North–Global South divide but varies substantially within regions and income groups. We now cite Barrington-Leigh and Millard-Ball (2017) to strengthen our discussion on robustness. We frame satellite-derived night-time light, housing age, and land value as complementary proxies for future sensitivity analyses in data-sparse contexts. While a full sensitivity analysis remains beyond the scope of this study, we now explicitly frame this as a priority for future work.

Similarly, the weighting scheme for the combined accessibility score (equal weights to proximity, density, entropy) may benefit from a short justification or robustness test showing whether alternative weightings significantly affect outcomes.

As mentioned in our previous answer, our approach distinguishes itself from others, such as those by [10] and [11], by incorporating multiple metrics rather than relying on a single measure. We additionally reference a recent study that reviews proximity assessment methodologies, particularly in the context of urban planning models like the 15-minute city [27].

We welcome the suggestion to assess the sensitivity of our results to the weighting scheme. Hence, we conducted a check by comparing the rankings obtained with equal weights (1/3 each) against those obtained using different weight configurations. The results of this analysis are presented in Fig. 3 of Appendix 2 (in the Supplementary Material), where we use a triangular heatmap to illustrate how the rankings change when varying the weights. This analysis reinforces the overall robustness of the rankings across different weight choices, showing strong or very strong signals of correlations with the ranking resulting from equal weighting. The figure is also reported below, and an additional brief comment has been added to Section “Analysis of the accessibility Metrics”, after Fig. 7.

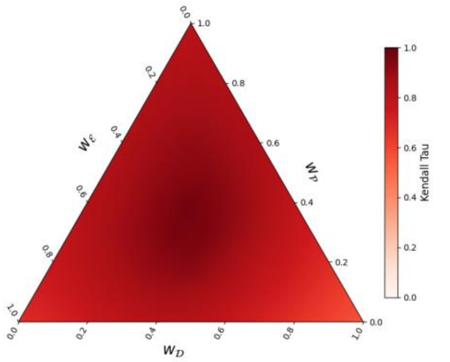

Interpretive Nuance on Segregation: While closeness centrality is a useful proxy for topological isolation, it risks oversimplifying socio-spatial exclusion. The revised text acknowledges this, but the interpretation of “segregation” should be carefully phrased to avoid overextension. Integrating insights from the literature on urban inequality (e.g., Lees,

Lucas, Soja) can help balance the structural-network perspective with the broader social dimension.

In the first round of review, we added references to situate our approach within the relevant literature on urban inequality, as suggested by Reviewer 2. Specifically, we cited Lees (2008) (since we could not identify a clear source for the suggested Lees 2017), as well as Lucas (2012, 2016) and Soja (2013). In this round, we have further strengthened our contextualization by including an additional reference to Soja (2009) and clarifying that our work emphasizes spatial segregation rather than other forms of inequality. Accordingly, we have revised the text to explicitly highlight our focus on spatial segregation, topological isolation, and mobility connectivity in cities. We believe these revisions fully address the suggestion to integrate insights from the literature on urban inequality while clearly situating our study within the spatial dimension of these mechanisms.

Results and Communication: The restructuring of figures and annotations has improved readability, but some visualizations (bubble charts, heatmaps) remain dense. Highlighting key illustrative cases such as Paris, Houston, or Jakarta, within the main text would make findings more accessible. The inclusion of a schematic overview of how the different accessibility metrics interrelate is very helpful and **could be emphasized more clearly in the discussion**.

Following the suggestions of Reviewer 2, who had already raised this point in the first round of revisions, we have further improved the clarity and interpretation of our visualizations. In particular, as mentioned in a previous answer, we included Fig. 3 in Appendix 2 to provide a clearer and more intuitive representation of the relationships among the different metrics. Importantly, we now explicitly discuss these results in the newly added Discussion section, where we also compare our findings with related work and outline their potential policy implications, in line with the reviewer's following comment.

Policy Relevance: The discussion has been expanded to touch upon spatial justice and SDG 11, which is commendable. Still, the paper would benefit from more concrete guidance for policymakers and urban planners. For example, how might results inform zoning reforms, equitable distribution of public services, or resilience planning? Providing specific examples or scenarios would strengthen the applied contribution of the work.

In response to the suggestion, we have added specific examples in a new paragraph at the end of the Discussion section under the heading "Policy Applications". We now describe recommendations for policymakers and urban planners, with a focus on zoning reforms, equitable allocation of public services, and resilience planning. In doing so, we demonstrate concrete ways our findings and our platform United-and-Close can inform decision-making.

Language and Style: The manuscript is generally well-written, but a final round of language polishing would improve flow and readability. Avoid jargon such as "ghettoization" (as already addressed) and maintain a consistent formal tone. Some sentences in the abstract and introduction can be streamlined to avoid repetition.

We have carefully revised the manuscript to improve the language and ensure a consistent formal tone throughout. We have also streamlined sentences in the abstract and reshaped the introduction section to avoid repetition and enhance readability.

Reviewer #3:

This study investigates the relationship between walkable accessibility to services and urban transport connectivity across 92 global cities, filling a critical gap at both practical and theoretical levels: while the 15-minute city has been widely promoted as a sustainable urban planning strategy, its potential impacts on spatial segregation have remained insufficiently explored. The research methodology is generally rigorous and innovative, avoiding the limitations of single-indicator assessments. After a round of revisions, the logic and methodology of the article have become more refined, and it is no longer possible for me to identify major revisions that have not yet been proposed within the limited review time.

Although this article may still face some structural and fundamental questions, such as the validity of using 15 minute cities to measure segregation and equality, and the reliability of OSM data as an international comparison, whether the value judgment of segregation is universally applicable, and so on. However, the existence of these limitations is acceptable in an independent paper.

Thank you for your valuable feedback. In response to your suggestion and to Reviewer 2's comments, we have expanded the Limitations and Future Perspectives section in the revised manuscript to clarify the variability in OpenStreetMap (OSM) completeness. We also refer to another study [42] that, despite uncovering the presence of regional disparities in data coverage, reports that OSM is approximately 80% complete globally, with over 40% of countries fully mapped at the street network level.

All maps in one figure should provide a scale reference, or in the same scale. Because 1. the urban scale may have an impact on the conclusions; 2. In Figures 6, 7, and 8, all the displayed cities appear to have similar scales. At this point, the "display weights" of analysis units with the same granularity in different cities may vary significantly, leading to a greater risk of misreading.

We thank the reviewer for raising this important point regarding scale and normalization in the maps, which is indeed critical for fair visual comparison across cities. Our approach is as follows:

- **Figures comparing cities at the same granularity** (formerly Figs. 6 and 8, now Figs. 7 and 9) use a consistent scale reference and normalized values. For example, PoI-accessibility (Fig. 7) is calculated as a linear combination of normalized proximity, density, and entropy (Eq. 1). Each dot represents values computed for the corresponding 15-minute isochrone, ensuring that visual differences reflect meaningful, comparable differences between cities.

- **Closeness maps (Fig. 9)** reflect the connectivity of individual intersections and surrounding residential addresses. To allow comparisons across cities of different sizes, closeness is normalized by the city’s maximum value (Eq. 3). This ensures, for instance, that a 45-minute travel across Paris is correctly represented relative to a smaller city. The same normalization principle applies to the comparative density diagrams (Figs. 11 and 14).

- **Figures at a finer urban granularity within a single city** (Figs. 12, 13, 15–17) are exceptions. Here, we retain consistent scales but focus on comparisons **within** a city rather

than across cities. Axes ranges are adjusted to highlight heterogeneity among Infomap neighborhoods. To clarify this, we added to all relevant figure captions: *"Axes are ranged differently to simplify the comparison between neighborhoods within a city."*

This strategy ensures that visual comparisons are fair and meaningful, whether across or within cities, while making differences in scale and granularity explicit to the reader.

Although this study has innovated in both urban science and computer technology, demonstrating good process and technology, the analysis of its results is somewhat insufficient. I hope to include a separate chapter in the conclusion section, or before, to present the sociological or urban science conclusions of this study after completing international and domestic comparisons. The existing conclusions are scattered in the analysis of various chapters and tables, resulting in poor readability*.

In response to the reviewer's comment, we reorganized the manuscript to enhance readability. All discussion points previously presented in the Results section have been moved to a separate Discussion section, where we discuss the main findings, as suggested. Within this section, we added a dedicated paragraph entitled *"Final Notes on the Socio-Demographic Analysis"* to present the urban science and sociological conclusions of the study. Finally, the Conclusions section has been shortened to provide a concise summary of the key findings, as requested by Reviewer 4.

Reviewer #4:

Manuscript needs revision in structure as follows
1. Kindly add separate section on the objectives of the study after introduction section clearly stating the objectives of the study along with scope and limitations.

We thank the reviewer for this and the following comments. In response, we added an "Objectives and Scope" section after the Introduction outlining the main goals and scope of our work. We also created a separate section at the end of the manuscript entitled "Limitations and future perspectives," in which we discuss the limitations of this study, as further detailed below.

2. In section on methods add information clearly stating different stages of research and giving information on methods of data collection and analysis to achieve the objectives of study, support it with figure also.

At the beginning of the Methods section, we added a description of the different stages of the study, i.e., data collection, network construction, data analysis, and information visualization, explicitly outlining the overall workflow of the study. We also included a figure (Fig. 1) that visually summarizes this workflow and illustrates how the different stages are connected, as requested.

3. Support conclusion with data from Analysis section also do not add citations in conclusion section*

We modified the Conclusion by integrating our findings, as requested by the reviewer and Reviewer 3, and by avoiding citations. We also revised the manuscript structure by adding a Discussion section, followed by a dedicated “Limitations and Future Perspectives” section. The Conclusions were correspondingly shortened and now provide a concise summary of the main findings supported by data from the analysis.
